# Unique adaptations in neonatal hepatic transcriptome, nutrient signaling, and one-carbon metabolism in response to feeding ethyl cellulose rumen-protected methionine during late-gestation in Holstein cows

**DOI:** 10.1186/s12864-021-07538-w

**Published:** 2021-04-17

**Authors:** Valentino Palombo, Abdulrahman Alharthi, Fernanda Batistel, Claudia Parys, Jessie Guyader, Erminio Trevisi, Mariasilvia D’Andrea, Juan J. Loor

**Affiliations:** 1 Dipartimento Agricoltura, Ambiente e Alimenti, Università degli Studi del Molise, via De Sanctis snc, 86100 Campobasso, Italy; 2 Department of Animal Sciences and Division of Nutritional Sciences, University of Illinois, Urbana, IL 61801 USA; 3 Department of Animal Production, College of Food and Agriculture Sciences, King Saud University, Riyadh, 11451 Saudi Arabia; 4 Department of Animal, Dairy and Veterinary Sciences, Utah State University, Logan, UT 84322 USA; 5 Evonik Operations GmbH, Hanau-Wolfgang, 63457 Essen, Germany; 6 Department of Animal Sciences, Food and Nutrition (DIANA), Università Cattolica del Sacro Cuore, 29122 Piacenza, Italy

**Keywords:** Calf, Epigenetics, Methyl donor, Nutritional programming

## Abstract

**Background:**

Methionine (Met) supply during late-pregnancy enhances fetal development in utero and leads to greater rates of growth during the neonatal period. Due to its central role in coordinating nutrient and one-carbon metabolism along with immune responses of the newborn, the liver could be a key target of the programming effects induced by dietary methyl donors such as Met. To address this hypothesis, liver biopsies from 4-day old calves (*n* = 6/group) born to Holstein cows fed a control or the control plus ethyl-cellulose rumen-protected Met for the last 28 days prepartum were used for DNA methylation, transcriptome, metabolome, proteome, and one-carbon metabolism enzyme activities.

**Results:**

Although greater withers and hip height at birth in Met calves indicated better development in utero, there were no differences in plasma systemic physiological indicators. RNA-seq along with bioinformatics and transcription factor regulator analyses revealed broad alterations in ‘Glucose metabolism’, ‘Lipid metabolism, ‘Glutathione’, and ‘Immune System’ metabolism due to enhanced maternal Met supply. Greater insulin sensitivity assessed via proteomics, and efficiency of transsulfuration pathway activity suggested beneficial effects on nutrient metabolism and metabolic-related stress. Maternal Met supply contributed to greater phosphatidylcholine synthesis in calf liver, with a role in very low density lipoprotein secretion as a mechanism to balance metabolic fates of fatty acids arising from the diet or adipose-depot lipolysis. Despite a lack of effect on hepatic amino acid (AA) transport, a reduction in metabolism of essential AA within the liver indicated an AA ‘sparing effect’ induced by maternal Met.

**Conclusions:**

Despite greater global DNA methylation, maternal Met supply resulted in distinct alterations of hepatic transcriptome, proteome, and metabolome profiles after birth. Data underscored an effect on maintenance of calf hepatic Met homeostasis, glutathione, phosphatidylcholine and taurine synthesis along with greater efficiency of nutrient metabolism and immune responses. Transcription regulators such as *FOXO1*, *PPARG*, *E2F1*, and *CREB1* appeared central in the coordination of effects induced by maternal Met. Overall, maternal Met supply induced better immunometabolic status of the newborn liver, conferring the calf a physiologic advantage during a period of metabolic stress and suboptimal immunocompetence.

**Supplementary Information:**

The online version contains supplementary material available at 10.1186/s12864-021-07538-w.

## Background

Maternal nutrient and metabolic stresses during pregnancy are important factors that can affect fetal and neonatal growth, development [1, 2], as well as metabolic and inflammatory responses of the offspring [3]. In dairy cattle, the last two months of gestation are the most-critical period for calf fetal growth, since most of the muscle and adipose tissue formation takes place primarily during this time. Around parturition, due to normal decreases in feed intake, cows are exposed to negative energy and essential amino acid (AA) balance, hence, AA such as methionine (Met) become limiting for both cow, fetus, and/or new-born calf [4, 5].

In addition to being a substrate for protein synthesis, Met is a key component of one-carbon metabolism [6] where it is initially converted into S-adenosylmethionine (SAM), the major biological methyl donor [7] and where it is involved, through the transsulfuration pathway, in the synthesis of antioxidants glutathione (GSH) and taurine [8, 9]. Physiologically, animals not only obtain Met from the diet, but also from protein breakdown and re-methylation of homocysteine in the Met cycle [10]. Through synthesis of SAM, methyl donors may alter gene transcription in the offspring by methylating DNA and RNA [11, 12]. In non-ruminants, it was demonstrated that maternal methyl donor supplementation (i.e. betaine) led to epigenetic changes that increased expression of genes controlling hepatic gluconeogenesis in the neonate [13, 14].

Epigenetic control of gene expression, also induced by the intrauterine environment, is one of the underlying mechanisms of the ‘Fetal programming’ hypothesis [15, 16] that was first proposed by Barker in 1998 [17]. This concept seeks to explain the effect of maternal nutrition on long-term offspring growth and health [18, 19]; despite its importance, few studies in dairy cattle have addressed the role of nutrient manipulation during late-gestation on fetal and postnatal development. In this regard, for example, Met supplementation is long recognized in dairy cattle as an effective approach to improve productive performance [20, 21], but only recently a promising path in research related to the maternal effect of Met supply on calf health, immune function, and reproductive performance has been highlighted [22]. In particular, it was recently demonstrated that rumen-protected Met (RPM) supplementation during the periparturient period enhanced dry matter intake (DMI) leading to reduced incidence of metabolic disorders and improved overall cow health [23, 24]. Furthermore, enhancing Met supply during late-pregnancy upregulated mRNA abundance of AA and glucose transporters in cow placenta [25], and was also associated with changes in hepatic one-carbon metabolism and transsulfuration in calf liver [26]. Although the greater DM intake during the last 2–3 wk. prior to parturition that has been consistently reported in cows fed RPM could explain a portion of the greater body mass of the calves at birth [25, 26], other mechanisms potentially encompassing nutrient assimilation efficiency likely play a role.

There are strong associations between Met supplementation during late-pregnancy and body weight and immune response in calves [27], confirming evidence that AA can affect regulation of metabolic pathways to sustain the immune response against pathogens [28]. The potential role of methyl donors in the early-life innate immune response was recently reported in calves born to cows with high body condition score and after ex vivo lipopolysaccharide challenge [29]. Furthermore, single gene expression studies have suggested that enhancing maternal supply of Met could promote the calf’s ability to quickly adapt to extrauterine life [10, 30, 31]. Lastly, Met supplementation as RPM altered the transcriptome of bovine preimplantation embryos harvested at 70 days postpartum [32]. Although these findings provided some evidence that methyl donors could play a role in nutritional programming in dairy cows, knowledge of the underlying mechanisms between late-gestation methyl donor supply and fetal programing in dairy cattle is still in its infancy.

Since nutritional management of modern dairy cows entails dietary manipulation of energy density and nutrients such as essential AA during the last stages of gestation (~ 4–6 weeks prepartum) [9], a deeper investigation of the biological outcomes on the neonatal calf is warranted. Particularly considering possible contributions of maternal nutrition to the calf’s immune and sanitary challenges during their first weeks of life [33]. In this context, the use of RNA sequencing technology (RNA-Seq) has already proven to be a promising tool in helping us detect offspring genome-wide alterations in response to maternal post-ruminal Met supply [32].

In the present work, a subset of calves from a larger cohort [24, 27] was used to investigate the effect of maternal post-ruminal Met supply during late-pregnancy (− 28 ± 2 d to parturition) on changes in plasma systemic physiological indicators, transcriptome profiles, DNA methylation, one-carbon metabolism enzyme activities and protein abundance of nutrient-sensing pathways in the liver of new-born calves. Our general hypothesis was that Met supplementation as RPM during late-gestation improves liver immunometabolic functions in the offspring similar to those observed in the cow [34], in particular affecting key metabolic and immunological pathways such as one-carbon metabolism and transsulfuration reactions.

## Results

### Growth performance, blood biomarkers and AA concentrations in plasma

At birth, calves born to dams fed MET had greater hip height (*P* value = 0.04) and wither height (P value = 0.01). No significant differences were detected for body weight and length, and hip width (*P* value ≥ 0.10) (Table 1). Calves in MET (from cows fed additional Met) had a tendency for lower concentration of glucose compared with CON (control) calves (*P* value = 0.08) at day 2, whereas no significant differences were detected for other blood parameters (Table 2). In this regards, it is interesting to note that no significant differences were detected for insulin concentrations (Table 2). At day 2, there was no significant effect of maternal diet for any AA concentration in the plasma; however, there was a tendency for higher concentrations of Phenylalanine (Phe; *P* value = 0.08) and Taurine (Tau; *P* value = 0.06) in MET calves compared with CON (Table 3).

**Table 1:** Developmental parameters at birth in Holstein calves (*n* = 6/group) born to cows randomly assigned to receive a basal control (CON) diet from − 28 ± 2 d to parturition [1.47 Mcal/kg dry matter (DM) and 15.3% crude protein (CP)] with no added Met or CON plus ethyl cellulose Met (MET, Mepron®, Evonik Nutrition & Care GmbH, Germany)

Item	Maternal diet		SEM	***p***-value
	CON	MET		
Body length (cm)	108.58	112.20	1.05	0.10
Body weight (kg)	41.77	43.81	1.24	0.44
Hip height (cm)	78.02	82.08	1.00	0.04
Hip width (cm)	15.92	16.14	0.33	0.75
Wither height (cm)	74.48	78.77	0.90	0.01

**Table 2:** Blood plasma biomarkers at d 2 of age in Holstein calves (*n* = 6/group) born to cows randomly assigned to receive a basal control (CON) diet from − 28 ± 2 d to parturition [1.47 Mcal/kg dry matter (DM) and 15.3% crude protein (CP)] with no added Met or CON plus ethyl cellulose Met (MET, Mepron®, Evonik Nutrition & Care GmbH, Germany)

Item	Maternal diet		SEM	*p*-value
	CON	MET		
Glucose (mmol/L)	9.34	8.12	0.35	0.08
Cholesterol (mmol/L)	0.96	0.89	0.07	0.64
Urea (mmol/L)	3.32	3.56	0.24	0.62
Ca (μmol/L)	3.08	2.89	0.07	0.22
P (μmol/L)	2.39	2.27	0.05	0.29
Mg (μmol/L)	1.00	0.96	0.03	0.59
Na (μmol/L)	145.98	147.10	0.87	0.55
K (μmol/L)	4.82	5.44	0.19	0.12
Cl (μmol/L)	99.38	98.92	0.72	0.76
Zn (μmol/L)	10.32	9.78	1.84	0.89
Ceruloplasmin (μmol/L)	0.99	1.01	0.12	0.93
Albumin (g/L)	26.64	27.86	0.47	0.21
AST (U/L)	112.85	128.47	10.49	0.49
GGT (U/L)	1,344.67	1,406.67	145.77	0.84
Bilirubin (μmol/L)	14.73	16.89	1.71	0.55
NEFA (mmol/L)	0.28	0.34	0.05	0.60
Hydroxybutyrate (mmol/L)	0.05	0.09	0.01	0.08
CREA (μmol/L)	107.92	111.67	4.81	0.72
Paraoxonase (U/mL)	12.27	13.02	1.15	0.76
ROM (mg H_2_O_2_/100 mL)	13.14	12.86	0.75	0.87
FRAP (μmol/L)	164.50	171.50	8.31	0.70
NO_x_ (μmol/L)	194.00	239.33	22.31	0.33
NO_2_^−^ (μmol/L)	16.87	10.33	2.56	0.26
NO_3_^−^ (μmol/L)	156.50	229.00	24.06	0.14
Insulin (μg/L)	1.05	0.70	0.16	0.29

**Table 3:** Plasma AA concentration at d 2 of age in Holstein calves (n = 6/group) born to cows randomly assigned to receive a basal control (CON) diet from −28 ± 2 d to parturition [1.47 Mcal/kg dry matter (DM) and 15.3% crude protein (CP)] with no added Met or CON plus ethyl cellulose Met (MET, Mepron®, Evonik Nutrition & Care GmbH, Germany)

Item (μM)	Maternal diet		SEM	***p***-value
	CON	MET		
Met	0.55	0.74	0.15	0.53
Lys	1.26	1.16	0.23	0.84
Thr	1.67	1.52	0.22	0.77
Arg	1.97	1.60	0.41	0.69
Ile	1.17	1.02	0.15	0.65
Leu	1.99	1.94	0.18	0.90
Val	2.89	3.39	0.30	0.41
His	1.85	2.46	0.23	0.21
Phe	0.46	0.66	0.06	0.08
Gly	1.33	1.72	0.16	0.24
Ser	0.80	1.03	0.11	0.29
Pro	2.22	2.68	0.35	0.52
Ala	2.77	2.26	0.36	0.55
Asp	0.31	0.09	0.10	0.37
Glu	1.36	1.19	0.24	0.77
Tau	0.66	1.09	0.12	0.06
Asn	0.59	0.72	0.11	0.58
Gln	5.37	7.17	0.96	0.37
Citr	0.95	1.08	0.13	0.63
Aabu	0.08	0.10	0.01	0.56
Tyr	0.97	1.30	0.18	0.39
Orn	0.48	0.57	0.10	0.68
1-Mhis	0.47	0.59	0.05	0.30
3-Mhis	0.11	0.12	0.03	0.75

### Global DNA methylation and western blotting

**Fig. 1:**
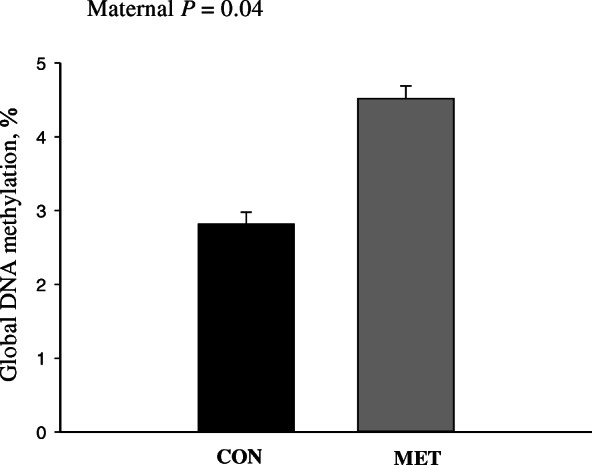
Global DNA methylation in liver tissue of 4-d old Holstein calves (*n* = 6/group) born to cows randomly assigned to receive a basal control (CON) diet from − 28 ± 2 d to parturition [1.47 Mcal/kg dry matter (DM) and 15.3% crude protein (CP)] with no added Met or CON plus ethyl cellulose Met (MET, Mepron®, Evonik Nutrition & Care GmbH, Germany)

Maternal supplementation with Met led to greater (*P* value < 0.05) global DNA methylation compared with CON calves (Fig. 1). Among the proteins measured, the ratio of *p*-AKT:AKT (AKT Serine/Threonine Kinase) was greater in MET calves (*P* value < 0.001; Table 4). In contrast, MET calves had a lower ratio of p-S6K1:S6K1 (*P* value = 0.01; Table 4).

**Table 4:** Expression of mTOR pathway-related proteins in liver tissue from 4-d old Holstein calves (*n* = 6/group) born to cows randomly assigned to receive a basal control (CON) diet from − 28 ± 2 d to parturition [1.47 Mcal/kg dry matter (DM) and 15.3% crude protein (CP)] with no added Met or CON plus ethyl cellulose Met (MET, Mepron®, Evonik Nutrition & Care GmbH, Germany)

Protein (log-transformed data)	Maternal diet		SEM	***p***-value
	CON	MET		
*Total protein*				
mTOR	0.93	0.90	0.08	0.85
S6K1	2.55	2.38	0.10	0.43
AKT	2.49	1.48	0.19	<.00
4EBP1	2.39	2.42	0.17	0.93
eEF2	1.91	1.69	0.09	0.26
eIF2a	2.31	2.05	0.08	0.12
RPS6	2.07	2.33	0.21	0.57
*Phosphorylated protein*				
p-mTOR	0.62	0.69	0.06	0.59
p-S6K1	2.61	1.62	0.18	<.00
p-AKT	2.46	2.24	0.11	0.36
p-4EBP1	1.50	1.58	0.10	0.72
p-eEF2	0.98	0.59	0.16	0.25
p-eIF2a	2.41	2.08	0.15	0.29
p-RPS6	1.78	1.17	0.21	0.15
*Ratio*				
p-mTOR/mTOR	0.74	0.85	0.12	0.69
p-S6K1/S6K1	1.04	0.70	0.07	0.01
p-AKT/AKT	1.01	1.52	0.09	<.00
p-4EBP1/4EBP1	0.65	0.71	0.07	0.66
p-eEF2/eEF2	0.51	0.36	0.08	0.35
p-eIF2a/eIF2a	1.04	1.01	0.05	0.73
p-RPS6/RPS6	0.90	0.61	0.13	0.28

### Metabolomics, hepatic enzyme activity and mRNA abundance

At day 4, maternal supplementation with MET led to greater concentrations (P value ≤0.05) of Glycine, Adenosine, Serine, Taurine, Cystathionine, Glutamate, Fumarate, NAD, NADH, Taurocholic acid, Glycocholic acid, Lithocholic acid, and Glycochenodeoxycholic acid (Table 5). In contrast, lower hepatic activity for cystathionine β-synthase (CBS) and 5-methyltetrahydrofolate homocysteine methyltransferase (MTR) was detected in the MET calves (Table 6). Maternal supplementation with MET led to a lower abundance of *Phosphate Cytidylyltransferase 1, Choline, Beta* (*PCYT1B*; *P* value = 0.04), an overall tendency toward lower abundance of *Cysteine Dioxygenase Type 1* (*CDO1*) and greater abundance of *Phosphatidylethanolamine N-Methyltransferase* (*PEMT*) and *Methionine Adenosyltransferase 1A* (*MAT1A*) respectively (*P* value ≤0.10; Table 7).

**Table 5:** Metabolite concentrations (ng/mg of total protein) in liver tissue from 4-d old Holstein calves (*n* = 6/group) born to cows randomly assigned to receive a basal control (CON) diet from −28 ± 2 d to parturition [1.47 Mcal/kg dry matter (DM) and 15.3% crude protein (CP)] with no added Met or CON plus ethyl cellulose Met (MET, Mepron®, Evonik Nutrition & Care GmbH, Germany)

Metabolite, (ng/mg of total protein)	Maternal diet		SEM	***p***-value
	CON	MET		
***1-Carbon metabolism***				
Adenosine	3,855	8,411	1,052	0.02
Methionine	979.29	1,566	254.08	0.29
Betaine	15,282	21,058	3,424	0.44
Choline	3,165	4,550	500.53	0.19
Glycine	9,643	19,085	2,035	0.01
5-Methyltetrahydrofolic acid	8.06	12.08	2.67	0.49
N,N-Dimethylglycine	33.67	55.76	7.74	0.18
S 5′ Adenosyl homocysteine	320.39	426.08	55.01	0.37
S 5′ Adenosyl methionine	773.97	630.38	74.81	0.36
Butyrobetaine	69.95	112.76	14.61	0.17
***Transsulfuration***				
Cystathionine	346.41	738.41	95.37	0.04
Cysteine	1,438	2,898	437.86	0.12
Cysteinesulfinic acid	641.98	1,471	213.39	0.06
Glutamylcysteine	909.72	1,041	144.79	0.67
Glutathione	19,575	21,762	2,557	0.69
Hypotaurine	2,929	5,879	1,080	0.20
Serine	3,663	7,387	770.19	0.01
Taurine	56,308	117,719	13,488	0.03
***Tricarboxylic acid cycle***				
Ketoglutaric acid	468.75	541.95	57.50	0.55
Fumarate	1,775	3,504	419.50	0.04
Glutamate	33,518	65,748	7,593	0.04
NAD	10,074	23,436	2,829	0.01
FAD	2,563	4,017	464.71	0.14
NADH	3,796	7,733	994.65	0.05
Pyruvate	1,656	1,974	293.37	0.62
Malic acid	7,591	8,053	1,367	0.90
NAD/NADH	2.75	3.68	0.47	0.35
***Other***				
Glutamine	2,567	5,256	1,216	0.36
Carnitine	688.15	1170	160.62	0.16
***Bile acids***				
Glycocholic acid	6,444	10,845	1,054	0.05
Glycochenodeoxycholic acid	2,627	5,438	666.36	0.04
Taurocholic acid	1,829	3,106	271.17	0.02
Lithocholic acid	7,796	26,284	3,501	0.01
Taurochenodeoxycholic acid	1,738	3,010	320.82	0.06

**Table 6:** Hepatic activity of betaine homocysteine methyltransferase (BHMT), cystathionine β-synthase (CBS), and 5-methyltetrahydrofolate homocysteine methyltransferase (MTR) in liver tissue from 4-d old Holstein calves (*n* = 6/group) born to cows randomly assigned to receive a basal control (CON) diet from − 28 ± 2 d to parturition [1.47 Mcal/kg dry matter (DM) and 15.3% crude protein (CP)] with no added Met or CON plus ethyl cellulose Met (MET, Mepron®, Evonik Nutrition & Care GmbH, Germany)

Enzyme (nmol product. h^**−1**^. mg protein^**−1**^)	Maternal diet		SEM	***p***-value
	CON	MET		
CBS	16.40	4.43	2.16	<.00
MTR	17.69	5.48	2.28	0.01
BHMT	20.62	14.88	2.39	0.26

**Table 7:** Abundance of genes related to methionine metabolism, DNA methylation, glutathione metabolism, and cytidine 5′-diphosphocholine (CDP)–choline pathway in liver tissue from 4-d old Holstein calves (n = 6/group) born to cows randomly assigned to receive a basal control (CON) diet from − 28 ± 2 d to parturition [1.47 Mcal/kg dry matter (DM) and 15.3% crude protein (CP)] with no added Met or CON plus ethyl cellulose Met (MET, Mepron®, Evonik Nutrition & Care GmbH, Germany)

Gene (log_**2**_ scale)	Maternal diet		SEM	***p***-value
	CON	MET		
***Methionine cycle***				
*MAT1A*	0.01	0.03	0.01	0.09
*BHMT*	1.23	0.98	0.11	0.26
*BHMT2*	0.06	0.11	0.04	0.33
*MTR*	0.73	1.04	0.11	0.19
*SAHH*	0.04	0.10	0.01	0.53
*PEMT*	0.09	0.17	0.02	0.07
*BADH*	1.16	1.20	0.06	0.73
*CHDH*	1.57	1.50	0.15	0.76
*SARDH*	0.93	0.83	0.06	0.40
***DNA methylation***				
*DNMT1*	0.95	0.94	0.03	0.93
*DNMT3A*	0.97	0.88	0.05	0.36
***Transsulfuration***				
*CBS*	0.76	0.73	0.05	0.81
*CDO1*	0.91	0.74	0.05	0.08
*CSAD*	0.34	0.30	0.02	0.37
*CTH*	1.18	1.45	0.09	0.20
***Glutathione pathway***				
*GSS*	1.02	0.97	0.03	0.48
*GCLC*	0.50	0.52	0.06	0.95
*GSR*	0.61	0.70	0.04	0.27
*GPX1*	0.86	1.02	0.06	0.13
***CDP–choline pathway***				
*CHKA*	0.75	0.96	0.10	0.31
*CHKB*	0.76	0.76	0.03	0.94
*PCYT1A*	0.94	0.98	0.05	0.66
*PCYT1B*	0.82	0.50	0.08	0.04
*CEPT1*	0.94	0.96	0.05	0.92

### RNA sequencing and gene expression analyses

**Fig. 2:**
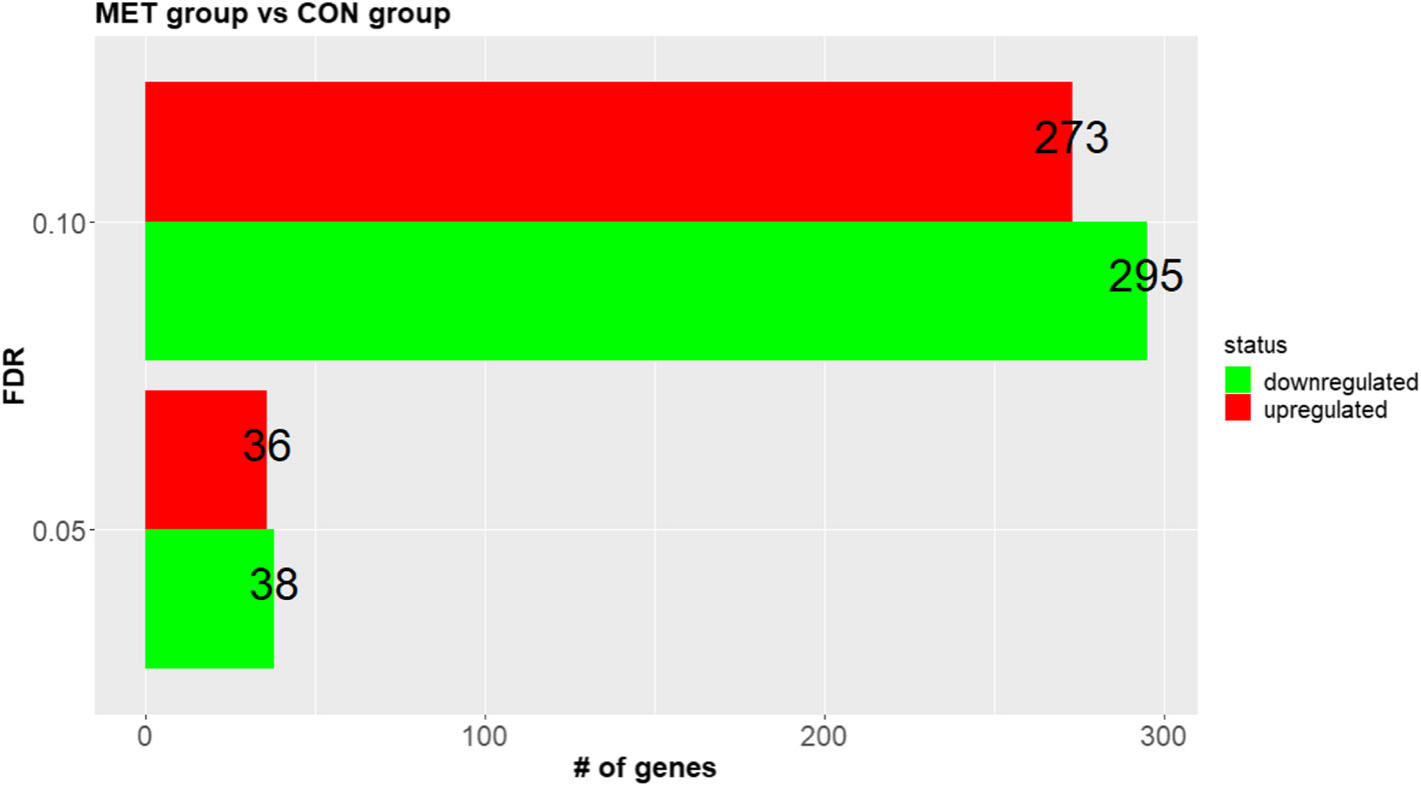
Differentially expressed genes (DEG; at FDR ≤0.10 and ≤ 0.05) from RNAseq data of liver tissue from 4-d old Holstein calves (*n* = 6/group) born to cows randomly assigned to receive a basal control (CON) diet from − 28 ± 2 d to parturition [1.47 Mcal/kg dry matter (DM) and 15.3% crude protein (CP)] with no added Met or CON plus ethyl cellulose Met (MET, Mepron®, Evonik Nutrition & Care GmbH, Germany). The green bars indicate downregulation, while the red bars indicate upregulation. The set of bars indicates differentially expressed genes at 0.05 and 0.10 FDR cut-offs considering in both cases the comparison of MET vs. CON

A summary of sequencing read alignment and mapping is reported in Additional File 1. Overall, samples had approximately 12 million reads of which 11 million (~ 95%) were uniquely mapped and 9.4 million (~ 78%) assigned to genes. Statistical analysis identified 13,867 uniquely annotated (EntrezID) genes. Of these, applying the 0.05 FDR cut-off, 74 genes (36 upregulated and 38 downregulated) were detected as differentially expressed (DEG) comparing MET with CON heifer calves, whereas 568 DEG (273 upregulated and 295 downregulated) were detected at FDR ≤ 0.10 cut-off (Fig. 2). A summary of the top-ten up- and downregulated genes (FDR ≤ 0.10) is reported in Tables 8 and 9. The entire list of DEG is reported in Additional File 2.

**Table 8:** Top 20 upregulated genes among those that were differentially expressed (DEG; FDR ≤0.10) in RNAseq data from liver tissue of 4-d old Holstein calves (n = 6/group) born to cows randomly assigned to receive a basal control (CON) diet from −28 ± 2 d to parturition [1.47 Mcal/kg dry matter (DM) and 15.3% crude protein (CP)] with no added Met or CON plus ethyl cellulose Met (MET, Mepron®, Evonik Nutrition & Care GmbH, Germany)

Entrez gene id	Gene Symbol	FC	FDR
618,238	*M-SAA3.2*	5.70	0.08
520,625	*SLC44A4*	4.46	0.07
516,626	*SLC51A*	4.06	0.09
516,291	*REC8*	3.88	0.07
782,922	*LOC782922*	3.72	0.10
281,644	*BFSP1*	3.42	0.04
530,071	*CRIP3*	2.97	0.09
505,642	*GSTO1*	2.85	0.08
514,770	*NR0B2*	2.64	0.05
527,458	*KLHL26*	2.58	0.08
504,950	*NPM3*	2.58	0.05
523,874	*FAM184B*	2.38	0.10
613,523	*EAF2*	2.32	0.08
514,979	*CLEC6A*	2.30	0.08
506,900	*HIST1H2AC*	2.27	0.10
536,607	*ACTN2*	2.21	0.09
104,968,464	*RPP21*	2.09	0.04
509,463	*ATCAY*	2.07	0.09
506,043	*FKBP11*	2.04	0.09
100,848,337	*BHLHA15*	2.02	0.07

**Table 9:** Top 20 downregulated genes among those that were differentially expressed (DEG; FDR ≤0.10) in RNAseq data from liver tissue of 4-d old Holstein calves (*n* = 6/group) born to cows randomly assigned to receive a basal control (CON) diet from −28 ± 2 d to parturition [1.47 Mcal/kg dry matter (DM) and 15.3% crude protein (CP)] with no added Met or CON plus ethyl cellulose Met (MET, Mepron®, Evonik Nutrition & Care GmbH, Germany)

Entrez gene id	Gene Symbol	FC	FDR
527,436	*MSI1*	−4.89	0.07
538,404	*MYOM1*	−4.08	0.07
521,822	*FADS2*	−4.02	0.08
526,134	*SLC9A2*	−3.90	0.09
533,161	*KIF2C*	−3.78	0.08
518,623	*LOC518623*	−3.60	0.08
327,679	*CCNB1*	−3.59	0.07
518,801	*CFAP43*	−3.40	0.05
528,813	*SCNN1D*	−3.23	0.08
537,698	*GRIK5*	−3.22	0.06
281,061	*CDK1*	−3.15	0.08
281,883	*KCNJ2*	−3.12	0.07
534,781	*PBK*	−3.06	0.09
504,746	*ECT2*	−2.82	0.09
616,767	*PRRG4*	−2.81	0.10
518,313	*KIAA1324L*	−2.77	0.08
538,436	*CCNE2*	−2.72	0.04
785,540	*LOC785540*	−2.65	0.09
517,383	*GPRIN1*	−2.42	0.08
615,890	*SMG8*	−2.40	0.08

### KEGG pathway analysis

**Fig. 3:**
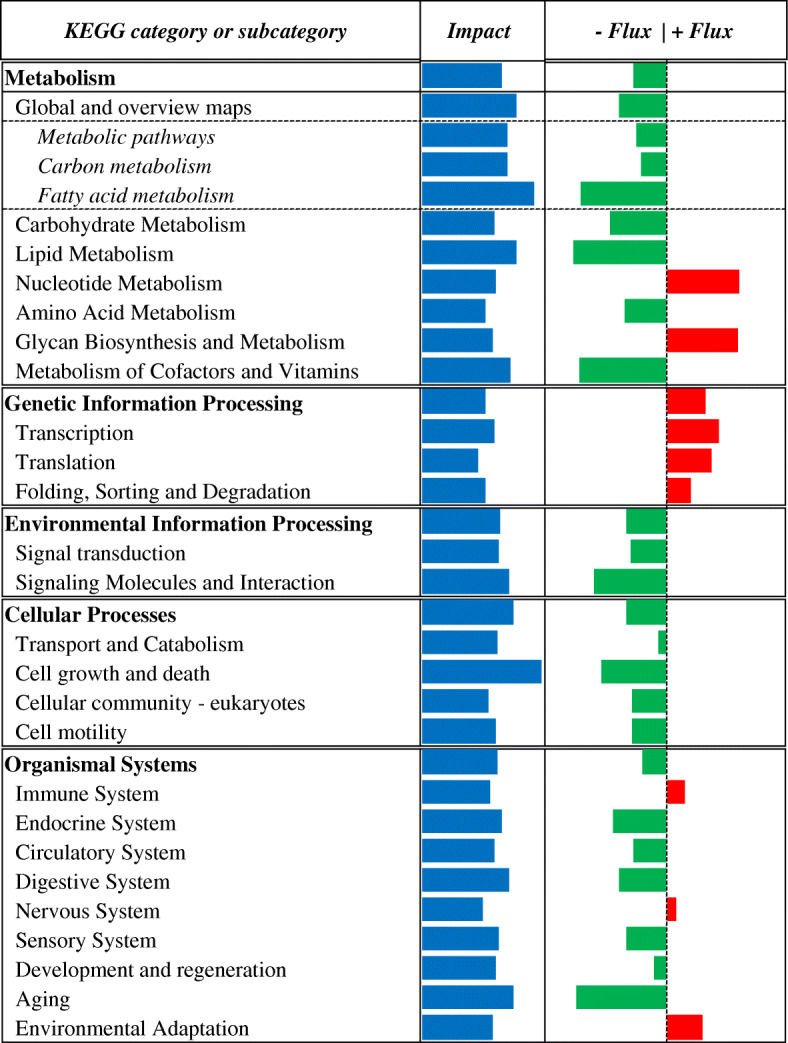
Summary of KEGG metabolic subcategories resulting from the DIA analysis of liver tissue transcriptome from 4-d old Holstein calves (n = 6/group) born to cows randomly assigned to receive a basal control (CON) diet from − 28 ± 2 d to parturition [1.47 Mcal/kg dry matter (DM) and 15.3% crude protein (CP)] with no added Met or CON plus ethyl cellulose Met (MET, Mepron®, Evonik Nutrition & Care GmbH, Germany). The columns represent the effect (impact) and flux responses. The blue bars represent the effect value (0 to 300), and the flux columns represent negative (−) and positive (+) flux (− 300 to + 300) based on the direction of the effect. The positive flux (red bars) indicates an upregulation in treated (MET) liver tissue cells compared to control (CON) ones, while the negative flux (green bars) indicates a downregulation

**Fig. 4:**
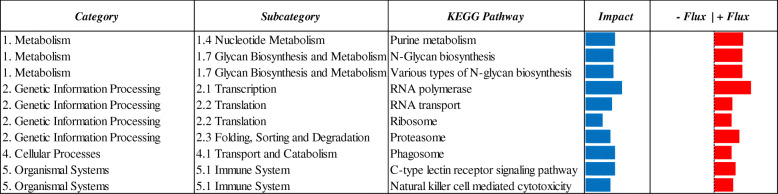
Summary of top-10 upregulated KEGG pathways resulting from the DIA analysis of liver tissue transcriptome from 4-d old Holstein calves (*n* = 6/group) born to cows randomly assigned to receive a basal control (CON) diet from − 28 ± 2 d to parturition [1.47 Mcal/kg dry matter (DM) and 15.3% crude protein (CP)] with no added Met or CON plus ethyl cellulose Met (MET, Mepron®, Evonik Nutrition & Care GmbH, Germany). The columns represent the effect (impact) and flux responses. The blue bars represent the effect value (0 to 300), and the flux columns represent negative (−) and positive (+) flux (− 300 to + 300) based on the direction of the effect. The positive flux (red bars) indicates an upregulation in treated (MET) liver tissue cells compared to control (CON) ones, while the negative flux (green bars) indicates a downregulation

**Fig. 5:**
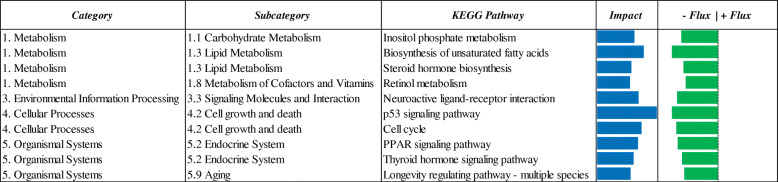
Summary of top-10 downregulated KEGG pathways resulting from the DIA analysis of liver tissue transcriptome from 4-d old Holstein calves (n = 6/group) born to cows randomly assigned to receive a basal control (CON) diet from − 28 ± 2 d to parturition [1.47 Mcal/kg dry matter (DM) and 15.3% crude protein (CP)] with no added Met or CON plus ethyl cellulose Met (MET, Mepron®, Evonik Nutrition & Care GmbH, Germany). The columns represent the effect (impact) and flux responses. The blue bars represent the effect value (0 to 300), and the flux columns represent negative (−) and positive (+) flux (− 300 to + 300) based on the direction of the effect. The positive flux (red bars) indicates an upregulation in treated (MET) liver tissue cells compared to control (CON) ones, while the negative flux (green bars) indicates a downregulation

The Dynamic Impact Approach (DIA) analysis yields the impact and flux of all the manually-curated pathways included in the KEGG database. The term ‘impact’ refers to the biological importance of a given pathway as a function of the change in expression of genes composing the pathway (proportion of DEG and their magnitude) in response to a treatment, condition, or change in physiological state. Consequently, the direction of the impact, or flux, characterizes the average change in expression as up-regulation/activation, down-regulation/inhibition, or no change. Considering DIA results with DEG at FDR ≤ 0.10, a broad effect on the transcriptome due to maternal MET was detected (Fig. 3). All main KEGG categories, both metabolic (‘Metabolism’) and non-metabolic (‘Environmental information processing’, ‘Cellular processes’, and ‘Organismal systems’) were broadly impacted with a negative flux (i.e. downregulated), except for ‘Genetic information processing’ that was markedly upregulated (Fig. 3). In particular, ‘Cell growth and death’, ‘Lipid Metabolism’, ‘Aging’, ‘Metabolism of Cofactors and Vitamins’ and ‘Signaling Molecules and Interaction’ were the most-impacted and downregulated KEGG subcategories (Fig. 5). Along with ‘Genetic Information Processing’ pathways, the ‘Nucleotide Metabolism’, ‘Glycan Biosynthesis and Metabolism’, ‘Environmental adaptation’, ‘Immune System’ and ‘Nervous system’ were the most-impacted subcategories with a positive flux (i.e. upregulation) in MET vs. CON comparison (Fig. 3). Top-10 up- and downregulated single pathways are reported in Figs. 4 and 5, respectively.

### Transcription regulator discovery

The transcription factor enrichment analysis with the ChIP-X Enrichment Analysis 3 (ChEA3) tool generated a list of 72 TF significantly-associated (FDR ≤ 0.05) with our DEG at the FDR ≤ 0.10 threshold. The list of top 10-ranked upstream regulators is reported in Table 10, whereas the entire list of TF is reported in Additional File 3. Applying the DIA approach to the ChEA3 results, the TF impact and flux values were predicted (Table 10).

**Table 10:** Summary of the top 10-ranked upstream regulators (TF) out of the 72 identified using the ChEA3 tool and significantly associated (FDR cutoff ≤0.05) with our differentially expressed gene (DEG) list obtained in RNAseq data from liver tissue of 4-d old Holstein calves (*n* = 6/group) born to cows randomly assigned to receive a basal control (CON) diet from − 28 ± 2 d to parturition [1.47 Mcal/kg dry matter (DM) and 15.3% crude protein (CP)] with no added Met or CON plus ethyl cellulose Met (MET, Mepron®, Evonik Nutrition & Care GmbH, Germany)

TF	Impact	Flux	Predicted State	Rank	P value	FDR	Overlapping DEG
MYC	178.42	57.40	Activated	1	4.11E-09	1.26E-06	*POP1, GMFG, SMC4, KRTCAP2, CDK5RAP3, IER2, CLPP, DDIT3, UBA52, RPL28, NPRL2, ABCB8, ANAPC11, BLOC1S6, PRKAR2A, TOE1, NR1D2, NFATC4, MRPL38, ABCC10, CYB561D2, MCEE, GSTO1, SUMF1, RNF167, TUT1, SF3B5, RIDA, PSMB2, BID, SF3A2, TARBP2, LAMTOR2, HSPA1A, STK16, MAP 2 K3, CNPY3, LENG1, SNRPA, ENY2, INO80E, GOT1, DHCR24, FOXN2, G6PC3, TMEM11, RAD9A, BMPR2, ZC3H3, MRPL4, NGRN, GPX1, REXO4, EVC, SNRPA1, ZBTB44, MRM1, NCLN, POLR2I, POLR2J, ATOX1, KLF5, KLF9, POLR3H, EIF4A2, SMARCB1, NUBP1, NUBP2, SNRPD1, RPL36AL, PDK1, ALG5, MRPS18B, MRPS18A, ALG3, IMP4, SNX4, PTDSS2, VAT1, ALDH6A1, CALM3, SUGP1, FKBP11, SLC44A4, GRIK5, GUK1, SEC61B, PRMT6, TMED9, NME1, APRT, RPUSD4, KIF2C, YAP1, NEDD8, NT5C, PES1, NPM3, GEMIN2, NR3C1, CHCHD1, ZGPAT, DPM2, DOK2, CCNE2, CCNB1, SHARPIN, DHRS7B, RHBDD3, NONO, DLG1, NABP2, COPE, CHD9, DDX41, YWHAG, PEX1, EEF1D, DOCK1, PFDN6, PNKP, PSMB10, TBRG4, PRRG4, RMND5A, MAPK6, SLC50A1, WEE1*
E2F1	178.13	31.67	Activated	2	7.28E-08	7.45E-06	*SMC4, SMC2, MYC, MPV17, CDK5RAP3, IER2, VPS18, AARSD1, RNF125, CLPP, DDIT3, RPL28, RPN1, ABCB8, ANAPC11, BLOC1S6, MCTS1, TOE1, MCAT, VANGL1, ADI1, MMS19, MRPL38, ING1, NUDC, ANXA4, SUMF1, CDC42EP5, TUT1, SF3B5, AHNAK, RNF138, BID, SF3A2, TARBP2, DCBLD2, SNX15, ATCAY, STK16, CNPY2, LENG1, UTRN, ENY2, FECH, FOXO4, ATXN7, AMDHD2, EIF2B4, GOT1, DHCR24, FOXN2, TMEM11, RAD9A, MRPL4, NGRN, GPX1, REXO4, COASY, SNRPA1, TSSC4, GTF3C5, HM13, MRM1, SH3BP2, POLR2G, FNIP1, KCNJ2, FOXJ3, GFER, ATOX1, KLF5, KLF9, SLC25A30, POLR3H, EIF4A2, SMARCB1, NUBP1, NUBP2, SNRPD1, RPL36AL, EIF2D, TNS3, PDK1, CPT1A, ACTN2, MRPS18B, MRPS18A, ALG3, IMP4, PLPP3, PTDSS2, RACGAP1, VAT1, EAF2, FKBP11, INTS11, MRPS11, CITED4, ROBO1, ACADM, PRMT6, ZFX, ZDHHC17, NME1, APRT, KIF2C, YAP1, UQCR11, TYMS, NT5C, PES1, SCAND1, YIPF1, ITCH, GEMIN2, CHCHD1, HIST1H2AC, ZGPAT, ACYP2, ALDH3A2, DPM2, CCNE2, RPP21, COX19, DPP4, SHARPIN, DHRS7B, ECT2, KLHL26, NONO, HSPA2, TJAP1, BRWD1, PICALM, DDX41, ADGRE5, ARHGEF10, TSFM, PEX1, ETV5, TRAPPC6A, EEF1D, CAT, PFDN6, PNKP, PPP2R3C, TAF10, WEE1, CDK2AP2*
CREB1	174.61	24.88	Activated	3	2.87E-07	2.20E-05	*KRTCAP2, IER2, CHID1, VPS18, BIN3, BIN1, DDIT3, FDX2, PRKD2, UBA52, EXD2, CDCA2, ANAPC11, BLOC1S6, MCTS1, IRF2BP2, TYSND1, NR1D2, FOSL2, MMS19, ING1, CYB561D2, EMD, TUT1, PPARA, SF3B5, PSMB2, TARBP2, SNX15, LAMTOR2, CHPF, LTN1, GOLIM4, FADS2, STK16, MAP 2 K3, CNPY3, SNRPA, ENY2, RLF, EIF2B4, GOT1, TMEM11, CPD, ZC3H3, MRPL4, DAD1, TSSC4, HM13, CXXC1, NCLN, SEC11C, SPAG9, OXR1, KLF6, KLF5, KLF9, TRMT61A, EIF4A2, PANK3, JMJD1C, TXNDC16, NUBP2, EFR3A, GOLPH3L, PREB, APOB, ZRANB1, EAF2, NDUFAF3, CALM3, SUGP1, GUK1, TMED9, KIF2C, AMIGO1, RTL6, CHCHD1, ALG12, BRMS1, SHARPIN, ANKZF1, CBX8, RHBDD3, DENND4A, NONO, NABP2, GMPPA, YWHAG, YWHAZ, ETV5, EEF1D, HLF, NR2C2, PSMB10, MAPK6, TAF10, WEE1, CDK2AP2, RBM42*
PPARG	178.93	−68.11	Inhibited	4	5.64E-07	3.46E-05	*ZC3H12D, SCP2, MYC, NAMPT, WDR91, IER2, CHID1, AARSD1, HDHD5, RNF125, BIN3, CLPP, DDIT3, PRKD2, RPL28, RPN1, MRPL16, PRKAR2A, IRF2BP2, RMDN2, FOSL2, FERMT1, PAH, MTMR10, EEA1, GSTO1, ANXA5, SUMF1, KAT2B, HEATR5A, AEBP2, TUT1, PLCB1, AHNAK, BID, RNLS, BBOX1, TARBP2, LAMTOR5, UHRF1BP1L, MYLK4, FADS2, CDH2, ZBTB39, UTRN, ENY2, AMDHD2, GOT1, FOXN2, CDK1, MDM2, SERINC5, NGRN, GPX1, PIK3CA, MET, GTF3C5, ROCK2, GLRX, MRM1, FNIP1, POLR2J, CYB5A, FOXJ3, DDHD2, PTPN12, CDC42BPA, KBTBD11, BICC1, KLF6, NAGK, KLF9, SLC25A30, PANK3, SMARCB1, TNS3, CPT1A, ECH1, MRPS18B, MRPS18A, PREB, PLPP3, CFTR, STK38L, PCK1, ZRANB1, MYO5C, CUTC, IDE, ROBO1, ACADM, ZDHHC17, RPUSD4, CD47, C1QB, SH3BGRL, KLB, GEMIN2, SSC4D, SELENOP, COX19, COBLL1, ACBD5, CRIM1, HOOK3, ARHGAP25, TJAP1, DLG1, GCLM, PICALM, CHD9, TNFAIP3, PPP1R9B, ADGRE5, YWHAG, ARHGEF10, PEX1, YWHAZ, ACOX1, CAT, KDM7A, USP12, NR2C2, PSMB10, RMND5A, TNKS2, MAPK6, TMEM30B, CMTM4*
KLF1	165.60	73.79	Activated	6	9.92E-07	4.19E-05	*SPI1, KRTCAP2, MYC, AARSD1, CLPP, RPN1, TYSND1, VANGL1, ADI1, MRPL38, EMD, GSTO1, TUT1, SNX15, SMCHD1, SNRPA, FECH, CORO1A, EIF2B4, GOT1, DHCR24, COPS9, SORD, SNRPA1, HM13, CXXC1, NCLN, SEC11C, NUBP2, SNRPD1, SOX6, PDK1, ECH1, IMP4, PREB, SYMPK, CALM3, IDE, RAC2, TMED9, UQCR11, NEDD8, NT5C, CHCHD1, HIST1H2AC, CYBA, BRMS1, RPP21, PPIH, RPS19BP1, ACBD5, NABP2, GCLM, PPP1R9B, YWHAG, TRAPPC6A, CAT, PFDN6, TBRG4, TAF10*
SPI1	173.45	5.80	Activated	5	9.49E-07	4.19E-05	*SPI1, GMFG, SLC4A4, SCP2, PSMD4, NAMPT, CDK5RAP3, IER2, RNF125, PRKD2, RPL28, EXD2, CFP, BLOC1S6, WDR70, MCTS1, NR1D2, NFATC4, FERMT1, PYGL, ING1, PQBP1, EEA1, TRMT112, MRPL42, GSTO1, ANXA4, GMIP, SUMF1, RNF167, TUT1, PPARA, FAM214A, APOLD1, BID, PET100, LAPTM5, PSMB8, LAMTOR2, GOLIM4, MYLK4, PYCARD, ATCAY, STK16, MAP 2 K3, TPM1, CNPY3, CLIP1, CORO1A, YBEY, AMDHD2, MAP 3 K20, GOT1, CYBRD1, DHCR24, FOXN2, BFSP1, G6PC3, CPD, CCDC22, SERINC5, NGRN, PSTPIP1, SORD, DAD1, ROCK2, UNC93B1, CXXC1, GLRX, HSD17B11, HECTD1, SH3BP2, POLR2G, PTPN18, PTPN12, OXR1, FBXL12, SLC25A30, PANK3, JMJD1C, MRPS18B, MAP 7, STK38L, ASL, CD14, STK11IP, STAG2, NDUFAF3, RAC2, TMED9, ZFX, ZDHHC17, VAV1, C1QB, TOR4A, AIF1, PES1, C1QC, WASHC4, NR3C1, EME2, KNTC1, ZGPAT, ACYP2, CYBA, TYROBP, RPS19BP1, CCNB1, CBX8, ACBD5, HOOK3, ARHGAP25, BRWD1, PICALM, CHD9, PPP1R9B, GMPPA, CNPPD1, YWHAZ, ETV5, CAT, PNKP, USP12, TBRG4, STOM, TAF10, SLC50A1, BUD13*
ZFX	170.08	15.72	Activated	7	1.23E-06	4.19E-05	*NCKAP1, PSMD4, NAMPT, IER2, AARSD1, HDHD5, CLPP, UBA52, RPL28, RPN1, ABCB8, ANAPC11, PRKAR2A, TTPA, TOE1, MCAT, NR0B2, VANGL1, ADI1, MRPL38, EEA1, EMD, NUDC, GSTO1, SUMF1, AHNAK, PSMB2, RNF138, SC5D, BID, SF3A2, TARBP2, DCBLD2, STK16, ZBTB39, CNPY2, CNPY3, LENG1, CLIP1, FKBP9, SNRPA, ENY2, FECH, ATXN7, NEK9, FOXN2, BFSP1, G6PC3, MDM2, RAD9A, ISY1, MRPL4, REXO4, COASY, EVC, SNRPA1, GTF3C5, ZBTB44, FNIP1, FOXJ3, ATOX1, SLC25A30, POLR3H, EIF4A2, PANK3, SMARCB1, SNRPD1, FBXO3, TNS3, PDK1, MRPS18A, NEIL2, FNDC4, SNX4, PTDSS2, STK38L, RAC2, PRMT6, ZFX, ZDHHC17, VAV1, AMIGO1, PES1, SCAND1, ITCH, GEMIN2, RTL6, ZGPAT, CYBA, ALDH3A2, DPM2, CCNE2, RHBDD3, KLHL26, NONO, TJAP1, NABP2, GCLM, BRWD1, CHD9, ADGRE5, ETV5, TRAPPC6A, LATS2, ACOX1, EEF1D, ZMYND11, CLN5, TBRG4, MARVELD2, MAPK6, TMEM30B, CMTM4, WEE1, CDK2AP2, BUD13*
CEBPB	167.51	36.64	Activated	8	1.79E-06	5.00E-05	*MYC, CHID1, AARSD1, BIN3, RUFY3, FDX2, CFP, NR2C2AP, TOE1, TYSND1, RMDN2, MCAT, ABCC10, CYB561D2, EMD, PCMTD1, GMIP, RNF167, PLCB1, PDF, PSMB8, LAMTOR5, CHPF, MPV17L2, STK16, ZBTB39, CNPY2, INSIG2, FBXO48, FOXO4, RLF, IP6K2, BCL3, RAD9A, COPS9, NGRN, CXXC1, MRM1, POLR2G, FOXJ3, PTPN12, OXR1, GFER, KBTBD11, FBXL12, KLF6, SLC25A30, NUBP2, RPL36AL, ECH1, IMP4, PREB, SNX4, STK38L, B4GALNT1, ZRANB1, FKBP11, IDE, GUK1, STBD1, SEC61B, PRMT6, TMED9, CYBA, CBX8, RHBDD3, PICALM, ARHGEF10, TSFM, EEF1D, NR2C2, MAPK6, LUM, TAF10, SLC50A1, MAPK12, CDK2AP2, BUD13*
HNF4A	175.29	−51.68	Inhibited	9	3.65E-06	9.34E-05	*NCKAP1, ZC3H12D, KRTCAP2, SCP2, PSMD4, MYC, RUFY3, CDCA2, NPRL2, MRPL16, WDR70, PRKAR2A, TTPA, IRF2BP2, TYSND1, NR0B2, FOSL2, FERMT1, ADI1, PAH, MMS19, HRG, MYOM1, MTMR10, PYGL, ABCC10, EEA1, MRPL42, NUDC, GSTO1, ANXA4, ANXA5, SUMF1, HEATR5A, TUT1, PPARA, PLCB1, RAPGEF5, SF3B5, AHNAK, PDF, CHN2, BID, KIDINS220, BBOX1, DCBLD2, GATM, AADAC, SLMAP, SNX15, LAMTOR5, UHRF1BP1L, CHPF, GOLIM4, PLOD2, IL1RAP, MYLK4, PYCARD, CDH2, MAP 2 K3, CNPY3, CLIP1, UTRN, CALCRL, INSIG2, AMDHD1, ATXN7, AMDHD2, ANKRD29, EIF2B4, GOT1, DHCR24, FOXN2, TMEM11, CPD, ALB, SLCO1A2, BCL3, SERINC5, ZNF771, ISY1, ZC3H3, MRPL4, PSTPIP1, REXO4, COASY, EVC, PCCA, DAD1, MET, TSSC4, GTF3C5, ROCK2, UNC93B1, HM13, GLRX, HSD17B11, HECTD1, NCLN, SH3BP2, FNIP1, POLR2I, SEC11C, SPAG9, CYB5A, METAP1D, SLC35A3, FOXJ3, PTPN12, CDC42BPA, OXR1, BICC1, ATOX1, FBXL12, KLF6, POLR3H, EIF4A2, JMJD1C, TXNDC16, SOX6, TNS3, CPT1A, ACTN2, GOLPH3L, MRPS18A, PLPP3, SNX4, RACGAP1, HLTF, STK38L, SYMPK, ASL, STXBP5, CD14, PCK1, APOB, STK11IP, ALDH6A1, STAG2, MYO5C, CALM3, CUTC, SUGP1, ROBO2, ROBO1, SH3PXD2B, ACADM, TMED9, ZFX, NME1, MICU3, YAP1, UQCR11, NEDD8, AIF1, PES1, SCAND1, IL33, YIPF1, ITCH, KLB, SSC4D, NR3C1, SRP14, SBF2, ZGPAT, IL11RA, LMO7, ACYP2, ALDH3A2, BRMS1, SELENOP, PPIH, COX19, DPP4, SHARPIN, COBLL1, DHRS7B, ACBD5, TCAF1, ARHGEF37, DENND4A, NONO, CRIM1, HOOK3, TJAP1, DLG1, DLG5, COPE, PICALM, CHD9, DOCK7, TNFAIP3, GMPPA, YWHAG, PEX1, YWHAZ, LATS2, RABEP2, ACOX1, ALDH1A1, CAT, DOCK1, HLF, PSMB10, PRRG4, TNKS2, MARVELD2, PPP2R3C, STOM, CMTM4, ATP2B1, WEE1, RBM42, SNTB1*
FOXO1	181.39	−36.48	Inhibited	10	7.35E-06	1.73E-04	*GMFG, ZC3H12D, IER2, VPS18, HDHD5, RNF125, BIN1, UBA52, RPN1, MCTS1, IRF2BP2, NR1D2, FOSL2, MMS19, MTMR10, PYGL, ING1, PCMTD1, AEBP2, AHNAK, LAPTM5, LAMTOR5, FADS2, SMCHD1, TPM1, UTRN, IP6K2, GOT1, RAD9A, ISY1, PTPN21, PSTPIP1, DAD1, HM13, GLRX, MRM1, HECTD1, PTPN18, FOXJ3, KBTBD11, KLF6, NAGK, TRMT61A, TXNDC16, PDK1, MRPS18A, PLPP3, NEIL1, ZRANB1, STK11IP, STAG2, GUK1, SH3PXD2B, RAC2, ACADM, ZFX, CD47, PTGER3, SH3BGRL, SSC4D, NR3C1, SRP14, GPRIN3, TYROBP, CCNE2, RPP21, COBLL1, ECT2, ACBD5, DENND4A, ARHGAP25, GCLM, PICALM, TNFAIP3, PPP1R9B, ADGRE5, USP12, MAPK6, SNTB1*

## Discussion

The present findings were broadly consistent with our previous reports investigating the effect of feeding MET during late-pregnancy on cow and calf hepatic function [10, 24, 26, 27, 30, 31, 34, 35]. Briefly, in those previous studies enhanced MET supply improved immunometabolism along with DMI in dairy cows during the peripartal period and through peak lactation [24, 34, 35]. In the larger cohort of calves from this study encompassing birth through the first 9 wk. of life, we reported that maternal MET supply influenced enzyme activity and metabolome in one-carbon metabolism and the tricarboxylic acid (TCA) cycle, with beneficial physiological advantages to calves [26]. Furthermore, previous results on hepatic target-gene transcription have suggested that feeding Met during late-pregnancy was associated with faster maturation of key metabolic pathways involved in the calf’s ability to quickly adapt to extrauterine life [10, 30]. Thus, RNA-seq results alone revealed that feeding Met during late-gestation broadly altered neonatal calf liver transcriptome profiles. In particular, transcriptome profiles confirmed the hypothesis of an enhanced immunometabolic status attributable to the change in expression profiles of several genes mainly involved in ‘Glucose metabolism’, ‘Lipid metabolism, ‘Glutathione’, and ‘Immune System’ metabolism.

Although the focus of the present integrative analyses only encompassed day 4 of age, together, the data supported the idea that feeding Met to enhance post-ruminal supply in the cow during late-gestation primed or programmed the Met cycle in calf liver, hence, contributing to better rates of growth and development [26]. For instance, the greater concentrations of adenosine and serine observed in MET calves at this early age support the hypothesis of a priming effect of the Met cycle. The conversion of Met to SAM is accompanied by ATP consumption in a reaction controlled by Met adenosyltransferase (MAT) [36] and after demethylation, SAM is converted to SAH, which is subsequently hydrolyzed to homocysteine with the production of adenosine [37]. The tendency for greater *MAT1A* abundance in MET calves (*P* value < 0.10; Table 7) supported this idea. In addition, the greater serine concentrations observed in MET calves supported this hypothesis, since it is known that serine supports the Met cycle by providing one-carbon units to regenerate Met from homocysteine and through de novo ATP synthesis [38]. In this context, the downregulation of *Thymidylate synthase* (*TYMS*) [FC = − 1.50] was also noteworthy. TYMS converts deoxyuridine monophosphate (dUMP) to deoxythymidine monophosphate (dTMP) in a 5,10-methylene-tetrahydrofolate (THF)-dependent reaction [39]. Its downregulation indirectly suggested a greater concentration of the most reduced form of folate 1C unit, 5-methyl-THF that has a unique cellular fate, the remethylation of homocysteine to form methionine [39]. In light of current and previous observations, we speculate that despite the lower activity of MTR in liver from MET calves, enzymatic efficiency might have been greater. More in-depth discussion on this point is available in Additional File 4.

The greater concentration of serine also indicated the potential for greater metabolic activity through the transsulfuration pathway in MET calves, an idea supported by the greater concentration of a number of metabolites including cystathionine and taurine. The former is the product of the CBS reaction using homocysteine and serine, which is considered rate-limiting in the transsulfuration pathway [9]. Thus, despite the lower CBS activity, based on the greater concentrations of a number of intermediate metabolites (e.g. cystathionine, cysteinesulfinic acid), we speculated that efficiency of the CBS reaction was such that flux through the transsulfuration pathway was overall greater in MET calves. As such, concentrations of the cellular antioxidant GSH [40] also increased, and could have elicited beneficial effects on antioxidant responses in the calves.

A greater degree of Met metabolism was also indirectly suggested in our experiment by the marked upregulation of the nucleotide metabolism pathway (Fig. 4), since the role played by Met in Purine synthesis is well-documented [41]. Considering that Met plays an essential role in epigenetics via DNA methylation [42] and that it has been demonstrated that maternal methyl donor supplementation during pregnancy can regulate epigenetics via DNA methylation [43], the overall upregulation of ‘Genetic information processing’ pathways was compatible with the greater global DNA methylation detected in MET calves (Fig. 1). The evident upregulation of *TRMT61A* (*tRNA Methyltransferase 61A*) [FC = 1.69] was consistent with this scenario, as well as the upregulation of *POLR3H* (*RNA Polymerase III Subunit H*) [FC = 1.72] and *POLR2I* (*RNA Polymerase II Subunit I*) [FC = 1.71] (Additional File 2). In this context, the marked upregulation of histone *HIST1H2AC* [FC = 2.27] was noteworthy considering that SAM is also the most-important methyl-donor for histone methyltransferases [44]. Recent studies demonstrated how increased SAM and histone methylation levels are associated with genetic ablation of MAT in *Saccharomyces pombe* [45]. In the general context of nutritional programming, the marked upregulation of *Meiotic recombination REC8* [FC = 3.88] and downregulation of *cyclin B1* (*CCNB1*) [FC = − 3.59] and *cyclin-dependent kinase 1* (*CDK1*) [FC = − 3.15] was intriguing. These changes could potentially be biologically-relevant considering the role of these genes in oocyte meiosis [46, 47] and that methionine adenosyltransferase 2B (MAT2B) influences oocyte maturation in mouse by regulating the MAPK pathway [48]. Further studies will have to be performed to ascertain the mechanistic relevance of these changes in the context of hepatic nutritional programming of the calf.

### Glucose metabolism

Studies in humans and mice suggest that DNA methylation plays a crucial role in tissue-specific insulin-induced gene expression [49]. Both in vivo and in vitro studies confirmed that AKT is an indicator of hepatic and adipose insulin sensitivity in dairy cows [50, 51]. Thus, the greater phosphorylated AKT ratio in MET calves suggested they were more sensitive to insulin, which is consistent with the tendency for lower plasma glucose concentrations in those calves (*P* value = 0.08; Table 2) despite the lack of statistical difference in insulin concentrations. In this regard, it is also noteworthy that (at least in non-ruminants) AKT phosphorylates and stimulates sterol regulatory element binding transcription factor 1c (SREPB1c) leading to enhanced liver lipogenesis through the suppression of insulin induced protein 2 (INSIG2). INSIG2 is a protein of the endoplasmic reticulum that blocks the activation of SREBP1c by binding to SREBP cleavage-activating protein (SCAP) and prevents it from escorting SREBPs to the Golgi [52]. This scenario was confirmed in our experiment by the significant downregulation of *INSIG2* [FC = − 1.35], and the fact that at this age of the calf most of the supply of fatty acids (FA) are derived from consuming milk replacer.

The lower phosphorylated S6K1 (Ribosomal Protein S6 Kinase) ratio in MET calves was intriguing considering that S6K1 is the predominant regulatory kinase of GSK3 (Glycogen synthase kinase 3) [53] and that AKT is known to inactivate GSK3β [54], one of the two isoforms of GSK3. In this context, the significant downregulation of *PCK1* (*Phosphoenolpyruvate Carboxykinase 1*) [FC = − 1.99] is in line with the fact that most of the circulating glucose in newborn calves arises from lactose in the milk replacer fed. The downregulation of *FOXO4* (*Forkhead Box O4*) [FC = − 1.44] also agreed with this idea, since the FOXO complex binds the *PCK1* promoter [55].

It is well-established that lactose intake on its own is not sufficient to meet the newborn glucose demands [56], thus, calves have to establish endogenous glucose production and gluconeogenesis [57]. In this regard, hepatic glycogen stored during late-gestation represents an important energy source that is quickly exhausted [57, 58] until the gluconeogenic response is better developed. Typically, an increase of glucose-6-phosphatase (G6Pase) activity mirrors the decline of hepatic glycogen storage soon after birth [57]. This scenario was confirmed in our experiment by the significant downregulation of *GYS2* (*Glycogen Synthase 2*) [FC = − 1.39] and the simultaneous upregulation of *G6PC3* (*Glucose-6-Phosphatase Catalytic Subunit 3*) [FC = 1.30].

Mechanistically, greater activity of G6P phosphatase favors conversion of glucose-6-phosphate to glucose and ensures the release of glucose into the circulation [59]. Nevertheless, the downregulation of *PYGL* (*Glycogen Phosphorylase L*) [FC = − 1.32], along with upregulation of *NR0B2* (*Nuclear Receptor Subfamily 0 Group B Member 2*) [FC = 2.64] and *PPP2R3C* (*Protein Phosphatase 2 Regulatory Subunit B″ Gamma*) [FC = 1.68] was intriguing. These responses seemed to suggest a better capacity of MET calves in the utilization of lactose for meeting glucose requirements with a consequent reduction in depletion of glycogen reserves. The *PYGL* gene encodes a protein that catalyzes the cleavage of alpha-1,4-glucosidic bonds to release glucose-1-phosphate from liver glycogen stores and plays an important role in maintaining blood glucose homeostasis [60]. *NR0B2* encodes a protein that can directly bind to cAMP response element-binding protein (CREB) to block the association with CREB regulated transcription coactivator 2 (CRTC2), leading to an inhibition of hepatic gluconeogenic mRNA abundance [61, 62]. This was in line with the fact that AKT is known to phosphorylate and inhibit CRTC2 whose disruption is known to improve insulin sensitivity [63]. *PPP2R3C* encodes a subunit of the PP2A (protein phosphatase 2) holoenzyme involved in glucocorticoid receptor (GR) feedback regulation, and particularly in GR-mediated negative regulation [64]. In this regard, it is known that glucocorticoids in neonatal calves through activation of the GR can influence many important liver functions such as gluconeogenesis [65]. Particularly neonatal glucose metabolism when nutrient supply is insufficient [57]. Overall, this idea was also supported by our metabolomics results. Indeed, in absence of differences in feed intake, the greater concentrations of the tricarboxylic acid cycle intermediates fumarate and glutamate along with NAD/NADH in MET calves indicated enhanced rates of energy metabolism [26].

### Lipid metabolism

Lactose intake on its own is not sufficient to meet glucose demands [56] and mitochondrial FA oxidation increases after birth to cover energy needs [57]. In our experiment, we detected an overall downregulation of ‘Lipid metabolism’ in MET calves (Fig. 3; Additional File 5), and notably of ‘Fatty acid degradation’ genes such as *ACOX1* (*Acyl-CoA Oxidase 1*) [FC = − 1.53], *CPT1A* (*Carnitine Palmitoyltransferase 1A*) [FC = − 1.46], and *ACADM* (*Acyl-CoA Dehydrogenase Medium Chain*) [FC = − 1.68] all of which are well-known to be crucial for mitochondrial and peroxisomal FA oxidation [66]. There was also a significant downregulation of *PPARA* (*Peroxisome Proliferator Activated Receptor Alpha*) [FC = − 1.33] that encodes a nuclear receptor controlling genes required for activation of mitochondrial and peroxisomal FA oxidation [67]. In this context, the downregulation of *Gamma-Butyrobetaine Hydroxylase 1* (*BBOX1*) [FC = − 1.51], which in non-ruminants is regulated by PPARA [68], was noteworthy. This gene encodes the rate-limiting enzyme in the synthesis of carnitine from butyrobetaine [68], an essential nutrient for CPT1 activity and mitochondrial FA oxidation [69, 70]. In the absence of differences in hepatic concentration of butyrobetaine (Table 5) and fat intake, and because calves were consuming a high-fat milk replacer (15% crude fat on dry matter basis), the significant upregulation of *MCAT* (*Malonyl-Coa-Acyl Carrier Protein Transacylase*) [FC = 1.42] agreed with the downregulation of *CPT1A*. Indeed, *MCAT* is a component of the fatty synthase complex [71] and is specifically involved in the step where malonyl-CoA is loaded into the enzyme complex at the beginning of the FA biosynthesis process [72]. Considering that most of the FA found in the liver would have come as chylomicron remnants or from lipogenesis [73], it is plausible that lipogenesis from intake of lactose and glucose availability in the liver would have increased malonyl-CoA concentration, an allosteric inhibitor of *CPT1A* and mitochondrial FA oxidation [74]. Although we did not measure proteins involved in mitochondrial or peroxisomal FA oxidation, the downregulation of the above well-known FA oxidation genes that are transcriptionally-regulated suggested that the most logical alternative for the liver to handle FA coming from the diet in the MET calves might have been through synthesis of very low density lipoproteins (VLDL), phospholipid species, and/or incorporation into cellular membranes. The former hypothesis, formulated previously [75], was corroborated in our experiment by the tendency for greater mRNA abundance of *MAT1A* and *PEMT* (*P* value < 0.10; Table 7) in MET calves. These genes, indeed, play an important role in hepatic phosphatidylcholine (PC) synthesis [76–78], which is known to participate in VLDL synthesis and help prevent fatty liver [79, 80]. Endogenous synthesis of PC from supplemental Met [81] was already proposed to play a role in the ability of the cow liver to handle influx of FA produced from lipolysis after parturition [34]. More in-depth discussion about possible effects on increased VLDL export through the generation of PC is available in Additional File 4.

The marked downregulation of *Fatty Acid Desaturase 2* (*FADS2*) [FC = − 4.02] was also intriguing. This gene encodes the delta-6 desaturase enzyme, which catalyzes the first step in the desaturation and elongation of the dietary essential FA [82]. Interestingly, a different pool of PC molecular species is synthesized in the liver and is partly dependent on the pathway of synthesis: the cytidine diphosphocholine (CDP)-choline and phosphatidylethanolamine N-methyltransferase (PEMT) pathways [83]. PC synthesized via the PEMT pathway has higher concentrations of long-chain and highly-unsaturated species [84], whereas PC produced via the CDP-choline pathway has higher monounsaturated and saturated FA [85]. Thus, we speculate that lower *FADS2* expression may influence the availability of the preferred substrate required for liver PC synthesis in the PEMT pathway. In this regard, a diminished methylation capacity and hypermethylation silencing of *FADS2* mRNA expression has been observed in mice with CBS deficiency [86].

### Amino acid metabolism

Our functional analysis indicated that ‘Amino Acid metabolism’ was overall inhibited (Fig. 3; Additional File 5), due to marked inhibition of the ‘Valine, leucine and isoleucine degradation’ pathway. Leucine, isoleucine and valine are branched-chain amino acids (BCAA) with a number of biological functions beyond protein synthesis [87]. Furthermore, although little is known about the impact of changes in BCAA availability on immune system functionality in ruminants, both human and rodent studies have underscored the relationship between BCAA availability and improved immune function [88–91], highlighting the need for further studies about BCAA effects on dairy cows during the periparturient period and on newborn calves.

An increased concentration and utilization of essential AA, particularly BCAA, was observed in immortalized bovine mammary epithelial cells when the supply of Met increased [92]. The authors speculated this change was potentially mediated by alterations in AA transporters controlled by mTOR whose activation was observed when Met supply increased [92–94]. In this context, the significant upregulation of *Late Endosomal/Lysosomal Adaptor, MAPK and MTOR Activator* (*LAMTOR2*) [FC = 1.36] was noteworthy. The protein encoded by this gene is part of the regulatory complex involved in AA sensing and activation of mTORC1 [95], a signaling complex promoting cell growth in response to growth factors, energy level, and supply of AA [96].

Although a greater mRNA abundance of AA transporters was observed in the placenta of MET cows [24], which we speculated was partly due to greater DMI, overall, our results indicated that feeding RPM to enhance post-ruminal supply of MET did not affect hepatic AA transport in calf liver. In this regard, it is important to note that our calves were fed a high-protein milk replacer (28% crude protein on dry matter basis). Without differences in intake, our results suggested a lower utilization of AA in MET calf liver. This idea is supported by the lower p-S6K1, since it is well known the central role played by S6K1 in the regulation of protein translation in mammals [97]. The numerically-lower expression of pRPS6 (*P* value = 0.15) also partly corroborated this idea. Indeed, RPS6 was the first S6K substrate identified [98]. Considering our scenario of lower AA utilization by the liver, we speculate about the presence of possible AA ‘sparing effects’ in MET calves, i.e. more essential AA available for other organs, e.g. skeletal muscle for growth. This hypothesis agrees with the greater rate of growth already observed in utero and during early postnatal life in calves born to cows fed RPM to enhance post-ruminal Met supply during late gestation [26, 27].

### Glutathione metabolism

Although no significant differences between CON and MET calves were detected for GSH concentration, upregulation of *GSTO1* (*Glutathione S-Transferase Omega 1*) [FC = 2.85] and *GPX1* (*Glutathione Peroxidase 1*) [FC = 1.56; Additional File 2] both of which are involved in ‘Glutathione metabolism’ underscored the importance of this pathway in the MET neonatal liver. In non-ruminants, the role of GSH (a potent intracellular antioxidant in liver tissue) has been extensively described, in particular its association with methyl-transferase products involved in ‘Cysteine and Methionine metabolism’ [99]. In this regard, the tendency for greater taurine production in MET calves (*P* value = 0.06; Table 3) suggested possible beneficial effects on antioxidant responses during early postnatal life. An improved antioxidant status has been observed in peripartal dairy cows receiving enhanced post-ruminal Met supply [22, 35], and an increased in GPX activity also has been highlighted [100].

The idea that endogenous taurine plays a role in the antioxidant response of calves was formulated considering enzyme activity and metabolomics in the bigger cohort of calves from the present study [26]. It was further supported by data from an in vitro study in which taurine supplementation mitigated the inflammatory activation of blood polymorphonuclear leukocytes from neonatal Holstein calves [101]. More in general along with taurine, despite the lower CBS activity, the greater concentration of cystathionine and serine detected in MET calves suggested a greater activity of the transsulfuration pathway in MET calves, which is the main source of the cellular antioxidant GSH [40]. This scenario in our experiment supported the hypothesis of a better antioxidant status in MET calves, also suggested by the marked downregulation of *Catalase* (*CAT*) [FC = − 1.77; Additional File 2]. In humans, CAT is the most-adaptive antioxidant enzyme in the presence of oxidative stress and plays a pivotal role in cellular defense against oxidative damage [102].

The increase of antioxidant enzymes such as CAT can be considered a compensatory response to increased oxidative stress [103]. In non-ruminants, hepatic CAT activity increased during oxidative impairment due to an inhibition of CBS, although no variation in mRNA abundance [104] and no effect on GHS peroxidase, GHS reductase and GHS S-transferase activities were reported [104]. This latter pattern seemed to be recognizable in our experiment, suggesting that lower CBS activity was not associated with an alteration in epigenetic methylation as reported previously [105]. This is particularly noteworthy considering the role played by CBS in the transition from methylation to transsulfuration. More in-depth discussion about CBS activity is available in Additional File 4.

Overall, our results seemed to support the idea that feeding RPM to enhance post-ruminal supply of Met (along with other nutrients due to the greater DMI) may promote, through the transsulfuration pathway, production of the antioxidants GSH and taurine in calves [26]. The increased potential for antioxidant production may help alleviate oxidative stress and inflammation induced from reactive oxygen metabolite production in the liver [35, 106, 107] particularly at birth when antioxidant mechanisms in the new-born are still immature [108, 109]. Although downregulation of various genes associated with mitochondrial FA oxidation was observed in MET calves, the fact that high intracellular levels of FA are a source of reactive oxygen species [110] potentially via peroxisomal oxidation underscores the importance for production of antioxidants such as GSH and taurine. This hypothesis was also broadly supported in our experiment by the marked downregulation of ‘p53 signaling pathway’ (Fig. 5), since it is known that p53 activation is induced by multiple stress signals [111] and notably by oxidative stress [112].

Furthermore, considering that induction of thermogenic genes was recently observed as a consequence of taurine supplementation [113], and that liver is one important hub in thermogenesis [114], the overall upregulation of ‘Enrivonmental Adaptation’ function (Fig. 3; Additional File 5) due to changes in the ‘Thermogenesis’ pathway suggested that adaptive functions also might have been enhanced by feeding RPM to enhance post-ruminal supply of Met in the pregnant cow. The importance of thermoregulation in the newborn is well-establish as well as its linkage with energy sources available at birth [115], thus, it is plausible that a better energetic and immunometabolic status in MET calves may contribute to the thermoregulation.

### Immune system

New-born calves are immunologically naïve at birth [116], and particularly vulnerable since they have to face many stressors while their immune system is still immature [33]. Birth is the first direct and critical stressor in the calf’s life that has to be faced through innate immunity, a process that functions less effectively in adults, and passive immunity, i.e. the ingestion of colostrum [116]. In this regard, it is worth noting that feeding RPM in late-pregnancy does not seem to impact colostrum yield, colostral FA or AA profiles, colostral immunoglobulin G (IgG) concentrations, or apparent IgG absorption by the calf [27, 30], suggesting that neither the increase in post-ruminal Met nor total nutrient supply (due to greater DMI) affects the ability of the calf to acquire passive immunity. This is in line with the absence of difference in plasma GGT concentration in MET or CON calves (Table 2). Activity of GGT is a suitable indicator of passive immunity transfer in calves, and is highly- and positively-correlated with plasma IgG [117]. At least in non-ruminants, the liver is a key frontline immune tissue, balanced between an anti-inflammatory and immunotolerant status [118, 119]. In our study the overall upregulation of various pathways involved in ‘Immune system’ (Fig. 3; Additional File 5) and notably of ‘C-type lectin receptor signaling’ and ‘Natural killer cell mediated cytotoxicity’ pathways (Fig. 4) seemed to support our general hypothesis of an enhanced immunometabolic status in response to feeding RPM to enhance post-ruminal supply of Met in late-pregnancy. Indeed, these pathways are well-known to be involved in innate immunity [120–122].

The upregulation of the ‘Natural killer cell mediated cytotoxicity’ pathway was especially noteworthy considering that natural killer cells are mainly present in liver tissue and play a key role in the innate immune system [121, 123, 124]. Increasing evidence of cross talk between NK cells and other immune cells suggested that NK cells also play an important role in shaping the adaptive immune response [125–127]. On the other hand, C-type lectin receptors (CLR) are powerful pattern-recognition receptors that mediate immune recognition and response [128], whose role in orchestrating the induction of signaling pathways regulating adaptive immune responses is also well-recognized [129, 130].

The evident upregulation of the ‘Glycan Biosynthesis and Metabolism’ pathway (Fig. 3) agreed with the general scenario of an earlier and more effective immune status. Indeed, the role of glycans in the regulation of both innate and adaptive immune responses is well-documented in non-ruminants [131], in particular it is well-known that the immune system is highly controlled and fine-tuned by glycosylation through the addition of a diversity of carbohydrate structures (glycans) to the immune cell receptors [131]. This was consistent with the upregulation of the ‘C-type lectin receptor signaling’ pathway in our experiment, since C-type lectins are a class of glycans binding proteins [132]. In addition, considering the role of the phagosome in linking innate and adaptive immunity [133], the upregulation of the ‘Phagosome’ pathway (Fig. 4) also supported the hypothesis of an enhanced immune status in MET calves.

The general downregulation of ‘Platelet activation’ (Additional File 5) broadly agreed with this scenario, since liver dysfunction is usually reported in combination with an activation of platelets, often considered dynamic sentinels interacting with immune cells [134–136]. In this context, the marked upregulation of *M-SAA3.2* (*Mammary Serum Amyloid A3.2*) [FC = 5.70] was noteworthy (Table 8). SAA is an acute-phase protein which is highly-conserved among all vertebrate species [137]. In this regard, it is important to note that, if this response translated into more SAA protein secreted from the liver, it would mean that AA within liver would have been channeled away from protein synthesis, as suggested by the lower p-S6K1 observed in MET calves. Although the functional significance of SAA production remains speculative, several authors suggested its plausible role as an immunological defense molecule providing immediate defense against inflammatory challenges [138, 139], with a protective role during inflammation [140] and particularly with a possible beneficial function in the well-being and better extrauterine life adaptation in calves [141]. More in-depth discussion on single target genes involved in the general context of enhanced immune status is available in Additional File 4.

### Upstream regulators

Transcription factor enrichment analysis with the ChEA3 tool [142] allowed us to predict 72 TF significantly associated (FDR ≤ 0.05) with our DEG list (Additional File 3). A similar approach to the DIA analysis [143] was also applied to predict the potential impact and flux for each highlighted TF. Focusing on the top-10 ranked TF (Table 10) and based on information retrieved from the published literature, many of them are consistent with our general scenario of a higher immunometabolic state, greater insulin sensitivity and lower mitochondrial FA oxidation in MET calves. Among other TF identified were FOXO1 (Forkhead Box O1), PPARG (Peroxisome Proliferator Activated Receptor Gamma), E2F1 (E2F Transcription Factor 1), and CREB1 (CAMP Responsive Element Binding Protein 1). All these have relatively well-established associations with important metabolic and cellular processes in non-ruminants.

At least in non-ruminants, FOXO1 is a key TF associated with coordinating lipid metabolism in the post-absorptive state, integrating insulin signaling with glucose and lipid metabolism [144]. In liver, insulin activates the PI3K/PKB signaling pathway [145] and results in FOXO1 protein phosphorylation and degradation [146]. FOXO1 binds and promotes transcription of *PCK1*, which plays a key role in gluconeogenesis [146]. Indeed, hepatic FOXO1 loss-of-function mutant suppresses *PCK1* expression determining a decreased hepatic gluconeogenesis [146]. Also the regulation of pyruvate dehydrogenase kinase (PDK) expression by FOXO signaling has been recently investigated and the inhibition of PDK by insulin via phosphorylation of FOXO through PI3K/Akt signaling pathway has been described [147, 148]. The downregulation of *PDK1* [FC = − 1.45] in our experiment was in line with this scenario. Furthermore, at least in non-ruminants, FOXO1 regulates transcription of genes involved in hepatic assembly of VLDL [149] and also plays a fundamental role in development and differentiation of immune cells [150].

The nuclear receptor PPARG belongs to peroxisome proliferator-activated receptors, members of the superfamily of ligand-activated nuclear transcription factors. Many PPAR-regulated genes encode proteins that regulate FA oxidation (mitochondrial and peroxisomal) and storage, for example, *ACOX1* and *CPT1A*. Furthermore, PPARG regulates pantothenate kinase (PANK) [151], which catalyzes the rate-controlling step in coenzyme A (CoA) biosynthesis [152]. In our experiment, we detected downregulation of *PANK3* [FC = − 1.87], which would be metabolically important because CoA is an essential cofactor supporting a multitude of oxidative and synthetic reactions, including those involved in FA biosynthesis and oxidation [153]. Taking all of this into account, the predicted inhibition of PPARG seemed particularly in line with our general scenario of FA oxidation downregulation.

Among the list of top-10 ranked TF, the presence of E2F1, a well-known TF promoting hepatic gluconeogenesis [154] and innate immune response [155] is noteworthy. Similarly, the predicted activation of CREB1 whose role in coordinating hepatic lipid and glucose metabolism through inhibition of PPARG has been documented [156] along with its role in the immune system through promoting anti-inflammatory responses [157].

## Conclusions

Feeding RPM to enhance maternal post-ruminal Met supply was associated with greater global DNA methylation and distinct hepatic transcriptome, proteome, and metabolome profiles after birth. The hypothesis that enhancing post-ruminal Met supply during late-gestation primed or programmed the Met cycle in calf liver was corroborated. However, in light of the greater DMI in response to feeding RPM (a consistent response across published studies), the increased supply of other nutrients and their potential impact cannot be disregarded. Molecular changes with potential beneficial effects to overall hepatic function were highlighted, especially in terms of metabolism and inflammatory status. This was attributable to the change in expression profiles of several genes mainly involved in ‘Glucose’, ‘Lipid’, ‘Glutathione’, and ‘Immune System’ metabolism. In particular, the results seemed to suggest a greater flux through the TCA cycle along with a better antioxidant status due to taurine and GSH synthesis. Although further studies are required to corroborate this result, the upregulation of ‘Immunity System’ pathways supported the hypothesis that feeding RPM to enhance maternal Met supply may play a role in regulating fetal immunity.

## Methods

### Animals

All procedures for this study were conducted in accordance with a protocol approved by the Institutional Animal Care and Use Committee (IACUC) of the University of Illinois (protocol # 14270). Heifer calves in the present study were a subset from a larger cohort of animals born to Holstein cows randomly assigned to receive a basal control (CON) close-up diet (from − 28 ± 2 d to parturition) [1.47 Mcal/kg dry matter (DM) and 15.3% crude protein (CP)] with no added Met or CON plus ethyl cellulose MET (MET, Mepron®, Evonik Nutrition & Care GmbH, Germany) [27]. Diet composition is available in Additional File 6. All management procedures have been described previously [27]. Briefly, during the preliminary period from − 45 to − 29 d relative to parturition all cows received a common low-energy and high-straw far-off diet (1.33 Mcal/kg of DM and 13.9% CP) with no RPM. Cows were individually-fed using Calan gates (American Calan Inc., Northwood, NH). At − 28 days relative to parturition, cows were randomly assigned to CON or MET groups. The Met product was offered at a rate of 0.09% of previous day DMI. This supply of Met was based on studies demonstrating beneficial effects on production performance and health during the prepartum period [23, 158]. Mepron® is a commercial rumen-protected source of DL-Met in the form of small beads containing a minimum of 85% methionine that resists ruminal degradation due to an ethyl-cellulose film coating the methionine core. The intestinal digestibility coefficient of Mepron® is 90% [159] and its ruminal bypass value is 80% [160]. Feeding this amount of RPM increased Met concentrations in plasma prior to and during the first 3 weeks postpartum [25].

After parturition, neonatal calves were separated from their dams. Body weight (BW), hip and wither height (HH, WH), hip width (HW) and body length were measured. Heifer calves were kept in the experiment if they fulfilled all the following criteria previously described [30], 1) single heifer calf; (2) heifer calf birth weight > 36 kg; (3) calving difficulty score < 3; and (4) dam first colostrum volume > 3.8 L. All calves were managed in the same fashion. At birth, the navel was disinfected with 7% tincture of iodine solution (First Priority Inc., Elgin, IL, United States), and neonatal calves were vaccinated with TSV II (Pfizer Inc., New York, NY, United States) via nostril application.

Calves were offered 3.8 L of first milking colostrum from their mother within 6 h. If voluntary colostrum intake had not reached the 3.8 L required, calves were force-fed via an esophageal tube to ensure that all calves consumed the same amount of colostrum. Calves were housed in individual outdoor hutches bedded with straw and fed twice daily (AM and PM) with a milk replacer (Advance Excelerate, Milk Specialties, Carpentersville, IL; 28.5% CP, 15% fat). More in detail, heifer calves received 4.54 kg/day of milk replacer mix (0.59 kg of milk replacer in 3.95 L of water) and had ad libitum access to starter grain mix [Ampli-Calf Starter 20®; 19.9% CP and 13.5% neutral detergent fiber (NDF), Purina Animal Nutrition, Shoreview, MN, United States] fed in the morning. All heifer calves remained clinically healthy during the study.

### Sample collection

Per IACUC guidelines, blood samples were collected (*n* = 6 calves per group) from the jugular vein at 2 d of age using 20-gauge BD Vacutainer needles (Becton Dickinson, Franklin Lakes, NJ). Vacutainer tubes used contained lithium heparin, and plasma was obtained by centrifugation at 1900×g for 15 min at 4 °C and stored at − 80 °C until further analysis. Plasma was used to analyze the concentrations of a number of biomarkers associated with energy metabolism (e.g. NEFA, BHBA), immune function, liver function, and oxidant status according to protocols described in a number of publications from our group [9, 10, 30, 31]. In addition, plasma was used to profile concentrations of free AA and indicators of skeletal muscle protein turnover [27]. Plasma insulin concentrations were determined using a bovine-specific commercial ELISA kit (catalog no. 10–1201-01; Mercodia, Uppsala, Sweden).

Per IACUC guidelines, surgical biopsies of the liver could not be obtained until day 4 of age. At that point, samples were obtained via puncture biopsy under local anesthesia (n = 6 calves per group) using published protocols from our group [26, 31]. Tissue was frozen immediately in liquid nitrogen and stored at − 80 °C until further analysis. Samples from the same calves (n = 6 per treatment) used for DNA methylation and RNA-sequencing were used for protein expression, enzyme activity, and metabolomics analyses. The chosen protein targets are key members of the nutrient signaling cascades that respond to insulin and AA supply, hence, allowed us to evaluate potential effects of maternal MET on key metabolic aspects of neonatal liver function. Since newborn calves do not have a functional rumen, insulin plays an essential role on aspects of hepatic nutrient metabolism including coordination of glucose, FA, and AA metabolism [57]. Similarly, since our previous work with neonatal calves revealed unique adaptations in hepatic one-carbon metabolism in response to feeding RPM in late-pregnancy [10, 30], we elected to perform those assays along with targeted metabolomics analysis.

### DNA isolation and global DNA methylation

The DNA was isolated from 50 mg of liver tissue using the Blood and Tissue DNeasy Kit (Qiagen). Methylation of genomic DNA was detected using the Methylamp global DNA methylation quantification ultra-kit (Epigentek) according to manufacturer’s instructions. Details on calculation were reported previously by our group [161].

### Western blotting

The protocol used for western blot was the same as reported previously [162]. Briefly, 50 g of liver tissue was used for total protein extraction using T-PER tissue protein extraction reagent (Cat. 78,510, Thermo Scientific) containing Halt protease and phosphatase inhibitor cocktail (100x, catalog no. 78442; Thermo Fisher Scientific). Concentration of total protein was determined using the Pierce BCA protein assay kit (catalog no. 23227; Thermo Fisher Scientific). Equal amounts of protein were separated using 4–20% Mini-Protean TGX precast gels (Cal. 4,561,096, Bio-rad) and transferred onto a PVDF membrane (Cat. 1,620,261, Bio-rad) using a semidry transfer cell (Serial No. 221BR, Bio-rad). After blocking with 5% non-fat dry milk in trisbuffered saline at room temperature for 2 h, the membrane was washed and incubated with a primary antibody directed against target antibodies. Antibodies raised from rabbit monoclonal antibody were from Cell Signaling Technology and diluted to 1:1000 prior to use [25, 92]. Targets were mTOR, p-mTOR (Ser2448), S6K, p-S6K (Thr389), RPS6, p-RPS6 (Ser235/236), 4EBP1, p-4EBP1 (Thr37/46), Akt, p-Akt (Ser473), eIF2, p-eIF2 (Ser51), eEF2, and p-eEF2 (Thr56) (catalog nos. 2972, 2971, 9202, 9234, 2217, 2211, 9452, 9459, 9272, 9271, 9722, 9721, 2332, and 2331, respectively; Cell Signaling Technology). The secondary antibody was HRP AffiniPure goat anti-rabbit IgG (ab6721, Abcam, diluted to 1:10,000). Visualization of the target proteins was performed using the Clarity Western ECL Substrate (Cat. 170–5060, Bio-rad). Each protein was normalized against GAPDH (Polyclonal antibodies raised in rabbit, ab22555, Abcam, diluted 1:2000). Data are reported as relative expression. The ECL signals were recorded using an imaging system (ChemiDocTM MP, Bio-rad) and analyzed using Image lab 5.2.1 (Bio-Rad, USA).

### Metabolomics

Approximately 100 mg of frozen tissue was extracted using a 2-step protocol described by Wu et al. [163]. Targeted metabolomics (liquid chromatography–MS) was performed to quantify 31 metabolites related to TCA cycle, one-carbon metabolism, and transsulfuration pathway. Samples were analyzed with the 5500 QTRAP liquid chromatography–tandem MS system (Sciex, Framingham, MA) at the metabolomics core facility of the Roy J. Carver Biotechnology Center, University of Illinois (Urbana). Software Analyst 1.6.2 [161] was used for data acquisition and analysis.

### One-carbon metabolism enzyme activity assays

Activity of BHMT, MTR, and CBS was determined according to protocols detailed by Zhou et al. [164].

### RNA extraction and quantitative PCR

All RNA extraction procedures were performed as described previously [26]. Briefly, RNA extracted from liver samples using the miRNeasy kit (Qiagen, Hilden, Germany) following the manufacturer’s protocol. Samples were treated on-column with DNaseI (Qiagen) to remove genomic DNA from the RNA. Quantification was accessed using the NanoDrop ND-1000 (NanoDrop Technologies, Rockland, DE), and RNA quality was measured using an Agilent 2100 Bioanalyzer (Agilent, Santa Clara, CA). All samples had an RNA integrity number factor greater than 8, RNA was diluted to 100 ng with DNase/RNase-free water for cDNA synthesis by using RT-PCR, and the cDNA was diluted 1:4 with DNase/RNase-free water.

The quantitative PCR (qPCR) performed was SYBER Green based (Quanta Biosciences, Beverly, MA), using a 7-point standard curve obtained from a cDNA pool of all samples. The final data were normalized using the geometric mean of 3 internal control genes: UXT, GAPDH, and RPS9. A total of 24 genes selected related to the Met cycle, DNA methylation, transsulfuration, GSH, and cytidine 5′-diphosphocholine (CDP)–choline pathway were measured. The log_2_ transformed data were analyzed in order to compare the expression means of CON and MET groups. All evaluated genes and primer information are reported in Additional File 7.

### Statistical analysis

Data were assessed for normality of distribution using the Shapiro-Wilk test. When the normality assumption was rejected, data were log_2_-transformed before statistical analysis and log_2_-back transformed after analysis. Differences between the means of the groups were analyzed via one-way ANOVA. Statistical differences were declared significant at *P* value ≤ 0.05 and tendencies at *P* value ≤ 0.10. All statistical analyses were performed in R environment [165].

### RNA-sequencing

Sequencing was performed by the High-Throughput Sequencing and Genotyping Unit of the W. M. Keck Biotechnology Center at the University of Illinois at Urbana Champaign (Urbana, IL, USA). A total of 12 mRNA libraries were prepared with Illumina’s ‘TruSeq Stranded Sample Prep kit’ following the manufacturer’s instructions. Libraries were sequenced on one lane for 101 cycles from one end of the fragments on a HiSeq2500 (Illumina Inc.), using HiSeq SBS sequencing kit version 4. Fastq files were generated and demultiplexed with the bcl2fastq (v2.17.1.14) software (Illumina). Approximately a total of 143 million single-reads sequences of 100 nt in length were collected. The quality control of the raw sequences was assessed with FastQC software (v0.11.15) and the reads were mapped to the bovine reference genome (UMD v.3.1.1; Ensembl Genomes website) using the software package STAR (v2.5.3a). The number of reads that mapped to each gene in each sample was calculated using the feature Counts function in the Subread package (v1.5.2). Data are available at the GEO site of NCBI (GSE163644).

### Identification of differentially expressed genes

The entire downstream analysis for identification of DEGs was conducted within the R environment using the edgeR pipeline [166]. Non-expressed and weakly-expressed genes were removed [167], TMM (trimmed mean of M-values) normalization was applied to all samples and the data were log_2_-transformed. Once dispersion estimates were obtained and a negative binomial generalized linear model was fitted with a design matrix describing the treatment condition (MET and CON groups), quasi-likelihood F-test was used to determine differential expression. Multiplicity correction was performed by applying the Benjamini-Hochberg method on the *P* values, to control the false discovery rate (FDR). The expression level was deemed to be significantly different between the two groups for FDR ≤ 0.10 [32].

### Dynamic impact approach (DIA)

The DIA software was used for functional analyses [143]. Briefly, DIA uses the KEGG systems information to rank biological pathways, calculating the overall impact (importance of a given pathway) and flux (direction of impact; e.g., upregulation, downregulation, or no change) based on gene fold change (FC) values. For this purpose, the entire DEG data set (FDR ≤ 0.10) with associated statistical *P* values was imported into DIA and the functional analysis was performed interrogating all KEGG pathway database.

### Gene network analysis

The PANEV (Pathway Network Visualizer) tool [168] was extensively used to visualize the results in a context of gene/pathway networks, and pinpoint candidate genes associated with a subset of pathways of interest. Briefly, PANEV is an R package set for gene/pathway-based network visualization. Based on information available on KEGG, it visualizes genes within a customized network of multiple interconnected pathways, chosen by the user since supposed to be involved with the trait under investigation. The network graph visualization helped us to interpret functional profiles of cluster of genes. In our case, a network of interconnected (i.e. up- and downstream) pathways involved in ‘Glycolysis/gluconeogenesis’, ‘Lipid metabolism’, ‘Amino acid metabolism’ and ‘Immune system’ pathways was created to pinpoint functional candidate genes with coordinate expressions (Additional File 8).

### Transcription regulator discovery

ChIP-X Enrichment Analysis 3 (ChEA3) tool [142] was used to predict the TF associated with the downstream DEG. ChEA3 is a transcription factor enrichment analysis software that ranks TF associated with user-submitted gene sets [142]. The entire list of DEG (FDR ≤ 0.10) was uploaded on ChEA3 and the ‘Literature ChIP-seq’ library, containing ChIP-seq experiments from human, mouse and rat, was interrogated. ChEA3 core analysis was run using default settings and TF with FDR ≤ 0.05 were considered significantly associated with our DEG list. Furthermore, in order to estimate the state (i.e. activation/inhibition) of significantly-predicted TF, the same approach underlying DIA analysis [143] was applied to the candidate TF list. Briefly, impact and flux value were calculated for each predicted TF according to Bionaz et al. [143]: the impact was obtained by combining the proportion of DEG with the log_2_ mean fold change and mean –log P value of all TF-target genes. More details about rationale and calculation steps of the analysis are reported in the original publication [143]. The ChEA3 outcomes were matched with PANEV results in order to create a network of possible connections between TF and the downstream DEG involved with the ‘Glycolysis/gluconeogenesis’, ‘Lipid metabolism’, ‘Amino acid metabolism’ and ‘Immune system’ pathways (Additional File 9).

## Supplementary Information

**Additional File 1: **Summary of RNA sequencing and alignment for all the samples. mRNA libraries were sequenced on a HiSeq2500 (Illumina Inc.). Quality control metrics were performed on raw sequencing reads using the FASTQC (v0.11.15) application. An index of the reference genome was built and single-end clean reads for each individual were aligned to the reference genome by STAR (v2.5.3a). Reads were mapped and annotated to the *Bos Taurus* UMD_3.1.1, downloaded from Ensembl Genomes website. Reads aligned were quantified with Subread package (v1.5.2) based on the Refseq gene annotation.

**Additional File 2: **List of all differentially expressed genes (FDR ≤ 0.10) from RNAseq data in liver tissue of 4-d old Holstein calves (*n* = 6/group) born to cows randomly assigned to receive a basal control (CON) diet from − 28 ± 2 d to parturition [1.47 Mcal/kg dry matter (DM) and 15.3% crude protein (CP)] with no added Met or CON plus ethyl cellulose Met (MET, Mepron®, Evonik Nutrition & Care GmbH, Germany).

**Additional File 3: **List of all transcription factor (TF) obtained with ChEA3 transcription factor enrichment analysis tool and significantly overrepresented (FDR ≤ 0.05) in the differentially expressed gene (DEG) list from RNAseq data in liver tissue of 4-d old Holstein calves (n = 6/group) born to cows randomly assigned to receive a basal control (CON) diet from − 28 ± 2 d to parturition [1.47 Mcal/kg dry matter (DM) and 15.3% crude protein (CP)] with no added Met or CON plus ethyl cellulose Met (MET, Mepron®, Evonik Nutrition & Care GmbH, Germany). For each TF, the ranking (Rank), *P* value, false discovery rate (FDR), the overlapping genes among the DEG list, and the predicted state (i.e. impact and flux value) calculated using the rationale underlying the Dynamic Impact Approach (DIA) analysis are reported.

**Additional File 4:.** Extended discussion on methionine synthase (MTR) and cystathionine beta-synthase (CBS) activity, phosphatidylcholine (PC) and very low density lipoproteins (VLDL), and on single genes involved in immune status in MET calves.

**Additional File 5: **KEGG pathways in ‘Lipid Metabolism’, ‘Amino Acid Metabolism’, and ‘Immune System’ subcategories resulting from the DIA analysis of RNAseq data from liver tissue of 4-d old Holstein calves (*n* = 6/group) born to cows randomly assigned to receive a basal control (CON) diet from − 28 ± 2 d to parturition [1.47 Mcal/kg dry matter (DM) and 15.3% crude protein (CP)] with no added Met or CON plus ethyl cellulose Met (MET, Mepron®, Evonik Nutrition & Care GmbH, Germany). The columns represent the effect (impact) and flux responses. The blue bars represent the effect value (0 to 300), and the flux columns represent negative (−) and positive (+) flux (− 300 to + 300) based on the direction of the effect. The positive flux (red bars) indicates an upregulation in treated (MET) liver tissue cells compared to control (CON) ones, while the negative flux (green bars) indicates a downregulation.

**Additional File 6:.** Ingredient and nutrient composition of far-off (from − 45 to − 29 d) and close-up (from − 28 d to parturition) diets fed to Holstein cows used in the present study.

**Additional File 7:.** Extended information about quantitative real-time PCR. Accession number, gene symbol, and forward and reverse primer sequences of genes analyzed in calf liver.

**Additional File 8:.** Gene/pathway-based network visualization obtained with Pathway Network Visualizer (PANEV) tool, considering the entire list of differentially expressed genes (FDR ≤ 0.10). The network was created taking into account the main pathways of interest involved in ‘Glycolysis/gluconeogenesis’, ‘Lipid metabolism’, ‘Amino acid metabolism’ and ‘Immune system’, along with the list of their interconnected (i.e. up- and downstream) pathways based on information retrieved on KEGG pathway database.

**Additional File 9:.** Transcription Factor/Gene/pathway-based network visualization matching the outcomes from Pathway Network Visualizer (PANEV) tool with the main results obtained by ChIP-X Enrichment Analysis 3 (ChEA3) software.

## Data Availability

The raw sequencing data of 12 animals in this study are available from the GEO site of NCBI under accession number GSE163644.

## Abbreviations

4EBP1: Eukaryotic translation initiation factor 4E-binding protein 1
AA: Amino acid
ACADM: Acyl-CoA dehydrogenase medium chain
ACOX1: Acyl-CoA oxidase 1
AKT: Serine/threonine-protein kinases
AM: Ante meridiem
ANGPTL3: Angiopoietin-like protein 3
ANOVA: Analysis of variance
APOB: Apolipoprotein B
BBOX1: Butyrobetaine hydroxylase 1
BCAA: Branched-chain amino acids
BHBA: Beta-hydroxybutyric acid
BHMT: Betaine-homocysteine S-methyltransferase
BW: Body weight
CAT: Catalase
CBS: Cystathionine β-synthase
CCNB1: Cyclin B1
CD14: CD14 molecule
CD209: CD209 molecule
CDK1: Cyclin-dependent kinase 1
cDNA: Complementary deoxyribonucleic acid
CDO1: Cysteine dioxygenase type 1
CDP: Cytidine diphosphate
ChEA3: ChIP-X enrichment analysis 3
CLEC6A: C-type lectin domain containing 6A
CLR: C-type lectin receptors
CON: Control
CPT1A: Carnitine palmitoyltransferase 1A
CREB1: CAMP responsive element binding protein 1
CRTC2: CREB regulated transcription coactivator 2
DEG: Differentially expressed gene
DIA: Dynamic impact approach
DMI: Dry matter intake
DNA: Deoxyribonucleic acid
dTMP: Deoxythymidine monophosphate
dUMP: Deoxyuridine monophosphate
E2F1: E2F transcription factor 1
ECL: Emitter-coupled logic
eEF2: Eukaryotic elongation factor 2
eIF2: Eukaryotic initiation factor 2
FA: Fatty acid
FADS2: Fatty acid desaturase 2
FDR: False discovery rate
FOXO1: Forkhead transcription factor 1
FOXO4: Forkhead box O4
G6Pase: Glucose-6-phosphatase
G6PC3: Glucose-6-phosphatase catalytic subunit 3
GAPDH: Glyceraldehyde-3-phosphate dehydrogenase
GCL: Glutamate–cysteine ligase
GCLM: Glutamate-cysteine ligase modifier subunit
GPX1: Glutathione peroxidase 1
GR: Glucocorticoid receptor
GSH: Glutathione
GSK3: Glycogen synthase kinase 3
GSTO1: Glutathione S-transferase omega 1
GYS2: Glycogen synthase 2
HH: Hip height
HIST1H2AC: H2A clustered histone 6
HRP: Horseradish peroxidase
HW: Hip width
IACUC: Institutional animal care and use committee
IgG: Immunoglobulin G
INSIG2: Insulin induced protein 2
KEGG: Kyoto encyclopedia of genes and genomes
KLF5: Krüppel-like factor 5
LAMTOR2: Late endosomal/lysosomal adaptor, MAPK and MTOR activator
LIPH: Lipase H
LPA: Lipoprotein A
LPL: Lipoprotein lipase
MAPK: Mitogen-activated protein kinase
MAT1A: Methionine adenosyltransferase 1A
MAT2B: Methionine adenosyltransferase 2B
MCAT: Malonyl-CoA-acyl carrier protein transacylase
Met: Methionine
MET: Maternal methionine supplied
mRNA: Messenger ribonucleic acid
M-SAA3.2: Mammary serum amyloid A3.2
mTORC1: Mechanistic target of rapamycin kinase 1
MTR: 5-methyltetrahydrofolate homocysteine methyltransferase
NAD: Nicotinamide adenine dinucleotide
NADH: Nicotinamide adenine dinucleotide plus hydrogen
NDF: Neutral Detergent Fiber
NEFA: Non-esterified fatty acids
NK: Natural killer
NR0B2: Nuclear receptor subfamily 0 group B member 2
NR3C1: Nuclear receptor subfamily 3 group C member 1
p-4EBP1: Phosphorylated eukaryotic translation initiation factor 4E-binding protein 1
p-AKT: Phosphorilated serine/threonine-protein kinases
PANEV: Pathway network visualizer tool
PANK: Pantothenate kinase
PC: Phosphatidylcholine
PCK1: Phosphoenolpyruvate carboxykinase 1
PCYT1B: Phosphate cytidylyltransferase 1, choline, beta
PDK: Pyruvate dehydrogenase kinase
p-eEF2: Phosphorylated eukaryotic elongation factor 2
p-eIF2: Phosphorylated eukaryotic initiation factor 2
PEMT: Phosphatidylethanolamine N-methyltransferase
Phe: Phenylalanine
PI3K: Phosphoinositide 3-kinases
PKB: Protein kinase B
PLPP3: Phospholipid phosphatase 3
PM: Post meridiem
p-mTOR: Phosphorylated mechanistic target of rapamycin kinase.
POLR2I: RNA polymerase II subunit I
POLR3H: RNA polymerase III subunit H
PP2A: Protein phosphatase 2
PPARA: Peroxisome proliferator activated receptor alpha
PPARG: Peroxisome proliferator sctivated receptor gamma
PPP2R3C: Protein phosphatase 2 regulatory subunit B″ gamma
p-RPS6: Phosphorylated ribosomal protein S6
p-S6K1: Phosphorylated ribosomal protein S6 kinase beta-1
PYGL: Glycogen phosphorylase L
qPCR: Quantitative polymerase chain reaction
QTRAP: Quadropule ion trap
REC8: REC8 meiotic recombination protein
RNA: Ribonucleic acid
RNA-Seq: RNA sequencing
RPM: Rumen-protected methionine
RPS6: Ribosomal protein S6
RPS9: Ribosomal protein S9
RT-PCR: Real-time polymerase chain reaction
S6K: Ribosomal S6 kinase
S6K1: Ribosomal protein S6 kinase beta-1
SAM: S-adenosylmethionine
SCAP: SREBP cleavage-activating protein
SLC44A4: Solute carrier family 44 member 4
SLC51A: Solute carrier family 51 subunit alpha
SREBP1: Sterol-regulated element-binding protein
SREPB1c: Sterol regulatory element binding transcription factor 1c
Tau: Taurine
TCA: Tricarboxylic acid cycle
TF: Transcription factor
THF: Tetrahydrofolate
TNF: Tumor necrosis factor
TRMT61A: tRNA methyltransferase 61A
TYMS: Thymidylate synthase
UXT: Ubiquitously expressed prefoldin like chaperone
VLDL: Very low density lipoproteins
WH: Wither height
