# Supplementary material for: Unique adaptations in neonatal hepatic transcriptome, nutrient signaling, and one-carbon metabolism in response to feeding ethyl cellulose rumen-protected methionine during late-gestation in Holstein cows
Source: BMC Genomics. 2021 Apr 17;22:280. doi: 10.1186/s12864-021-07538-w (PMC8053294; doi:10.1186/s12864-021-07538-w)
Supplement: Supplementary file 1 — Additional File 1: Summary of RNA sequencing and alignment for all the samples. mRNA libraries were sequenced on a HiSeq2500 (Illumina Inc.). Quality control metrics were performed on raw sequencing reads using the FASTQC (v0.11.15) application. An index of the reference genome was built and single-end clean reads for each individual were aligned to the reference genome by STAR (v2.5.3a). Reads were mapped and annotated to the Bos Taurus UMD_3.1.1, downloaded from Ensembl Genomes website. Reads aligned were quantified with Subread package (v1.5.2) based on the Refseq gene annotation. [file 12864_2021_7538_MOESM1_ESM.docx]

**Additional File 1:** Summary of RNA sequencing and alignment for all the samples. mRNA libraries were sequenced on a HiSeq2500 (Illumina Inc.). Quality control metrics were performed on raw sequencing reads using the FASTQC (v0.11.15) application. An index of the reference genome was built and single-end clean reads for each individual were aligned to the reference genome by STAR (v2.5.3a). Reads were mapped and annotated to the Bos Taurus UMD_3.1.1, downloaded from Ensembl Genomes website. Reads aligned were quantified with Subread package (v1.5.2) based on the Refseq gene annotation.

| **Sample ID** | **Treatment** | **Total Reads** | **Total Mapped Reads** | **Reads mapped to annotated exons** |
| --- | --- | --- | --- | --- |
| 1 | CON | 12,221,828 | 11,639,884 | 9,500,438 |
| 2 | CON | 11,168,823 | 10,640,267 | 8,906,764 |
| 3 | CON | 10,879,061 | 10,400,778 | 8,277,789 |
| 4 | CON | 11,758,882 | 11,205,120 | 9,111,404 |
| 5 | CON | 11,772,012 | 11,253,914 | 9,312,036 |
| 6 | CON | 11,657,305 | 11,129,228 | 9,317,134 |
| 1 | MET | 13,196,788 | 12,549,985 | 10,397,604 |
| 2 | MET | 11,705,537 | 11,142,667 | 8,954,783 |
| 3 | MET | 12,689,657 | 12,068,463 | 9,975,314 |
| 4 | MET | 11,332,005 | 10,755,817 | 9,163,920 |
| 5 | MET | 12,411,897 | 11,776,700 | 10,010,787 |
| 6 | MET | 12,272,518 | 11,650,398 | 9,770,316 |
