# Supplementary material for: Unique adaptations in neonatal hepatic transcriptome, nutrient signaling, and one-carbon metabolism in response to feeding ethyl cellulose rumen-protected methionine during late-gestation in Holstein cows
Source: BMC Genomics. 2021 Apr 17;22:280. doi: 10.1186/s12864-021-07538-w (PMC8053294; doi:10.1186/s12864-021-07538-w)
Supplement: Supplementary file 2 — Additional File 2: List of all differentially expressed genes (FDR ≤ 0.10) from RNAseq data in liver tissue of 4-d old Holstein calves (n = 6/group) born to cows randomly assigned to receive a basal control (CON) diet from − 28 ± 2 d to parturition [1.47 Mcal/kg dry matter (DM) and 15.3% crude protein (CP)] with no added Met or CON plus ethyl cellulose Met (MET, Mepron®, Evonik Nutrition & Care GmbH, Germany). [file 12864_2021_7538_MOESM2_ESM.docx]

**Additional File 2:** List of all differentially expressed genes (FDR ≤ 0.10) from RNAseq data of liver tissue of calves (n = 6/group) from Holstein cows randomly assigned to receive a basal control (CON) close-up diet (from −28 ±2 d to parturition) [1.47 Mcal/kg dry matter (DM) and 15.3% crude protein (CP)] with no added Met or CON plus ethyl cellulose Met (MET, Mepron®, Evonik Nutrition & Care GmbH, Germany).

| **Entrez ID** | **Ensembl ID** | **Gene symbol** | **FC** | **FDR** | **P value** |
| --- | --- | --- | --- | --- | --- |
| 100125834 | ENSBTAG00000038375 | *STOM* | -1.43 | 0.037 | 1.82E-05 |
| 104968464 | ENSBTAG00000050907 | *RPP21* | 2.09 | 0.037 | 1.10E-05 |
| 281111 | ENSBTAG00000051907 | *CYBA* | 1.42 | 0.037 | 4.60E-05 |
| 281644 | ENSBTAG00000021867 | *BFSP1* | 3.42 | 0.037 | 1.21E-05 |
| 282360 | ENSBTAG00000002348 | *SLC4A4* | -1.73 | 0.037 | 4.62E-05 |
| 508918 | ENSBTAG00000003746 | *SCP2* | -1.79 | 0.037 | 1.93E-05 |
| 509304 | ENSBTAG00000007933 | *DIPK2A* | -1.75 | 0.037 | 4.09E-05 |
| 510749 | ENSBTAG00000011895 | *PANK3* | -1.87 | 0.037 | 4.16E-05 |
| 511968 | ENSBTAG00000018918 | *NDUFAF3* | 1.54 | 0.037 | 9.65E-06 |
| 512028 | ENSBTAG00000001535 | *LIPH* | -1.75 | 0.037 | 2.90E-05 |
| 519557 | ENSBTAG00000011569 | *AADAC* | -1.65 | 0.037 | 3.38E-05 |
| 531682 | ENSBTAG00000020980 | *CAT* | -1.77 | 0.037 | 2.14E-05 |
| 538436 | ENSBTAG00000004906 | *CCNE2* | -2.72 | 0.037 | 1.22E-05 |
| 614472 | ENSBTAG00000015002 | *POLR2I* | 1.71 | 0.037 | 1.78E-05 |
| 615490 | ENSBTAG00000013176 | *SMOC2* | -2.29 | 0.037 | 2.78E-05 |
| 767843 | ENSBTAG00000005311 | *POLR3H* | 1.72 | 0.037 | 4.77E-05 |
| 783869 | ENSBTAG00000021373 | *GTF3C5* | 1.47 | 0.037 | 4.62E-05 |
| 788119 | ENSBTAG00000006366 | *NFATC4* | 1.60 | 0.037 | 3.13E-05 |
| 287026 | ENSBTAG00000008338 | *PLCB1* | -1.50 | 0.039 | 6.04E-05 |
| 507007 | ENSBTAG00000009560 | *NUBP1* | 1.41 | 0.039 | 6.73E-05 |
| 510283 | ENSBTAG00000031327 | *TRMT61A* | 1.70 | 0.039 | 6.95E-05 |
| 510373 | ENSBTAG00000013988 | *BID* | 1.43 | 0.039 | 5.84E-05 |
| 539334 | ENSBTAG00000013205 | *IL1RAP* | -1.57 | 0.039 | 6.86E-05 |
| 617707 | ENSBTAG00000011640 | *PLPP3* | -1.84 | 0.039 | 6.59E-05 |
| 782615 | n/a | *n/a* | -1.88 | 0.039 | 6.53E-05 |
| 537188 | ENSBTAG00000002701 | *UGT3A2* | -1.54 | 0.040 | 7.40E-05 |
| 100125882 | ENSBTAG00000049300 | *AARSD1* | 1.46 | 0.040 | 9.23E-05 |
| 101906561 | n/a | *n/a* | -2.00 | 0.040 | 9.20E-05 |
| 107132211 | n/a | *n/a* | -2.57 | 0.040 | 8.86E-05 |
| 509889 | ENSBTAG00000023929 | *FOSL2* | -1.94 | 0.040 | 8.07E-05 |
| 512069 | ENSBTAG00000017010 | *FAM207A* | 1.76 | 0.040 | 8.80E-05 |
| 513116 | ENSBTAG00000013145 | *DDHD2* | -1.41 | 0.040 | 9.48E-05 |
| 534287 | ENSBTAG00000010071 | *FBXO3* | -1.39 | 0.040 | 9.36E-05 |
| 533971 | ENSBTAG00000006116 | *SUGP1* | 1.43 | 0.044 | 0.000107417 |
| 100847567 | n/a | *n/a* | 1.55 | 0.046 | 0.000115365 |
| 107131965 | n/a | *n/a* | 3.07 | 0.047 | 0.000170815 |
| 281544 | ENSBTAG00000005373 | *TPM1* | -1.51 | 0.047 | 0.000157253 |
| 281625 | ENSBTAG00000001105 | *ANXA4* | -1.36 | 0.047 | 0.000201849 |
| 282254 | ENSBTAG00000025441 | *HSPA1A* | -1.53 | 0.047 | 0.00014859 |
| 282328 | ENSBTAG00000018040 | *PSMB10* | 1.56 | 0.047 | 0.00012615 |
| 338085 | ENSBTAG00000000810 | *COPE* | 1.48 | 0.047 | 0.000204112 |
| 404189 | ENSBTAG00000004651 | *NME1* | 1.49 | 0.047 | 0.000149504 |
| 407215 | ENSBTAG00000000746 | *KAT2B* | -1.49 | 0.047 | 0.000151657 |
| 504587 | ENSBTAG00000011825 | *TRIR* | 1.37 | 0.047 | 0.000181865 |
| 504950 | ENSBTAG00000016335 | *NPM3* | 2.58 | 0.047 | 0.000224488 |
| 505763 | ENSBTAG00000018269 | *MPV17* | 1.51 | 0.047 | 0.000172674 |
| 506325 | ENSBTAG00000002578 | *ZMYND11* | -1.32 | 0.047 | 0.000212999 |
| 508130 | ENSBTAG00000002413 | *MCAT* | 1.42 | 0.047 | 0.00021169 |
| 511117 | ENSBTAG00000031553 | *SLC49A3* | 1.39 | 0.047 | 0.000208012 |
| 514407 | ENSBTAG00000005390 | *GMFG* | 1.49 | 0.047 | 0.000201113 |
| 514770 | ENSBTAG00000014848 | *NR0B2* | 2.64 | 0.047 | 0.000215059 |
| 516259 | ENSBTAG00000000421 | *EEA1* | -1.38 | 0.047 | 0.000121278 |
| 532076 | ENSBTAG00000018024 | *NR1D2* | -1.44 | 0.047 | 0.000160789 |
| 533042 | ENSBTAG00000019075 | *METAP1D* | 1.47 | 0.047 | 0.000203312 |
| 533147 | ENSBTAG00000009681 | *PPP2R3C* | 1.68 | 0.047 | 0.000223152 |
| 534358 | ENSBTAG00000009665 | *UTRN* | -1.45 | 0.047 | 0.000133239 |
| 537203 | ENSBTAG00000031890 | *DOCK1* | -1.42 | 0.047 | 0.00018382 |
| 538532 | ENSBTAG00000018727 | *GOLIM4* | -1.58 | 0.047 | 0.000176884 |
| 539625 | ENSBTAG00000020363 | *GBA3* | -1.56 | 0.047 | 0.000189722 |
| 540172 | ENSBTAG00000013440 | *RBM42* | 1.38 | 0.047 | 0.000147617 |
| 614148 | ENSBTAG00000002708 | *MRPL42* | 1.52 | 0.047 | 0.000165665 |
| 615764 | ENSBTAG00000005403 | *ADI1* | -1.61 | 0.047 | 0.000205104 |
| 616375 | ENSBTAG00000011754 | *LAMTOR2* | 1.36 | 0.047 | 0.000223245 |
| 617927 | ENSBTAG00000044126 | *SNTB1* | -1.54 | 0.047 | 0.000182364 |
| 777788 | ENSBTAG00000031544 | *DDIT3* | 1.43 | 0.047 | 0.000166509 |
| 780845 | ENSBTAG00000006245 | *WDR83* | 1.49 | 0.047 | 0.000223988 |
| 782667 | ENSBTAG00000055158 | *TP53INP1* | -1.89 | 0.047 | 0.000169501 |
| 100297914 | ENSBTAG00000048798 | *KIAA1109* | -1.41 | 0.047 | 0.000234386 |
| 286862 | ENSBTAG00000004077 | *YWHAG* | -1.50 | 0.047 | 0.000239965 |
| 532399 | ENSBTAG00000011527 | *NT5C* | 1.53 | 0.047 | 0.000237578 |
| 540228 | ENSBTAG00000039951 | *PRMT6* | 1.75 | 0.047 | 0.000231818 |
| 507199 | ENSBTAG00000003914 | *MVB12A* | 1.32 | 0.047 | 0.000244327 |
| 505884 | ENSBTAG00000015188 | *KLF6* | -2.05 | 0.049 | 0.000259987 |
| 518801 | ENSBTAG00000014574 | *CFAP43* | -3.40 | 0.049 | 0.000263432 |
| 281946 | ENSBTAG00000019472 | *NR3C1* | -1.40 | 0.052 | 0.0003033 |
| 282855 | ENSBTAG00000001936 | *PCK1* | -1.99 | 0.052 | 0.00030131 |
| 505771 | ENSBTAG00000024431 | *PEX1* | -1.50 | 0.052 | 0.000302369 |
| 507159 | ENSBTAG00000014847 | *BBOX1* | -1.51 | 0.052 | 0.000292155 |
| 535177 | ENSBTAG00000004940 | *CELF4* | -2.00 | 0.052 | 0.000303426 |
| 539633 | ENSBTAG00000019704 | *HLTF* | -1.44 | 0.052 | 0.000288356 |
| 767958 | ENSBTAG00000032557 | *TMEM258* | 1.78 | 0.052 | 0.000298589 |
| 514925 | ENSBTAG00000014517 | *KLB* | -1.61 | 0.052 | 0.000315873 |
| 520472 | ENSBTAG00000015509 | *NAMPT* | -1.46 | 0.052 | 0.000312256 |
| 534321 | ENSBTAG00000044185 | *SOX6* | -1.56 | 0.052 | 0.000311762 |
| 513052 | ENSBTAG00000011243 | *TOE1* | 1.40 | 0.053 | 0.000327508 |
| 510220 | ENSBTAG00000015900 | *CNPY3* | 1.40 | 0.054 | 0.000343056 |
| 512349 | ENSBTAG00000019972 | *TEPSIN* | 1.38 | 0.054 | 0.000341392 |
| 540771 | ENSBTAG00000003809 | *PLCD4* | -2.20 | 0.054 | 0.000342812 |
| 286797 | ENSBTAG00000000081 | *CERT1* | -1.89 | 0.056 | 0.000361913 |
| 107131208 | n/a | *n/a* | -1.62 | 0.057 | 0.000386255 |
| 280989 | ENSBTAG00000020554 | *AIF1* | 1.38 | 0.057 | 0.000384676 |
| 282196 | ENSBTAG00000008631 | *CORO1A* | 1.45 | 0.057 | 0.000392352 |
| 353160 | ENSBTAG00000017278 | *ACBD5* | -1.45 | 0.057 | 0.000373821 |
| 519017 | ENSBTAG00000000639 | *APRT* | 1.46 | 0.057 | 0.000389082 |
| 524284 | ENSBTAG00000014083 | *AMIGO1* | -1.61 | 0.057 | 0.000389316 |
| 534182 | ENSBTAG00000016707 | *FKBP9* | -1.67 | 0.057 | 0.000392431 |
| 100125314 | ENSBTAG00000046173 | *ALG12* | 1.46 | 0.061 | 0.000533112 |
| 280855 | ENSBTAG00000006161 | *MET* | -1.61 | 0.061 | 0.00046728 |
| 327671 | ENSBTAG00000011043 | *RAC2* | 1.49 | 0.061 | 0.000447829 |
| 444987 | ENSBTAG00000004769 | *NEIL2* | -1.96 | 0.061 | 0.000511315 |
| 506458 | ENSBTAG00000009107 | *CBX8* | 1.72 | 0.061 | 0.000532068 |
| 509968 | ENSBTAG00000011193 | *C1QC* | 1.33 | 0.061 | 0.000522327 |
| 512361 | ENSBTAG00000005477 | *LAPTM5* | 1.33 | 0.061 | 0.000481714 |
| 512490 | ENSBTAG00000007330 | *STXBP3* | -1.37 | 0.061 | 0.000504165 |
| 514674 | ENSBTAG00000000435 | *TARBP2* | 1.39 | 0.061 | 0.000545282 |
| 516039 | ENSBTAG00000010576 | *MAP2K3* | 1.37 | 0.061 | 0.00051188 |
| 519597 | ENSBTAG00000011383 | *SNX4* | -1.40 | 0.061 | 0.000516155 |
| 520178 | ENSBTAG00000018596 | *PTPN21* | -1.38 | 0.061 | 0.000478682 |
| 522864 | ENSBTAG00000024958 | *FAM214A* | -1.50 | 0.061 | 0.000470988 |
| 523737 | ENSBTAG00000003388 | *DPM2* | 1.46 | 0.061 | 0.000453542 |
| 531802 | ENSBTAG00000020759 | *NCLN* | 1.49 | 0.061 | 0.000437397 |
| 531942 | ENSBTAG00000009493 | *BCL3* | 1.62 | 0.061 | 0.000456306 |
| 532067 | ENSBTAG00000005501 | *COBLL1* | -1.39 | 0.061 | 0.000453256 |
| 534440 | ENSBTAG00000002112 | *INSIG2* | -1.35 | 0.061 | 0.000484203 |
| 534624 | ENSBTAG00000016779 | *CLIP1* | -1.54 | 0.061 | 0.00043142 |
| 537698 | ENSBTAG00000011723 | *GRIK5* | -3.22 | 0.061 | 0.000545009 |
| 539092 | ENSBTAG00000008285 | *OXR1* | -1.51 | 0.061 | 0.000456469 |
| 539605 | ENSBTAG00000015815 | *CFP* | 1.44 | 0.061 | 0.000533578 |
| 540002 | ENSBTAG00000020539 | *CHPF* | 1.79 | 0.061 | 0.000478868 |
| 540322 | ENSBTAG00000005278 | *WEE1* | -1.48 | 0.061 | 0.000489466 |
| 540689 | ENSBTAG00000012293 | *SPAG9* | -1.35 | 0.061 | 0.000526197 |
| 617154 | ENSBTAG00000047265 | *n/a* | 1.62 | 0.061 | 0.000540607 |
| 768063 | ENSBTAG00000003161 | *YBEY* | 1.46 | 0.061 | 0.000514164 |
| 783068 | ENSBTAG00000010888 | *VSIR* | 1.48 | 0.061 | 0.00053951 |
| 786696 | ENSBTAG00000012425 | *RTL6* | -1.54 | 0.061 | 0.000546048 |
| 526282 | ENSBTAG00000010311 | *MRM1* | 1.69 | 0.062 | 0.000562521 |
| 100126182 | ENSBTAG00000015253 | *COX19* | 1.35 | 0.063 | 0.000576313 |
| 785057 | ENSBTAG00000032617 | *FBXO44* | -1.75 | 0.064 | 0.000589373 |
| 508932 | ENSBTAG00000014907 | *IL11RA* | 1.35 | 0.065 | 0.000607337 |
| 517206 | ENSBTAG00000002121 | *CDK2AP2* | 1.82 | 0.065 | 0.000607518 |
| 508170 | ENSBTAG00000035247 | *MCEE* | 1.34 | 0.065 | 0.000618598 |
| 518907 | ENSBTAG00000018284 | *KBTBD11* | -1.66 | 0.065 | 0.000617689 |
| 281883 | ENSBTAG00000008294 | *KCNJ2* | -3.12 | 0.066 | 0.000629937 |
| 618793 | ENSBTAG00000004879 | *FOXO4* | -1.45 | 0.066 | 0.000635543 |
| 327692 | ENSBTAG00000018469 | *ALDH6A1* | -1.63 | 0.067 | 0.000655695 |
| 101906072 | n/a | *n/a* | -1.48 | 0.069 | 0.000693531 |
| 516952 | ENSBTAG00000021158 | *SATB1* | -1.60 | 0.069 | 0.000679918 |
| 517987 | ENSBTAG00000013339 | *NEBL* | -1.70 | 0.069 | 0.000694926 |
| 521261 | ENSBTAG00000017492 | *PCMTD1* | -1.48 | 0.069 | 0.0006825 |
| 523752 | ENSBTAG00000019759 | *IDE* | -1.79 | 0.069 | 0.000698931 |
| 538404 | ENSBTAG00000019585 | *MYOM1* | -4.08 | 0.069 | 0.000698774 |
| 100296268 | n/a | *n/a* | 1.45 | 0.069 | 0.000805217 |
| 100296470 | ENSBTAG00000054199 | *CHCHD8* | 1.48 | 0.069 | 0.000809738 |
| 100336629 | ENSBTAG00000039307 | *YAP1* | -1.32 | 0.069 | 0.000727654 |
| 107132947 | n/a | *n/a* | 1.71 | 0.069 | 0.0008211 |
| 281206 | ENSBTAG00000011960 | *GOT1* | 2.00 | 0.069 | 0.000740915 |
| 281209 | ENSBTAG00000054195 | *GPX1* | 1.56 | 0.069 | 0.000740958 |
| 281217 | ENSBTAG00000014775 | *GUK1* | 1.29 | 0.069 | 0.000742945 |
| 507431 | ENSBTAG00000016313 | *ECH1* | 1.37 | 0.069 | 0.000784596 |
| 507858 | ENSBTAG00000010365 | *SQOR* | 1.44 | 0.069 | 0.000768008 |
| 510498 | ENSBTAG00000043981 | *SBF2* | -1.44 | 0.069 | 0.000775824 |
| 512534 | ENSBTAG00000008840 | *HM13* | 1.60 | 0.069 | 0.000842914 |
| 514906 | ENSBTAG00000017095 | *DENND4A* | -1.31 | 0.069 | 0.000728652 |
| 515660 | ENSBTAG00000016561 | *NUBP2* | 1.45 | 0.069 | 0.000775769 |
| 519526 | ENSBTAG00000000201 | *LTN1* | -1.72 | 0.069 | 0.000804471 |
| 524427 | ENSBTAG00000020465 | *FERMT1* | -1.96 | 0.069 | 0.000748359 |
| 525154 | ENSBTAG00000019246 | *SC5D* | -1.68 | 0.069 | 0.000748265 |
| 526320 | ENSBTAG00000009483 | *POLR2G* | 1.38 | 0.069 | 0.000753515 |
| 526872 | ENSBTAG00000000517 | *ANKRD29* | -2.31 | 0.069 | 0.000762801 |
| 529335 | ENSBTAG00000019855 | *MAP3K15* | -1.34 | 0.069 | 0.000824069 |
| 534842 | ENSBTAG00000010462 | *ROBO2* | -1.79 | 0.069 | 0.000846619 |
| 537464 | ENSBTAG00000019877 | *DOCK7* | -1.44 | 0.069 | 0.000787061 |
| 537894 | ENSBTAG00000003354 | *SMCHD1* | -1.32 | 0.069 | 0.000830269 |
| 540532 | ENSBTAG00000009210 | *ZBTB44* | -1.33 | 0.069 | 0.000822303 |
| 540610 | ENSBTAG00000012654 | *CHCHD1* | 1.41 | 0.069 | 0.0008507 |
| 613442 | ENSBTAG00000015479 | *ZFPL1* | 1.48 | 0.069 | 0.000849412 |
| 614576 | ENSBTAG00000019177 | *BIN1* | 1.52 | 0.069 | 0.00084195 |
| 616621 | ENSBTAG00000001834 | *PNKP* | 1.32 | 0.069 | 0.000801584 |
| 616814 | ENSBTAG00000030933 | *ZNF576* | 1.47 | 0.069 | 0.000820502 |
| 617891 | ENSBTAG00000023333 | *LENG1* | 1.41 | 0.069 | 0.000812392 |
| 618791 | ENSBTAG00000025788 | *MPV17L2* | 1.74 | 0.069 | 0.00081879 |
| 101907541 | ENSBTAG00000033662 | *NCKAP1* | -1.39 | 0.069 | 0.000858494 |
| 510824 | ENSBTAG00000006936 | *MRPS18B* | 1.50 | 0.070 | 0.000867956 |
| 281122 | ENSBTAG00000048246 | *DPP4* | -1.40 | 0.070 | 0.00092861 |
| 507115 | ENSBTAG00000005470 | *BIN3* | 1.89 | 0.070 | 0.000913364 |
| 507939 | ENSBTAG00000005191 | *RPN1* | 1.58 | 0.070 | 0.000939685 |
| 508399 | ENSBTAG00000005842 | *ABCC10* | 1.40 | 0.070 | 0.000891498 |
| 509087 | ENSBTAG00000046875 | *AP5B1* | 1.42 | 0.070 | 0.000937457 |
| 511011 | ENSBTAG00000003609 | *PBDC1* | 1.30 | 0.070 | 0.00093283 |
| 513405 | ENSBTAG00000003225 | *IMP4* | 1.44 | 0.070 | 0.000892993 |
| 530171 | n/a | *n/a* | -2.41 | 0.070 | 0.000927483 |
| 540724 | ENSBTAG00000033095 | *WDR70* | 1.41 | 0.070 | 0.000909066 |
| 614728 | ENSBTAG00000046746 | *COASY* | 1.42 | 0.070 | 0.000907971 |
| 615863 | ENSBTAG00000007388 | *ZC3H12D* | 1.61 | 0.070 | 0.000927242 |
| 616423 | ENSBTAG00000010465 | *C29H11orf98* | 1.48 | 0.070 | 0.000932844 |
| 100138004 | ENSBTAG00000048013 | *n/a* | -1.95 | 0.071 | 0.000979837 |
| 104976274 | n/a | *n/a* | 1.42 | 0.071 | 0.001068015 |
| 281048 | ENSBTAG00000015032 | *CD14* | 1.63 | 0.071 | 0.001048719 |
| 281110 | ENSBTAG00000012012 | *CYB5A* | -1.47 | 0.071 | 0.001016215 |
| 282472 | ENSBTAG00000003282 | *SLCO1A2* | -1.70 | 0.071 | 0.001063387 |
| 287022 | ENSBTAG00000000236 | *YWHAZ* | -1.75 | 0.071 | 0.000972154 |
| 505276 | ENSBTAG00000015187 | *DDX41* | 1.27 | 0.071 | 0.001060785 |
| 506534 | ENSBTAG00000004374 | *CNPY2* | 1.54 | 0.071 | 0.001027043 |
| 507021 | ENSBTAG00000016529 | *SLC25A30* | -1.88 | 0.071 | 0.001055926 |
| 508009 | ENSBTAG00000004423 | *ARHGAP42* | -1.53 | 0.071 | 0.000960601 |
| 508132 | ENSBTAG00000014668 | *NAGK* | 1.38 | 0.071 | 0.001003215 |
| 508386 | ENSBTAG00000011238 | *CD48* | 1.42 | 0.071 | 0.001035619 |
| 508733 | ENSBTAG00000006244 | *WDR83OS* | 1.42 | 0.071 | 0.001019054 |
| 511077 | ENSBTAG00000008409 | *MYC* | -2.20 | 0.071 | 0.000984024 |
| 515620 | ENSBTAG00000011189 | *TJAP1* | 1.44 | 0.071 | 0.001011299 |
| 520625 | ENSBTAG00000005675 | *SLC44A4* | 4.46 | 0.071 | 0.001042953 |
| 524909 | ENSBTAG00000014467 | *n/a* | -1.67 | 0.071 | 0.001065439 |
| 527669 | ENSBTAG00000018808 | *SLC25A35* | 1.69 | 0.071 | 0.001017225 |
| 532489 | ENSBTAG00000018394 | *SDR42E1* | -1.41 | 0.071 | 0.001028198 |
| 533642 | ENSBTAG00000007331 | *PLOD2* | -1.57 | 0.071 | 0.000993062 |
| 535372 | ENSBTAG00000014601 | *SGCB* | -1.34 | 0.071 | 0.00102967 |
| 540661 | ENSBTAG00000013602 | *BRWD1* | -1.39 | 0.071 | 0.001049053 |
| 541166 | ENSBTAG00000014915 | *ETV5* | 1.92 | 0.071 | 0.00104432 |
| 613998 | ENSBTAG00000008340 | *ATOX1* | 1.45 | 0.071 | 0.000967985 |
| 785291 | ENSBTAG00000020378 | *DLG1* | -1.29 | 0.071 | 0.00104925 |
| 539275 | ENSBTAG00000002287 | *CHD9* | -1.35 | 0.072 | 0.001087033 |
| 614624 | ENSBTAG00000005652 | *ALG3* | 1.43 | 0.072 | 0.001102249 |
| 614538 | ENSBTAG00000019077 | *DAD1* | 1.49 | 0.073 | 0.001122426 |
| 516731 | ENSBTAG00000021140 | *RMND5A* | -1.41 | 0.073 | 0.001127739 |
| 100140713 | ENSBTAG00000019721 | *REXO4* | 1.41 | 0.074 | 0.001231284 |
| 100848337 | ENSBTAG00000046010 | *BHLHA15* | 2.02 | 0.074 | 0.001193608 |
| 327679 | ENSBTAG00000014239 | *CCNB1* | -3.59 | 0.074 | 0.001177543 |
| 507833 | ENSBTAG00000008646 | *TRMT112* | 1.36 | 0.074 | 0.001219994 |
| 508236 | ENSBTAG00000021853 | *IP6K2* | -1.67 | 0.074 | 0.001228032 |
| 510453 | ENSBTAG00000038498 | *ZBTB39* | -1.39 | 0.074 | 0.001200931 |
| 511446 | ENSBTAG00000021884 | *CXXC1* | 1.26 | 0.074 | 0.001195965 |
| 514798 | ENSBTAG00000016795 | *RUFY3* | -1.44 | 0.074 | 0.001188696 |
| 524608 | ENSBTAG00000014889 | *DCBLD2* | -1.71 | 0.074 | 0.001151465 |
| 527436 | ENSBTAG00000009800 | *MSI1* | -4.89 | 0.074 | 0.001175089 |
| 527592 | ENSBTAG00000006307 | *HSD17B11* | -1.39 | 0.074 | 0.001225271 |
| 533726 | ENSBTAG00000004688 | *DHCR24* | -1.75 | 0.074 | 0.001210942 |
| 535125 | ENSBTAG00000047834 | *EFR3A* | -1.54 | 0.074 | 0.001225719 |
| 536240 | ENSBTAG00000032477 | *HECTD1* | -1.34 | 0.074 | 0.001188426 |
| 574400 | ENSBTAG00000009795 | *SF3B5* | 1.40 | 0.074 | 0.001228946 |
| 614852 | ENSBTAG00000005030 | *INO80E* | 1.39 | 0.074 | 0.00120989 |
| 616487 | ENSBTAG00000009121 | *STAG2* | -1.40 | 0.074 | 0.001216532 |
| 101906096 | ENSBTAG00000049864 | *PPP1R9B* | 1.28 | 0.074 | 0.001260741 |
| 517995 | ENSBTAG00000045966 | *GPRIN3* | -1.32 | 0.074 | 0.00125686 |
| 519541 | ENSBTAG00000055051 | *RNF125* | -1.69 | 0.074 | 0.001256409 |
| 525256 | ENSBTAG00000007080 | *PREB* | 1.57 | 0.074 | 0.001249654 |
| 536267 | ENSBTAG00000007963 | *KIDINS220* | -1.35 | 0.074 | 0.001249053 |
| 523338 | ENSBTAG00000003395 | *ZRANB1* | -1.32 | 0.074 | 0.001268877 |
| 618702 | ENSBTAG00000023343 | *RPL28* | 1.35 | 0.074 | 0.001272346 |
| 100138303 | ENSBTAG00000008585 | *ARHGEF10* | -1.47 | 0.075 | 0.001286243 |
| 282661 | ENSBTAG00000003585 | *CD47* | -1.33 | 0.075 | 0.001318921 |
| 509559 | ENSBTAG00000047793 | *TSSC4* | 1.57 | 0.075 | 0.001300585 |
| 512261 | ENSBTAG00000007065 | *TRAPPC6A* | 1.39 | 0.075 | 0.001317815 |
| 529462 | n/a | *n/a* | -1.94 | 0.075 | 0.001313969 |
| 530484 | ENSBTAG00000010630 | *ABHD18* | -1.32 | 0.075 | 0.001296346 |
| 616937 | ENSBTAG00000047550 | *KIAA2026* | -1.49 | 0.075 | 0.001312704 |
| 506362 | ENSBTAG00000032905 | *RMDN2* | -1.51 | 0.075 | 0.001356256 |
| 510866 | ENSBTAG00000007498 | *ZGPAT* | 1.33 | 0.075 | 0.001351898 |
| 511239 | ENSBTAG00000018556 | *MGC137211* | -1.55 | 0.075 | 0.001337775 |
| 516291 | ENSBTAG00000002705 | *REC8* | 3.88 | 0.075 | 0.001335665 |
| 539359 | ENSBTAG00000010693 | *LMO7* | -1.40 | 0.075 | 0.001349703 |
| 539458 | ENSBTAG00000015887 | *FOXJ3* | -1.46 | 0.075 | 0.001355139 |
| 525659 | ENSBTAG00000007842 | *GCLM* | -1.33 | 0.075 | 0.001369505 |
| 616275 | ENSBTAG00000001937 | *ANAPC11* | 1.27 | 0.075 | 0.001368108 |
| 282013 | ENSBTAG00000003039 | *PSMB8* | 1.54 | 0.075 | 0.001387596 |
| 504842 | ENSBTAG00000011954 | *SEC11C* | 1.75 | 0.075 | 0.001387726 |
| 508115 | ENSBTAG00000014505 | *NGRN* | 1.28 | 0.075 | 0.001384274 |
| 518976 | ENSBTAG00000021678 | *SLC31A1* | -1.32 | 0.075 | 0.001392735 |
| 514443 | ENSBTAG00000000694 | *TAF10* | 1.31 | 0.076 | 0.001412015 |
| 533086 | ENSBTAG00000011720 | *HSD17B6* | -1.43 | 0.076 | 0.00140924 |
| 614007 | ENSBTAG00000010587 | *SH3BGRL* | -1.34 | 0.076 | 0.00141842 |
| 514979 | ENSBTAG00000023635 | *CLEC6A* | 2.30 | 0.076 | 0.001442621 |
| 532555 | ENSBTAG00000020357 | *GOLPH3L* | 1.41 | 0.076 | 0.001443066 |
| 538598 | n/a | *n/a* | -1.46 | 0.076 | 0.001450967 |
| 614075 | ENSBTAG00000012747 | *FAM3A* | 1.36 | 0.076 | 0.001453196 |
| 101904121 | n/a | *n/a* | -1.92 | 0.077 | 0.001478549 |
| 513526 | ENSBTAG00000019804 | *SNRNP25* | 1.65 | 0.077 | 0.001476491 |
| 521742 | ENSBTAG00000015151 | *FOXN2* | -2.21 | 0.077 | 0.001470571 |
| 537799 | ENSBTAG00000010694 | *BICC1* | -1.62 | 0.077 | 0.001486428 |
| 107131209 | ENSBTAG00000006864 | *n/a* | 1.44 | 0.077 | 0.001496274 |
| 524648 | ENSBTAG00000007634 | *HOOK3* | -1.44 | 0.077 | 0.001498511 |
| 506566 | ENSBTAG00000011850 | *ALG5* | 1.55 | 0.077 | 0.001511345 |
| 537649 | ENSBTAG00000016691 | *LACC1* | -1.56 | 0.079 | 0.00154528 |
| 518781 | ENSBTAG00000011567 | *VPS18* | 1.34 | 0.079 | 0.001556851 |
| 513458 | ENSBTAG00000007365 | *NEIL1* | 1.47 | 0.079 | 0.001567701 |
| 107131338 | n/a | *n/a* | -1.88 | 0.080 | 0.001613222 |
| 281158 | ENSBTAG00000006393 | *FECH* | -1.33 | 0.080 | 0.001621965 |
| 505642 | ENSBTAG00000003989 | *GSTO1* | 2.85 | 0.080 | 0.001614901 |
| 510583 | ENSBTAG00000012794 | *PAH* | -1.49 | 0.080 | 0.001606069 |
| 513579 | ENSBTAG00000001657 | *PICALM* | -1.26 | 0.080 | 0.001608201 |
| 515416 | ENSBTAG00000038186 | *GLRX* | 1.47 | 0.080 | 0.001598204 |
| 518313 | ENSBTAG00000004023 | *KIAA1324L* | -2.77 | 0.080 | 0.001620116 |
| 537603 | ENSBTAG00000021107 | *STXBP5* | -1.42 | 0.080 | 0.001603108 |
| 528813 | ENSBTAG00000017674 | *SCNN1D* | -3.23 | 0.080 | 0.001637186 |
| 615199 | ENSBTAG00000007737 | *UBA52* | 1.35 | 0.081 | 0.00164959 |
| 527458 | ENSBTAG00000014114 | *KLHL26* | 2.58 | 0.081 | 0.001663681 |
| 533499 | ENSBTAG00000012704 | *MMS19* | 1.26 | 0.081 | 0.001657865 |
| 281551 | ENSBTAG00000016912 | *TSFM* | 1.40 | 0.081 | 0.001768752 |
| 282330 | ENSBTAG00000019230 | *PTGER3* | -1.91 | 0.081 | 0.001782506 |
| 504658 | ENSBTAG00000001619 | *SERINC5* | -1.31 | 0.081 | 0.001754389 |
| 504889 | ENSBTAG00000002995 | *GMPPA* | 1.80 | 0.081 | 0.001703395 |
| 507125 | ENSBTAG00000017386 | *C3H1orf50* | 1.36 | 0.081 | 0.001790068 |
| 508105 | ENSBTAG00000000436 | *TNFAIP3* | -1.61 | 0.081 | 0.001733611 |
| 508954 | ENSBTAG00000025496 | *SORD* | -1.42 | 0.081 | 0.001800809 |
| 509275 | n/a | *n/a* | 2.24 | 0.081 | 0.001700478 |
| 511548 | ENSBTAG00000007759 | *ABCB8* | 1.43 | 0.081 | 0.001701143 |
| 513983 | ENSBTAG00000005573 | *SCAND1* | 1.70 | 0.081 | 0.001727359 |
| 513996 | ENSBTAG00000030174 | *ACOX1* | -1.53 | 0.081 | 0.001760683 |
| 518025 | ENSBTAG00000012079 | *MYLK4* | -1.96 | 0.081 | 0.001799487 |
| 521237 | ENSBTAG00000020613 | *STK16* | 1.23 | 0.081 | 0.001795375 |
| 523153 | ENSBTAG00000027201 | *RAPH1* | -1.40 | 0.081 | 0.001735629 |
| 528138 | ENSBTAG00000000501 | *KLHL13* | -1.61 | 0.081 | 0.001710687 |
| 533126 | ENSBTAG00000002554 | *TCAF1* | -1.33 | 0.081 | 0.001797992 |
| 536815 | ENSBTAG00000009851 | *ROBO1* | -1.50 | 0.081 | 0.001691168 |
| 537412 | ENSBTAG00000009988 | *SMARCB1* | 1.50 | 0.081 | 0.001757139 |
| 540247 | ENSBTAG00000001104 | *CMTM4* | -1.31 | 0.081 | 0.001697555 |
| 616030 | ENSBTAG00000004913 | *RNF167* | 1.32 | 0.081 | 0.001715479 |
| 617407 | ENSBTAG00000010910 | *COPS9* | 1.38 | 0.081 | 0.001767834 |
| 617720 | ENSBTAG00000027728 | *NUDT12* | -1.33 | 0.081 | 0.001769547 |
| 788473 | ENSBTAG00000001663 | *PDF* | 1.51 | 0.081 | 0.001676744 |
| 505968 | ENSBTAG00000024240 | *ACADM* | -1.68 | 0.081 | 0.001813262 |
| 616324 | n/a | *n/a* | 1.38 | 0.081 | 0.001811558 |
| 508431 | ENSBTAG00000000308 | *ITCH* | -1.35 | 0.081 | 0.001823492 |
| 510313 | ENSBTAG00000016855 | *BUD13* | 1.32 | 0.081 | 0.001826972 |
| 100336649 | ENSBTAG00000049685 | *PTDSS2* | 1.41 | 0.081 | 0.001866855 |
| 107132838 | n/a | *n/a* | -1.66 | 0.081 | 0.001892176 |
| 281626 | ENSBTAG00000021746 | *ANXA5* | -1.45 | 0.081 | 0.001858904 |
| 281827 | ENSBTAG00000007807 | *HSPA2* | -1.59 | 0.081 | 0.001883261 |
| 520463 | ENSBTAG00000003083 | *SLC50A1* | 1.82 | 0.081 | 0.001844841 |
| 533901 | ENSBTAG00000009279 | *TNKS2* | -1.39 | 0.081 | 0.001868869 |
| 534652 | ENSBTAG00000016612 | *NEK9* | -1.37 | 0.081 | 0.001857491 |
| 535702 | ENSBTAG00000002129 | *KLF5* | -2.18 | 0.081 | 0.001891093 |
| 537932 | ENSBTAG00000020296 | *UBR3* | -1.44 | 0.081 | 0.001865055 |
| 539182 | ENSBTAG00000003020 | *SSC4D* | 1.90 | 0.081 | 0.001861528 |
| 615952 | ENSBTAG00000017689 | *BRMS1* | 1.27 | 0.081 | 0.001885641 |
| 617344 | ENSBTAG00000013996 | *SH3BP2* | 1.36 | 0.081 | 0.00190335 |
| 532189 | ENSBTAG00000012371 | *CPD* | -1.92 | 0.081 | 0.001911441 |
| 507031 | ENSBTAG00000017139 | *STX7* | -1.27 | 0.082 | 0.001929058 |
| 505315 | ENSBTAG00000016275 | *AMDHD1* | -1.86 | 0.082 | 0.001971828 |
| 511498 | ENSBTAG00000023939 | *n/a* | -1.34 | 0.082 | 0.001961386 |
| 520277 | ENSBTAG00000014583 | *CALM3* | 1.28 | 0.082 | 0.001956234 |
| 521822 | ENSBTAG00000015505 | *FADS2* | -4.02 | 0.082 | 0.001942338 |
| 527048 | n/a | *n/a* | -1.63 | 0.082 | 0.001968396 |
| 531877 | ENSBTAG00000003825 | *PTPN12* | -1.33 | 0.082 | 0.001970974 |
| 783379 | ENSBTAG00000033735 | *WASHC4* | -1.30 | 0.082 | 0.001968659 |
| 281615 | ENSBTAG00000008103 | *ALDH1A1* | -1.55 | 0.082 | 0.001991692 |
| 618238 | ENSBTAG00000054278 | *M-SAA3.2* | 5.70 | 0.082 | 0.001991699 |
| 512792 | ENSBTAG00000014353 | *SRP14* | 1.36 | 0.083 | 0.0020053 |
| 518623 | ENSBTAG00000038058 | *n/a* | -3.60 | 0.083 | 0.002011291 |
| 280961 | ENSBTAG00000007730 | *ZFX* | -1.42 | 0.083 | 0.002021447 |
| 281061 | ENSBTAG00000010109 | *CDK1* | -3.15 | 0.083 | 0.00204834 |
| 282041 | ENSBTAG00000005847 | *ROCK2* | -1.64 | 0.083 | 0.002061873 |
| 504401 | ENSBTAG00000002974 | *FMO2* | -1.68 | 0.083 | 0.002031982 |
| 506766 | ENSBTAG00000001598 | *MAP7* | -1.24 | 0.083 | 0.002060297 |
| 516473 | ENSBTAG00000014643 | *EEF1D* | 1.36 | 0.083 | 0.002051706 |
| 525655 | ENSBTAG00000017179 | *USP12* | -1.30 | 0.083 | 0.002050861 |
| 529186 | ENSBTAG00000018846 | *CLN5* | -1.31 | 0.083 | 0.002043573 |
| 516555 | ENSBTAG00000009655 | *TNS3* | -1.41 | 0.083 | 0.002075714 |
| 781806 | ENSBTAG00000018164 | *FNDC4* | 2.01 | 0.083 | 0.002071582 |
| 513967 | ENSBTAG00000039161 | *ALDH3A2* | -1.42 | 0.083 | 0.002084941 |
| 100337373 | ENSBTAG00000012101 | *SYMPK* | 1.29 | 0.083 | 0.002096919 |
| 507154 | ENSBTAG00000001419 | *MRPL4* | 1.24 | 0.083 | 0.002112295 |
| 534329 | n/a | *n/a* | -1.91 | 0.083 | 0.002104223 |
| 536075 | ENSBTAG00000010399 | *TXNDC16* | -2.06 | 0.083 | 0.002115711 |
| 540389 | ENSBTAG00000010617 | *KRTCAP2* | 1.37 | 0.083 | 0.002107214 |
| 107131517 | n/a | *n/a* | 1.47 | 0.083 | 0.002153493 |
| 508487 | ENSBTAG00000019161 | *NPRL2* | 1.38 | 0.083 | 0.002141001 |
| 509914 | ENSBTAG00000010723 | *PFDN6* | 1.27 | 0.083 | 0.002152542 |
| 521401 | ENSBTAG00000001022 | *AMDHD2* | 1.34 | 0.083 | 0.002142723 |
| 522283 | ENSBTAG00000010559 | *DHRS7B* | 1.36 | 0.083 | 0.002134277 |
| 615175 | ENSBTAG00000012152 | *NONO* | -1.41 | 0.083 | 0.002153768 |
| 104974144 | n/a | *n/a* | 1.92 | 0.084 | 0.002187811 |
| 282641 | ENSBTAG00000009552 | *ATP2B1* | -1.27 | 0.084 | 0.002209781 |
| 353350 | ENSBTAG00000005862 | *SMC4* | -1.50 | 0.084 | 0.002200206 |
| 509255 | ENSBTAG00000014202 | *CDK5RAP3* | 1.28 | 0.084 | 0.002206132 |
| 518356 | ENSBTAG00000009019 | *SH3PXD2B* | -1.34 | 0.084 | 0.002192685 |
| 531336 | ENSBTAG00000013468 | *AHNAK* | -1.61 | 0.084 | 0.002176553 |
| 533161 | ENSBTAG00000015280 | *KIF2C* | -3.78 | 0.084 | 0.002180878 |
| 615890 | ENSBTAG00000003365 | *SMG8* | -2.40 | 0.084 | 0.002216614 |
| 513924 | ENSBTAG00000021144 | *PSTPIP1* | 1.55 | 0.084 | 0.002231381 |
| 538455 | ENSBTAG00000007660 | *ZNF414* | 1.44 | 0.084 | 0.002248736 |
| 517383 | ENSBTAG00000044035 | *GPRIN1* | -2.42 | 0.085 | 0.002266201 |
| 781665 | ENSBTAG00000046841 | *IRF2BP2* | -1.28 | 0.085 | 0.002265009 |
| 510698 | ENSBTAG00000007390 | *VAT1* | -1.43 | 0.085 | 0.002279118 |
| 613523 | ENSBTAG00000013070 | *EAF2* | 2.32 | 0.085 | 0.002280791 |
| 613592 | ENSBTAG00000025564 | *RPL36AL* | 1.38 | 0.085 | 0.002289388 |
| 786586 | n/a | *n/a* | 1.46 | 0.085 | 0.002297154 |
| 100135053 | ENSBTAG00000021222 | *ZNF771* | 1.40 | 0.085 | 0.002306548 |
| 505472 | ENSBTAG00000011494 | *PYGL* | -1.32 | 0.085 | 0.002324437 |
| 282874 | ENSBTAG00000004287 | *EVC* | -1.86 | 0.086 | 0.002359585 |
| 504742 | ENSBTAG00000015113 | *CITED4* | 1.53 | 0.086 | 0.002373459 |
| 508208 | ENSBTAG00000014406 | *LATS2* | -1.33 | 0.086 | 0.002373706 |
| 509463 | ENSBTAG00000018375 | *ATCAY* | 2.07 | 0.086 | 0.002375436 |
| 767855 | ENSBTAG00000010504 | *TBRG4* | 1.38 | 0.086 | 0.002366212 |
| 100847143 | n/a | *n/a* | 1.58 | 0.086 | 0.002422904 |
| 504647 | ENSBTAG00000012141 | *RAD9A* | 1.39 | 0.086 | 0.002446867 |
| 506812 | ENSBTAG00000021999 | *CPT1A* | -1.46 | 0.086 | 0.002451344 |
| 509816 | ENSBTAG00000019831 | *MRPS11* | 1.35 | 0.086 | 0.002453959 |
| 511736 | ENSBTAG00000006542 | *RABEP2* | 1.27 | 0.086 | 0.002419715 |
| 526134 | ENSBTAG00000001706 | *SLC9A2* | -3.90 | 0.086 | 0.002415592 |
| 530071 | ENSBTAG00000006357 | *CRIP3* | 2.97 | 0.086 | 0.002391141 |
| 530653 | ENSBTAG00000025760 | *n/a* | -1.36 | 0.086 | 0.002457827 |
| 537451 | ENSBTAG00000013333 | *GYS2* | -1.39 | 0.086 | 0.00241308 |
| 538392 | ENSBTAG00000022777 | *CDC42BPA* | -1.50 | 0.086 | 0.002459734 |
| 539532 | ENSBTAG00000010052 | *EXD2* | -2.20 | 0.086 | 0.002415317 |
| 614302 | ENSBTAG00000010672 | *PCCA* | -1.30 | 0.086 | 0.002438393 |
| 618423 | ENSBTAG00000017631 | *GFER* | 1.39 | 0.086 | 0.00240267 |
| 281062 | ENSBTAG00000021190 | *CDH2* | -1.42 | 0.086 | 0.002483413 |
| 507631 | ENSBTAG00000007003 | *TYMS* | -1.50 | 0.086 | 0.002515302 |
| 513428 | ENSBTAG00000004590 | *PPIH* | 1.32 | 0.086 | 0.002486064 |
| 529689 | ENSBTAG00000016484 | *ATP11C* | -1.50 | 0.086 | 0.002503352 |
| 533842 | ENSBTAG00000002798 | *NABP2* | 1.34 | 0.086 | 0.002497091 |
| 535981 | ENSBTAG00000014712 | *CLPP* | 1.36 | 0.086 | 0.002509952 |
| 618745 | ENSBTAG00000045989 | *CDC42EP5* | -1.68 | 0.086 | 0.0024867 |
| 767889 | ENSBTAG00000006852 | *ACYP2* | -1.76 | 0.086 | 0.002495921 |
| 506043 | ENSBTAG00000015828 | *FKBP11* | 2.04 | 0.087 | 0.002529961 |
| 514618 | ENSBTAG00000012784 | *RACGAP1* | -1.90 | 0.087 | 0.002539572 |
| 789082 | ENSBTAG00000025452 | *SF3A2* | 1.35 | 0.087 | 0.002543196 |
| 414732 | ENSBTAG00000005586 | *GATM* | -1.97 | 0.087 | 0.002590974 |
| 504746 | ENSBTAG00000023814 | *ECT2* | -2.82 | 0.087 | 0.002639118 |
| 505159 | ENSBTAG00000010318 | *FDX2* | 1.34 | 0.087 | 0.002576648 |
| 508832 | ENSBTAG00000010447 | *LSP1* | 1.36 | 0.087 | 0.002645289 |
| 513653 | n/a | *n/a* | -3.17 | 0.087 | 0.002610075 |
| 516919 | ENSBTAG00000002377 | *PSMB2* | 1.39 | 0.087 | 0.002641879 |
| 534246 | ENSBTAG00000013277 | *CCDC22* | 1.30 | 0.087 | 0.002615427 |
| 534994 | ENSBTAG00000023976 | *ARHGAP25* | 1.40 | 0.087 | 0.002585197 |
| 536426 | ENSBTAG00000020468 | *MICU3* | -1.41 | 0.087 | 0.002597226 |
| 537791 | ENSBTAG00000010818 | *AEBP2* | -1.34 | 0.087 | 0.002616461 |
| 540428 | ENSBTAG00000005446 | *POLR2J* | 1.39 | 0.087 | 0.002584329 |
| 615443 | ENSBTAG00000014422 | *MTMR10* | -1.58 | 0.087 | 0.002642581 |
| 617005 | ENSBTAG00000022009 | *MRPL38* | 1.24 | 0.087 | 0.002580249 |
| 618444 | ENSBTAG00000023787 | *TOR4A* | 1.49 | 0.087 | 0.002635784 |
| 407127 | ENSBTAG00000006420 | *BMPR2* | -1.31 | 0.087 | 0.002659375 |
| 507100 | ENSBTAG00000021709 | *SPI1* | 1.38 | 0.087 | 0.002668868 |
| 516932 | ENSBTAG00000018834 | *PPP1R37* | 1.31 | 0.087 | 0.002672806 |
| 280820 | ENSBTAG00000001265 | *HRG* | -2.27 | 0.088 | 0.002728789 |
| 282066 | ENSBTAG00000054085 | *SELENOP* | -1.41 | 0.088 | 0.002747408 |
| 282390 | ENSBTAG00000007338 | *TYROBP* | 1.44 | 0.088 | 0.002716318 |
| 505009 | ENSBTAG00000007623 | *CPTP* | 1.29 | 0.088 | 0.002694149 |
| 507285 | ENSBTAG00000020931 | *CHN2* | -1.49 | 0.088 | 0.002734317 |
| 509108 | ENSBTAG00000017463 | *RPS19BP1* | 1.42 | 0.088 | 0.002745537 |
| 514441 | ENSBTAG00000016995 | *HDHD5* | 1.40 | 0.088 | 0.002699124 |
| 536435 | ENSBTAG00000039855 | *SUMF1* | -1.28 | 0.088 | 0.002743916 |
| 536607 | ENSBTAG00000009696 | *ACTN2* | 2.21 | 0.088 | 0.002722342 |
| 615106 | ENSBTAG00000007000 | *ENOSF1* | -1.53 | 0.088 | 0.002712311 |
| 538054 | ENSBTAG00000021188 | *MRPL16* | 1.24 | 0.088 | 0.002769583 |
| 101906884 | n/a | *n/a* | -1.77 | 0.089 | 0.002788604 |
| 513376 | ENSBTAG00000010389 | *STBD1* | -1.43 | 0.089 | 0.002794815 |
| 534894 | ENSBTAG00000018498 | *PQBP1* | 1.36 | 0.089 | 0.002804932 |
| 511306 | ENSBTAG00000012596 | *POP1* | 1.46 | 0.089 | 0.002825925 |
| 613525 | n/a | *n/a* | 1.41 | 0.089 | 0.002823863 |
| 786890 | ENSBTAG00000047772 | *FBXO48* | -2.38 | 0.089 | 0.002821252 |
| 512499 | ENSBTAG00000012235 | *SHARPIN* | 1.42 | 0.089 | 0.002833074 |
| 282846 | ENSBTAG00000020535 | *PYCARD* | 1.49 | 0.089 | 0.002846678 |
| 522946 | ENSBTAG00000019497 | *ILDR2* | -1.98 | 0.089 | 0.002840772 |
| 508412 | ENSBTAG00000034992 | *MCTS1* | 1.26 | 0.089 | 0.002860619 |
| 281570 | ENSBTAG00000008692 | *UQCR11* | 1.47 | 0.089 | 0.002881109 |
| 506353 | ENSBTAG00000019278 | *KNTC1* | -1.96 | 0.089 | 0.00287873 |
| 504781 | ENSBTAG00000009899 | *CHID1* | 1.39 | 0.090 | 0.002892729 |
| 617215 | ENSBTAG00000011658 | *PTPN18* | 1.33 | 0.090 | 0.002899714 |
| 101903772 | ENSBTAG00000038845 | *TYSND1* | 1.36 | 0.090 | 0.002936049 |
| 104968435 | n/a | *n/a* | 1.36 | 0.090 | 0.002979738 |
| 281103 | ENSBTAG00000006367 | *CCN2* | -1.57 | 0.090 | 0.002923008 |
| 509802 | ENSBTAG00000009077 | *SNRPA* | 1.43 | 0.090 | 0.002979324 |
| 510380 | ENSBTAG00000023549 | *CYP2C19* | -2.10 | 0.090 | 0.002912816 |
| 515395 | ENSBTAG00000038992 | *STK11IP* | 1.39 | 0.090 | 0.002964603 |
| 515615 | ENSBTAG00000021472 | *ZC3H3* | 1.48 | 0.090 | 0.002924468 |
| 534778 | ENSBTAG00000054881 | *RNLS* | 1.36 | 0.090 | 0.002958193 |
| 539139 | ENSBTAG00000016229 | *KLF9* | -2.07 | 0.090 | 0.002967133 |
| 539217 | ENSBTAG00000008772 | *SMC2* | -1.67 | 0.090 | 0.002959251 |
| 617435 | ENSBTAG00000011196 | *C1QB* | 1.31 | 0.090 | 0.002938348 |
| 785540 | ENSBTAG00000049496 | *n/a* | -2.65 | 0.090 | 0.002948944 |
| 512318 | ENSBTAG00000008457 | *SLU7* | -1.46 | 0.090 | 0.002988504 |
| 281992 | ENSBTAG00000008063 | *PPARA* | -1.34 | 0.090 | 0.003021951 |
| 506264 | ENSBTAG00000009020 | *CRIM1* | -1.45 | 0.090 | 0.003010324 |
| 538827 | ENSBTAG00000002362 | *APOLD1* | -1.71 | 0.090 | 0.003017072 |
| 282016 | ENSBTAG00000006101 | *PSMD4* | 1.32 | 0.090 | 0.003106523 |
| 507751 | ENSBTAG00000002243 | *SNX15* | 1.33 | 0.090 | 0.003112223 |
| 514616 | ENSBTAG00000003341 | *DOK2* | 1.43 | 0.090 | 0.003107576 |
| 515287 | ENSBTAG00000002756 | *CDCA2* | 1.29 | 0.090 | 0.003112412 |
| 516626 | ENSBTAG00000012347 | *SLC51A* | 4.06 | 0.090 | 0.003066617 |
| 520918 | ENSBTAG00000012454 | *SLC35A3* | -1.29 | 0.090 | 0.003091475 |
| 521504 | ENSBTAG00000003495 | *KDM7A* | -1.39 | 0.090 | 0.00306261 |
| 526631 | ENSBTAG00000015419 | *ARHGEF37* | -2.19 | 0.090 | 0.003075199 |
| 535065 | ENSBTAG00000002971 | *CUTC* | 1.24 | 0.090 | 0.003064668 |
| 536833 | ENSBTAG00000011287 | *ATXN7* | -1.27 | 0.090 | 0.003053192 |
| 538094 | ENSBTAG00000005865 | *MAPK6* | -1.68 | 0.090 | 0.003111034 |
| 616224 | ENSBTAG00000048824 | *PET100* | 1.39 | 0.090 | 0.003035057 |
| 782603 | ENSBTAG00000021386 | *ANGPTL3* | -1.55 | 0.090 | 0.003074614 |
| 540101 | ENSBTAG00000024482 | *n/a* | -1.37 | 0.090 | 0.003121734 |
| 516069 | ENSBTAG00000006618 | *HLF* | -1.89 | 0.091 | 0.003138425 |
| 525380 | ENSBTAG00000016354 | *IER2* | 1.35 | 0.091 | 0.003143524 |
| 539324 | ENSBTAG00000016713 | *TMEM30B* | -1.30 | 0.091 | 0.003145304 |
| 511844 | ENSBTAG00000010432 | *EIF2D* | 1.27 | 0.091 | 0.00315223 |
| 513971 | n/a | *n/a* | 3.23 | 0.091 | 0.003161928 |
| 511676 | ENSBTAG00000007728 | *PES1* | 1.43 | 0.091 | 0.003186264 |
| 516258 | ENSBTAG00000018922 | *TRMT10B* | 1.29 | 0.091 | 0.003188142 |
| 519251 | ENSBTAG00000001083 | *MAP3K20* | -1.47 | 0.091 | 0.003188458 |
| 511438 | n/a | *n/a* | -1.48 | 0.091 | 0.0032081 |
| 280717 | ENSBTAG00000017121 | *ALB* | -1.57 | 0.092 | 0.003241534 |
| 507473 | ENSBTAG00000012619 | *CNPPD1* | -1.42 | 0.092 | 0.003239279 |
| 508598 | ENSBTAG00000010566 | *TMEM11* | 1.35 | 0.092 | 0.003225399 |
| 516934 | ENSBTAG00000020900 | *RPUSD4* | 1.35 | 0.092 | 0.003248387 |
| 614102 | ENSBTAG00000025571 | *MRPS18A* | 1.36 | 0.092 | 0.00325027 |
| 534781 | ENSBTAG00000021069 | *PBK* | -3.06 | 0.092 | 0.003258044 |
| 520159 | ENSBTAG00000004494 | *B4GALNT1* | -1.92 | 0.092 | 0.003277864 |
| 507081 | ENSBTAG00000004996 | *ABITRAM* | 1.37 | 0.092 | 0.003304348 |
| 513047 | ENSBTAG00000025211 | *ING1* | 1.38 | 0.092 | 0.003309593 |
| 513056 | ENSBTAG00000005788 | *VANGL1* | -1.43 | 0.092 | 0.003310918 |
| 540378 | ENSBTAG00000010422 | *MDM2* | -1.42 | 0.092 | 0.003288456 |
| 619100 | ENSBTAG00000027326 | *TP53RK* | 1.34 | 0.092 | 0.003312454 |
| 507674 | ENSBTAG00000017750 | *RHBDD3* | 1.32 | 0.093 | 0.003352539 |
| 521926 | ENSBTAG00000019510 | *EIF2B4* | 1.30 | 0.093 | 0.003368617 |
| 281067 | ENSBTAG00000006589 | *CFTR* | -1.47 | 0.093 | 0.0033859 |
| 512771 | ENSBTAG00000015314 | *ASL* | -1.46 | 0.094 | 0.003408284 |
| 512943 | ENSBTAG00000019574 | *MAPK12* | 1.41 | 0.094 | 0.003405016 |
| 494004 | ENSBTAG00000008505 | *APOB* | -1.30 | 0.094 | 0.003424243 |
| 523966 | ENSBTAG00000019163 | *CYB561D2* | 1.41 | 0.094 | 0.003424751 |
| 286813 | ENSBTAG00000022471 | *TTPA* | -1.54 | 0.094 | 0.003446023 |
| 399681 | ENSBTAG00000022314 | *EMD* | 1.25 | 0.094 | 0.003458255 |
| 505531 | ENSBTAG00000013716 | *NR2C2* | -1.23 | 0.094 | 0.003476063 |
| 519566 | ENSBTAG00000015539 | *RAPGEF5* | -1.35 | 0.094 | 0.003460203 |
| 529366 | ENSBTAG00000021164 | *SLMAP* | -1.25 | 0.094 | 0.003468542 |
| 535699 | ENSBTAG00000013187 | *DLG5* | -1.25 | 0.094 | 0.003472094 |
| 536793 | ENSBTAG00000016457 | *FXR1* | -1.23 | 0.094 | 0.003476509 |
| 508878 | ENSBTAG00000001193 | *UNC93B1* | 1.24 | 0.094 | 0.003483338 |
| 534257 | ENSBTAG00000007898 | *CYBRD1* | -1.51 | 0.094 | 0.003492224 |
| 338066 | ENSBTAG00000021818 | *ADGRE5* | 1.53 | 0.094 | 0.003514925 |
| 514787 | ENSBTAG00000011798 | *STK38L* | -1.30 | 0.094 | 0.003510882 |
| 783414 | ENSBTAG00000030915 | *ISY1* | 1.22 | 0.094 | 0.00350973 |
| 527058 | ENSBTAG00000019695 | *RLF* | -1.29 | 0.094 | 0.003530496 |
| 533029 | ENSBTAG00000015144 | *GMIP* | 1.25 | 0.094 | 0.00353426 |
| 538445 | ENSBTAG00000020160 | *RNF138* | -1.33 | 0.094 | 0.003535415 |
| 507867 | ENSBTAG00000020610 | *ANKZF1* | 1.26 | 0.094 | 0.003542371 |
| 404160 | ENSBTAG00000022329 | *SLCO1B3* | -1.33 | 0.094 | 0.00359684 |
| 512584 | ENSBTAG00000013593 | *SNRPA1* | 1.31 | 0.094 | 0.003611663 |
| 513266 | ENSBTAG00000007312 | *CD209* | 2.02 | 0.094 | 0.003603798 |
| 516090 | ENSBTAG00000014970 | *LAMTOR5* | 1.29 | 0.094 | 0.00360868 |
| 520720 | ENSBTAG00000006040 | *UHRF1BP1L* | -1.40 | 0.094 | 0.0035919 |
| 527140 | ENSBTAG00000008483 | *CALCRL* | -1.50 | 0.094 | 0.003573385 |
| 541110 | ENSBTAG00000040001 | *MARVELD2* | -1.30 | 0.094 | 0.003574499 |
| 617304 | ENSBTAG00000025148 | *FBXL12* | 1.35 | 0.094 | 0.003571118 |
| 617345 | ENSBTAG00000039160 | *VAV1* | 1.49 | 0.094 | 0.003608298 |
| 282306 | ENSBTAG00000009232 | *PIK3CA* | -1.46 | 0.094 | 0.003626823 |
| 528655 | ENSBTAG00000016836 | *PDK1* | -1.45 | 0.095 | 0.003659187 |
| 613674 | ENSBTAG00000006296 | *VMA21* | -1.23 | 0.095 | 0.003659026 |
| 618580 | ENSBTAG00000001394 | *TMED9* | 1.44 | 0.095 | 0.003664986 |
| 504390 | ENSBTAG00000012595 | *RIDA* | 1.32 | 0.095 | 0.003688718 |
| 513277 | ENSBTAG00000004416 | *NUDC* | 1.29 | 0.095 | 0.00370178 |
| 540606 | ENSBTAG00000017905 | *WDR91* | 1.32 | 0.095 | 0.003695573 |
| 286819 | ENSBTAG00000014724 | *EIF4A2* | -1.28 | 0.096 | 0.003723115 |
| 616339 | ENSBTAG00000011846 | *TUT1* | 1.25 | 0.096 | 0.00372555 |
| 515662 | ENSBTAG00000016553 | *EME2* | 1.27 | 0.096 | 0.003734112 |
| 781067 | ENSBTAG00000001573 | *JMJD1C* | -1.29 | 0.096 | 0.003751731 |
| 506900 | ENSBTAG00000039657 | *HIST1H2AC* | 2.27 | 0.096 | 0.003763152 |
| 511748 | ENSBTAG00000050962 | *YIPF1* | -1.53 | 0.096 | 0.00377613 |
| 782793 | ENSBTAG00000015599 | *PRKD2* | -1.31 | 0.096 | 0.003770644 |
| 508684 | ENSBTAG00000001763 | *NR2C2AP* | 1.39 | 0.096 | 0.00379157 |
| 510259 | ENSBTAG00000020930 | *GEMIN2* | 1.67 | 0.096 | 0.003797185 |
| 539471 | n/a | *n/a* | -1.53 | 0.096 | 0.00379077 |
| 541172 | ENSBTAG00000021756 | *ZDHHC17* | -1.70 | 0.096 | 0.00380656 |
| 508788 | ENSBTAG00000008292 | *SNRPD1* | 1.42 | 0.097 | 0.003855191 |
| 280847 | ENSBTAG00000001745 | *LUM* | -1.62 | 0.098 | 0.003880003 |
| 369023 | ENSBTAG00000016253 | *G6PC3* | 1.30 | 0.098 | 0.003893129 |
| 100847890 | n/a | *n/a* | 1.46 | 0.098 | 0.003901138 |
| 523874 | ENSBTAG00000005932 | *FAM184B* | 2.38 | 0.099 | 0.003946552 |
| 782922 | ENSBTAG00000022570 | *n/a* | 3.72 | 0.099 | 0.003939903 |
| 516026 | ENSBTAG00000039509 | *GPRC5A* | -2.05 | 0.099 | 0.003954192 |
| 100139910 | ENSBTAG00000014205 | *PRKAR2A* | -1.74 | 0.099 | 0.003977459 |
| 522378 | ENSBTAG00000007620 | *INTS11* | 1.22 | 0.099 | 0.004009223 |
| 532574 | ENSBTAG00000003763 | *MYO5C* | -1.53 | 0.099 | 0.004001764 |
| 616767 | ENSBTAG00000020731 | *PRRG4* | -2.81 | 0.099 | 0.004009618 |
| 507054 | ENSBTAG00000018347 | *IL33* | -1.48 | 0.099 | 0.004019835 |
| 614069 | ENSBTAG00000007387 | *ENY2* | 1.21 | 0.099 | 0.004029686 |
| 614408 | ENSBTAG00000012250 | *BLOC1S6* | -1.39 | 0.100 | 0.004043502 |
| 507297 | ENSBTAG00000022847 | *HEATR5A* | -1.32 | 0.100 | 0.004072372 |
| 523042 | ENSBTAG00000025443 | *FNIP1* | -1.38 | 0.100 | 0.004069531 |
| 286796 | ENSBTAG00000038842 | *NEDD8* | 1.28 | 0.100 | 0.004087817 |
| 506986 | ENSBTAG00000002457 | *SEC61B* | 1.43 | 0.100 | 0.004090369 |
