# Supplementary material for: Unique adaptations in neonatal hepatic transcriptome, nutrient signaling, and one-carbon metabolism in response to feeding ethyl cellulose rumen-protected methionine during late-gestation in Holstein cows
Source: BMC Genomics. 2021 Apr 17;22:280. doi: 10.1186/s12864-021-07538-w (PMC8053294; doi:10.1186/s12864-021-07538-w)
Supplement: Supplementary file 3 — Additional File 3: List of all transcription factor (TF) obtained with ChEA3 transcription factor enrichment analysis tool and significantly overrepresented (FDR ≤ 0.05) in the differentially expressed gene (DEG) list from RNAseq data in liver tissue of 4-d old Holstein calves (n = 6/group) born to cows randomly assigned to receive a basal control (CON) diet from − 28 ± 2 d to parturition [1.47 Mcal/kg dry matter (DM) and 15.3% crude protein (CP)] with no added Met or CON plus ethyl cellulose Met (MET, Mepron®, Evonik Nutrition & Care GmbH, Germany). For each TF, the ranking (Rank), P value, false discovery rate (FDR), the overlapping genes among the DEG list, and the predicted state (i.e. impact and flux value) calculated using the rationale underlying the Dynamic Impact Approach (DIA) analysis are reported. [file 12864_2021_7538_MOESM3_ESM.docx]

**Additional File 3:** List of all transcription factor (TF) obtained with ChEA3 transcription factor enrichment analysis tool and significantly overrepresented (FDR ≤ 0.05) in the differentially expressed genes (DEG) list from RNAseq data of liver tissue of calves (n = 6/group) from Holstein cows randomly assigned to receive a basal control (CON) close-up diet (from −28 ±2 d to parturition) [1.47 Mcal/kg dry matter (DM) and 15.3% crude protein (CP)] with no added Met or CON plus ethyl cellulose Met (MET, Mepron®, Evonik Nutrition & Care GmbH, Germany). For each TF, the ranking (Rank), P value, false discovery rate (FDR), the overlapping genes among the DEG list, and the predicted state (i.e. impact and flux value) calculated using the rationale underlying the Dynamic Impact Approach (DIA) analysis are reported.

| **TF** | **Impact** | **Flux** | **Predicted State** | **Rank** | **P value** | **FDR** | **Overlapping DEG** |
| --- | --- | --- | --- | --- | --- | --- | --- |
| MYC | 178.42 | 57.40 | Activated | 1 | 4.11E-09 | 1.26E-06 | *POP1, GMFG, SMC4, KRTCAP2, CDK5RAP3, IER2, CLPP, DDIT3, UBA52, RPL28, NPRL2, ABCB8, ANAPC11, BLOC1S6, PRKAR2A, TOE1, NR1D2, NFATC4, MRPL38, ABCC10, CYB561D2, MCEE, GSTO1, SUMF1, RNF167, TUT1, SF3B5, RIDA, PSMB2, BID, SF3A2, TARBP2, LAMTOR2, HSPA1A, STK16, MAP2K3, CNPY3, LENG1, SNRPA, ENY2, INO80E, GOT1, DHCR24, FOXN2, G6PC3, TMEM11, RAD9A, BMPR2, ZC3H3, MRPL4, NGRN, GPX1, REXO4, EVC, SNRPA1, ZBTB44, MRM1, NCLN, POLR2I, POLR2J, ATOX1, KLF5, KLF9, POLR3H, EIF4A2, SMARCB1, NUBP1, NUBP2, SNRPD1, RPL36AL, PDK1, ALG5, MRPS18B, MRPS18A, ALG3, IMP4, SNX4, PTDSS2, VAT1, ALDH6A1, CALM3, SUGP1, FKBP11, SLC44A4, GRIK5, GUK1, SEC61B, PRMT6, TMED9, NME1, APRT, RPUSD4, KIF2C, YAP1, NEDD8, NT5C, PES1, NPM3, GEMIN2, NR3C1, CHCHD1, ZGPAT, DPM2, DOK2, CCNE2, CCNB1, SHARPIN, DHRS7B, RHBDD3, NONO, DLG1, NABP2, COPE, CHD9, DDX41, YWHAG, PEX1, EEF1D, DOCK1, PFDN6, PNKP, PSMB10, TBRG4, PRRG4, RMND5A, MAPK6, SLC50A1, WEE1* |
| E2F1 | 178.13 | 31.67 | Activated | 2 | 7.28E-08 | 7.45E-06 | *SMC4, SMC2, MYC, MPV17, CDK5RAP3, IER2, VPS18, AARSD1, RNF125, CLPP, DDIT3, RPL28, RPN1, ABCB8, ANAPC11, BLOC1S6, MCTS1, TOE1, MCAT, VANGL1, ADI1, MMS19, MRPL38, ING1, NUDC, ANXA4, SUMF1, CDC42EP5, TUT1, SF3B5, AHNAK, RNF138, BID, SF3A2, TARBP2, DCBLD2, SNX15, ATCAY, STK16, CNPY2, LENG1, UTRN, ENY2, FECH, FOXO4, ATXN7, AMDHD2, EIF2B4, GOT1, DHCR24, FOXN2, TMEM11, RAD9A, MRPL4, NGRN, GPX1, REXO4, COASY, SNRPA1, TSSC4, GTF3C5, HM13, MRM1, SH3BP2, POLR2G, FNIP1, KCNJ2, FOXJ3, GFER, ATOX1, KLF5, KLF9, SLC25A30, POLR3H, EIF4A2, SMARCB1, NUBP1, NUBP2, SNRPD1, RPL36AL, EIF2D, TNS3, PDK1, CPT1A, ACTN2, MRPS18B, MRPS18A, ALG3, IMP4, PLPP3, PTDSS2, RACGAP1, VAT1, EAF2, FKBP11, INTS11, MRPS11, CITED4, ROBO1, ACADM, PRMT6, ZFX, ZDHHC17, NME1, APRT, KIF2C, YAP1, UQCR11, TYMS, NT5C, PES1, SCAND1, YIPF1, ITCH, GEMIN2, CHCHD1, HIST1H2AC, ZGPAT, ACYP2, ALDH3A2, DPM2, CCNE2, RPP21, COX19, DPP4, SHARPIN, DHRS7B, ECT2, KLHL26, NONO, HSPA2, TJAP1, BRWD1, PICALM, DDX41, ADGRE5, ARHGEF10, TSFM, PEX1, ETV5, TRAPPC6A, EEF1D, CAT, PFDN6, PNKP, PPP2R3C, TAF10, WEE1, CDK2AP2* |
| CREB1 | 174.61 | 24.88 | Activated | 3 | 2.87E-07 | 2.20E-05 | *KRTCAP2, IER2, CHID1, VPS18, BIN3, BIN1, DDIT3, FDX2, PRKD2, UBA52, EXD2, CDCA2, ANAPC11, BLOC1S6, MCTS1, IRF2BP2, TYSND1, NR1D2, FOSL2, MMS19, ING1, CYB561D2, EMD, TUT1, PPARA, SF3B5, PSMB2, TARBP2, SNX15, LAMTOR2, CHPF, LTN1, GOLIM4, FADS2, STK16, MAP2K3, CNPY3, SNRPA, ENY2, RLF, EIF2B4, GOT1, TMEM11, CPD, ZC3H3, MRPL4, DAD1, TSSC4, HM13, CXXC1, NCLN, SEC11C, SPAG9, OXR1, KLF6, KLF5, KLF9, TRMT61A, EIF4A2, PANK3, JMJD1C, TXNDC16, NUBP2, EFR3A, GOLPH3L, PREB, APOB, ZRANB1, EAF2, NDUFAF3, CALM3, SUGP1, GUK1, TMED9, KIF2C, AMIGO1, RTL6, CHCHD1, ALG12, BRMS1, SHARPIN, ANKZF1, CBX8, RHBDD3, DENND4A, NONO, NABP2, GMPPA, YWHAG, YWHAZ, ETV5, EEF1D, HLF, NR2C2, PSMB10, MAPK6, TAF10, WEE1, CDK2AP2, RBM42* |
| PPARG | 178.93 | -68.11 | Inhibited | 4 | 5.64E-07 | 3.46E-05 | *ZC3H12D, SCP2, MYC, NAMPT, WDR91, IER2, CHID1, AARSD1, HDHD5, RNF125, BIN3, CLPP, DDIT3, PRKD2, RPL28, RPN1, MRPL16, PRKAR2A, IRF2BP2, RMDN2, FOSL2, FERMT1, PAH, MTMR10, EEA1, GSTO1, ANXA5, SUMF1, KAT2B, HEATR5A, AEBP2, TUT1, PLCB1, AHNAK, BID, RNLS, BBOX1, TARBP2, LAMTOR5, UHRF1BP1L, MYLK4, FADS2, CDH2, ZBTB39, UTRN, ENY2, AMDHD2, GOT1, FOXN2, CDK1, MDM2, SERINC5, NGRN, GPX1, PIK3CA, MET, GTF3C5, ROCK2, GLRX, MRM1, FNIP1, POLR2J, CYB5A, FOXJ3, DDHD2, PTPN12, CDC42BPA, KBTBD11, BICC1, KLF6, NAGK, KLF9, SLC25A30, PANK3, SMARCB1, TNS3, CPT1A, ECH1, MRPS18B, MRPS18A, PREB, PLPP3, CFTR, STK38L, PCK1, ZRANB1, MYO5C, CUTC, IDE, ROBO1, ACADM, ZDHHC17, RPUSD4, CD47, C1QB, SH3BGRL, KLB, GEMIN2, SSC4D, SELENOP, COX19, COBLL1, ACBD5, CRIM1, HOOK3, ARHGAP25, TJAP1, DLG1, GCLM, PICALM, CHD9, TNFAIP3, PPP1R9B, ADGRE5, YWHAG, ARHGEF10, PEX1, YWHAZ, ACOX1, CAT, KDM7A, USP12, NR2C2, PSMB10, RMND5A, TNKS2, MAPK6, TMEM30B, CMTM4* |
| KLF1 | 165.60 | 73.79 | Activated | 6 | 9.92E-07 | 4.19E-05 | *SPI1, KRTCAP2, MYC, AARSD1, CLPP, RPN1, TYSND1, VANGL1, ADI1, MRPL38, EMD, GSTO1, TUT1, SNX15, SMCHD1, SNRPA, FECH, CORO1A, EIF2B4, GOT1, DHCR24, COPS9, SORD, SNRPA1, HM13, CXXC1, NCLN, SEC11C, NUBP2, SNRPD1, SOX6, PDK1, ECH1, IMP4, PREB, SYMPK, CALM3, IDE, RAC2, TMED9, UQCR11, NEDD8, NT5C, CHCHD1, HIST1H2AC, CYBA, BRMS1, RPP21, PPIH, RPS19BP1, ACBD5, NABP2, GCLM, PPP1R9B, YWHAG, TRAPPC6A, CAT, PFDN6, TBRG4, TAF10* |
| SPI1 | 173.45 | 5.80 | Activated | 5 | 9.49E-07 | 4.19E-05 | *SPI1, GMFG, SLC4A4, SCP2, PSMD4, NAMPT, CDK5RAP3, IER2, RNF125, PRKD2, RPL28, EXD2, CFP, BLOC1S6, WDR70, MCTS1, NR1D2, NFATC4, FERMT1, PYGL, ING1, PQBP1, EEA1, TRMT112, MRPL42, GSTO1, ANXA4, GMIP, SUMF1, RNF167, TUT1, PPARA, FAM214A, APOLD1, BID, PET100, LAPTM5, PSMB8, LAMTOR2, GOLIM4, MYLK4, PYCARD, ATCAY, STK16, MAP2K3, TPM1, CNPY3, CLIP1, CORO1A, YBEY, AMDHD2, MAP3K20, GOT1, CYBRD1, DHCR24, FOXN2, BFSP1, G6PC3, CPD, CCDC22, SERINC5, NGRN, PSTPIP1, SORD, DAD1, ROCK2, UNC93B1, CXXC1, GLRX, HSD17B11, HECTD1, SH3BP2, POLR2G, PTPN18, PTPN12, OXR1, FBXL12, SLC25A30, PANK3, JMJD1C, MRPS18B, MAP7, STK38L, ASL, CD14, STK11IP, STAG2, NDUFAF3, RAC2, TMED9, ZFX, ZDHHC17, VAV1, C1QB, TOR4A, AIF1, PES1, C1QC, WASHC4, NR3C1, EME2, KNTC1, ZGPAT, ACYP2, CYBA, TYROBP, RPS19BP1, CCNB1, CBX8, ACBD5, HOOK3, ARHGAP25, BRWD1, PICALM, CHD9, PPP1R9B, GMPPA, CNPPD1, YWHAZ, ETV5, CAT, PNKP, USP12, TBRG4, STOM, TAF10, SLC50A1, BUD13* |
| ZFX | 170.08 | 15.72 | Activated | 7 | 1.23E-06 | 4.19E-05 | *NCKAP1, PSMD4, NAMPT, IER2, AARSD1, HDHD5, CLPP, UBA52, RPL28, RPN1, ABCB8, ANAPC11, PRKAR2A, TTPA, TOE1, MCAT, NR0B2, VANGL1, ADI1, MRPL38, EEA1, EMD, NUDC, GSTO1, SUMF1, AHNAK, PSMB2, RNF138, SC5D, BID, SF3A2, TARBP2, DCBLD2, STK16, ZBTB39, CNPY2, CNPY3, LENG1, CLIP1, FKBP9, SNRPA, ENY2, FECH, ATXN7, NEK9, FOXN2, BFSP1, G6PC3, MDM2, RAD9A, ISY1, MRPL4, REXO4, COASY, EVC, SNRPA1, GTF3C5, ZBTB44, FNIP1, FOXJ3, ATOX1, SLC25A30, POLR3H, EIF4A2, PANK3, SMARCB1, SNRPD1, FBXO3, TNS3, PDK1, MRPS18A, NEIL2, FNDC4, SNX4, PTDSS2, STK38L, RAC2, PRMT6, ZFX, ZDHHC17, VAV1, AMIGO1, PES1, SCAND1, ITCH, GEMIN2, RTL6, ZGPAT, CYBA, ALDH3A2, DPM2, CCNE2, RHBDD3, KLHL26, NONO, TJAP1, NABP2, GCLM, BRWD1, CHD9, ADGRE5, ETV5, TRAPPC6A, LATS2, ACOX1, EEF1D, ZMYND11, CLN5, TBRG4, MARVELD2, MAPK6, TMEM30B, CMTM4, WEE1, CDK2AP2, BUD13* |
| CEBPB | 167.51 | 36.64 | Activated | 8 | 1.79E-06 | 5.00E-05 | *MYC, CHID1, AARSD1, BIN3, RUFY3, FDX2, CFP, NR2C2AP, TOE1, TYSND1, RMDN2, MCAT, ABCC10, CYB561D2, EMD, PCMTD1, GMIP, RNF167, PLCB1, PDF, PSMB8, LAMTOR5, CHPF, MPV17L2, STK16, ZBTB39, CNPY2, INSIG2, FBXO48, FOXO4, RLF, IP6K2, BCL3, RAD9A, COPS9, NGRN, CXXC1, MRM1, POLR2G, FOXJ3, PTPN12, OXR1, GFER, KBTBD11, FBXL12, KLF6, SLC25A30, NUBP2, RPL36AL, ECH1, IMP4, PREB, SNX4, STK38L, B4GALNT1, ZRANB1, FKBP11, IDE, GUK1, STBD1, SEC61B, PRMT6, TMED9, CYBA, CBX8, RHBDD3, PICALM, ARHGEF10, TSFM, EEF1D, NR2C2, MAPK6, LUM, TAF10, SLC50A1, MAPK12, CDK2AP2, BUD13* |
| HNF4A | 175.29 | -51.68 | Inhibited | 9 | 3.65E-06 | 9.34E-05 | *NCKAP1, ZC3H12D, KRTCAP2, SCP2, PSMD4, MYC, RUFY3, CDCA2, NPRL2, MRPL16, WDR70, PRKAR2A, TTPA, IRF2BP2, TYSND1, NR0B2, FOSL2, FERMT1, ADI1, PAH, MMS19, HRG, MYOM1, MTMR10, PYGL, ABCC10, EEA1, MRPL42, NUDC, GSTO1, ANXA4, ANXA5, SUMF1, HEATR5A, TUT1, PPARA, PLCB1, RAPGEF5, SF3B5, AHNAK, PDF, CHN2, BID, KIDINS220, BBOX1, DCBLD2, GATM, AADAC, SLMAP, SNX15, LAMTOR5, UHRF1BP1L, CHPF, GOLIM4, PLOD2, IL1RAP, MYLK4, PYCARD, CDH2, MAP2K3, CNPY3, CLIP1, UTRN, CALCRL, INSIG2, AMDHD1, ATXN7, AMDHD2, ANKRD29, EIF2B4, GOT1, DHCR24, FOXN2, TMEM11, CPD, ALB, SLCO1A2, BCL3, SERINC5, ZNF771, ISY1, ZC3H3, MRPL4, PSTPIP1, REXO4, COASY, EVC, PCCA, DAD1, MET, TSSC4, GTF3C5, ROCK2, UNC93B1, HM13, GLRX, HSD17B11, HECTD1, NCLN, SH3BP2, FNIP1, POLR2I, SEC11C, SPAG9, CYB5A, METAP1D, SLC35A3, FOXJ3, PTPN12, CDC42BPA, OXR1, BICC1, ATOX1, FBXL12, KLF6, POLR3H, EIF4A2, JMJD1C, TXNDC16, SOX6, TNS3, CPT1A, ACTN2, GOLPH3L, MRPS18A, PLPP3, SNX4, RACGAP1, HLTF, STK38L, SYMPK, ASL, STXBP5, CD14, PCK1, APOB, STK11IP, ALDH6A1, STAG2, MYO5C, CALM3, CUTC, SUGP1, ROBO2, ROBO1, SH3PXD2B, ACADM, TMED9, ZFX, NME1, MICU3, YAP1, UQCR11, NEDD8, AIF1, PES1, SCAND1, IL33, YIPF1, ITCH, KLB, SSC4D, NR3C1, SRP14, SBF2, ZGPAT, IL11RA, LMO7, ACYP2, ALDH3A2, BRMS1, SELENOP, PPIH, COX19, DPP4, SHARPIN, COBLL1, DHRS7B, ACBD5, TCAF1, ARHGEF37, DENND4A, NONO, CRIM1, HOOK3, TJAP1, DLG1, DLG5, COPE, PICALM, CHD9, DOCK7, TNFAIP3, GMPPA, YWHAG, PEX1, YWHAZ, LATS2, RABEP2, ACOX1, ALDH1A1, CAT, DOCK1, HLF, PSMB10, PRRG4, TNKS2, MARVELD2, PPP2R3C, STOM, CMTM4, ATP2B1, WEE1, RBM42, SNTB1* |
| FOXO1 | 181.39 | -36.48 | Inhibited | 10 | 7.35E-06 | 1.73E-04 | *GMFG, ZC3H12D, IER2, VPS18, HDHD5, RNF125, BIN1, UBA52, RPN1, MCTS1, IRF2BP2, NR1D2, FOSL2, MMS19, MTMR10, PYGL, ING1, PCMTD1, AEBP2, AHNAK, LAPTM5, LAMTOR5, FADS2, SMCHD1, TPM1, UTRN, IP6K2, GOT1, RAD9A, ISY1, PTPN21, PSTPIP1, DAD1, HM13, GLRX, MRM1, HECTD1, PTPN18, FOXJ3, KBTBD11, KLF6, NAGK, TRMT61A, TXNDC16, PDK1, MRPS18A, PLPP3, NEIL1, ZRANB1, STK11IP, STAG2, GUK1, SH3PXD2B, RAC2, ACADM, ZFX, CD47, PTGER3, SH3BGRL, SSC4D, NR3C1, SRP14, GPRIN3, TYROBP, CCNE2, RPP21, COBLL1, ECT2, ACBD5, DENND4A, ARHGAP25, GCLM, PICALM, TNFAIP3, PPP1R9B, ADGRE5, USP12, MAPK6, SNTB1* |
| ELF1 | 153.60 | 90.69 | Activated | 11 | 8.03E-06 | 1.76E-04 | *RNF167, SF3B5, SNRPA, GTF3C5, NCLN, PES1, DOK2, BRMS1, COPE, YWHAZ, TRAPPC6A, ACOX1, PFDN6* |
| FOXA2 | 193.24 | -70.10 | Inhibited | 12 | 1.06E-05 | 2.16E-04 | *RNF125, RUFY3, CDCA2, WDR70, NEBL, IRF2BP2, NR1D2, NR0B2, PAH, PYGL, PCMTD1, GSTO1, ANXA4, ANXA5, TUT1, PLCB1, RAPGEF5, AHNAK, APOLD1, PSMB2, CHN2, SC5D, SQOR, BBOX1, GATM, UHRF1BP1L, CHPF, IL1RAP, CDH2, STK16, ZBTB39, CNPY3, CLIP1, UTRN, INSIG2, AMDHD1, AMDHD2, MAP3K20, ANKRD29, GOT1, DHCR24, FOXN2, BFSP1, G6PC3, CPD, SLCO1A2, SERINC5, ZNF771, BMPR2, ZC3H3, PTPN21, REXO4, PIK3CA, PCCA, DAD1, MET, ROCK2, HM13, HSD17B11, HECTD1, POLR2I, SPAG9, METAP1D, FOXJ3, OXR1, BICC1, KLF6, KLF5, SOX6, TNS3, CPT1A, ECH1, MRPS18A, PLPP3, CD14, PCK1, APOB, ALDH6A1, MYO5C, CUTC, SLC44A4, ROBO1, SH3PXD2B, TMED9, MICU3, KLB, SRP14, SBF2, ZGPAT, ACYP2, ALDH3A2, DOK2, BRMS1, CCNE2, SELENOP, COBLL1, ECT2, CBX8, DENND4A, CRIM1, HOOK3, DLG1, DLG5, COPE, CHD9, DOCK7, TNFAIP3, ETV5, ACOX1, HLF, PNKP, STOM, ATP2B1, WEE1, SNTB1* |
| E2F4 | 171.37 | -24.18 | Inhibited | 13 | 1.78E-05 | 3.41E-04 | *KRTCAP2, CHID1, MRPL16, TOE1, IRF2BP2, TYSND1, VANGL1, MMS19, MTMR10, PYGL, ING1, EEA1, TRMT112, NUDC, GSTO1, RNF167, AHNAK, FAM214A, APOLD1, PDF, PSMB2, SC5D, SQOR, TARBP2, GATM, LAMTOR2, LAMTOR5, PYCARD, FADS2, TPM1, CNPY2, CLIP1, SNRNP25, INO80E, RLF, AMDHD1, AMDHD2, NEK9, DHCR24, TMEM11, CDK1, MDM2, RAD9A, NGRN, PCCA, SNRPA1, TSSC4, UNC93B1, ZBTB44, HECTD1, SLC35A3, GFER, REC8, KLF6, KLF5, SLC25A35, JMJD1C, TXNDC16, NUBP1, NUBP2, RPL36AL, EIF2D, ZFPL1, ALG5, PLPP3, PTDSS2, RACGAP1, B4GALNT1, CUTC, INTS11, MRPS11, CITED4, IDE, ACADM, PRMT6, ZDHHC17, APRT, KIF2C, YAP1, PTGER3, WASHC4, EME2, KNTC1, SBF2, CHCHD1, ALDH3A2, BRMS1, PPIH, DHRS7B, ACBD5, HSPA2, DLG5, NABP2, GCLM, CHD9, DOCK7, LATS2, RABEP2, CPTP, ZMYND11, USP12, CLN5, PRRG4, TNKS2, MAPK6, TMEM30B, CMTM4, TAF10, SLC50A1, WEE1, CDK2AP2, BUD13* |
| MYB | 177.28 | -17.66 | Inhibited | 14 | 1.96E-05 | 3.54E-04 | *SPI1, MYC, DDIT3, PYGL, EEA1, TRMT112, NUDC, GSTO1, BID, SQOR, SLMAP, IL1RAP, MAP2K3, AMDHD2, G6PC3, HM13, GLRX, ZBTB44, HSD17B11, POLR2I, CYB5A, PTPN12, OXR1, KLF6, KLF5, KLF9, TNS3, PDK1, FXR1, B4GALNT1, STXBP5, NDUFAF3, CALM3, STBD1, APRT, IL33, ITCH, ALDH3A2, HSPA2, GCLM, CHD9, ADGRE5, ETV5, BUD13* |
| THAP11 | 174.35 | 108.94 | Activated | 15 | 2.15E-05 | 3.67E-04 | *POP1, HDHD5, CLPP, NPRL2, ABCB8, WDR70, ING1, CYB561D2, TUT1, RIDA, TARBP2, LAMTOR2, SNRPA, ENY2, EIF2B4, TMEM11, REXO4, DAD1, TSSC4, NCLN, SEC11C, PANK3, PTDSS2, SUGP1, PRMT6, TMED9, NME1, KIF2C, NEDD8, DPM2, RPP21, RPS19BP1, SHARPIN, RHBDD3, COPE, GMPPA, YWHAG* |
| GFI1B | 156.17 | 24.10 | Activated | 16 | 3.26E-05 | 5.27E-04 | *SPI1, ZC3H12D, IER2, VPS18, RNF125, RPL28, FOSL2, MRPL38, ING1, EEA1, TRMT112, GMIP, KAT2B, RNF167, SF3B5, PSMB2, RNF138, LAPTM5, SNX15, UHRF1BP1L, STK16, UBR3, FOXN2, BMPR2, PTPN21, MRPL4, GPX1, SNRPA1, ROCK2, HM13, FNIP1, POLR2J, FOXJ3, NAGK, EIF4A2, NUBP1, EIF2D, PDK1, IMP4, HLTF, B4GALNT1, NDUFAF3, CUTC, SLC44A4, VAV1, CD48, SLU7, PTGER3, NEDD8, NPM3, ITCH, CHCHD1, ZGPAT, TYROBP, ANKZF1, CBX8, ACBD5, RHBDD3, CRIM1, HOOK3, GCLM, PICALM, CHD9, GMPPA, PEX1, RABEP2, EEF1D, PNKP, CLN5, TNKS2, MVB12A, MAPK12* |
| MECOM | 165.92 | 0.24 | Activated | 18 | 4.51E-05 | 6.60E-04 | *GMFG, SMC4, KRTCAP2, IER2, RNF125, BIN3, PRKD2, EXD2, IRF2BP2, FOSL2, PYGL, EMD, KAT2B, HEATR5A, BID, SQOR, LAPTM5, SLMAP, UHRF1BP1L, GOLIM4, PYCARD, STX7, CNPY3, LENG1, CLIP1, CALCRL, CORO1A, CPD, COPS9, ISY1, PSTPIP1, COASY, CXXC1, GLRX, HSD17B11, MRM1, SEC11C, SPAG9, SLC25A30, SLC25A35, JMJD1C, ECH1, PREB, ASL, B4GALNT1, STXBP5, VAT1, CUTC, SUGP1, INTS11, RAC2, APRT, MICU3, PTGER3, PES1, SCAND1, ALG12, CYBA, DOK2, SHARPIN, DHRS7B, ECT2, ACBD5, DENND4A, ARHGAP25, TNFAIP3, ADGRE5, YWHAG, YWHAZ, PNKP, CLN5, ATP2B1* |
| SRF | 173.51 | 5.77 | Activated | 17 | 4.49E-05 | 6.60E-04 | *POP1, RPN1, TOE1, MMS19, ING1, PCMTD1, GSTO1, ANXA5, SUMF1, TUT1, SF3B5, AHNAK, TRMT10B, GOLIM4, CNPY2, UTRN, SNRPA, FECH, AMDHD2, TMEM11, PTPN21, DAD1, TSSC4, POLR2G, POLR2I, OXR1, KLF6, NAGK, POLR3H, JMJD1C, NUBP2, ECH1, PLPP3, HLTF, STK11IP, CUTC, SUGP1, ZDHHC17, CD47, SLC31A1, GEMIN2, SRP14, EME2, ACYP2, ECT2, ACBD5, CHD9, ARHGEF10, TSFM, PEX1, TRAPPC6A, NR2C2, STOM, SLC50A1* |
| GABPA | 181.03 | -127.76 | Inhibited | 21 | 7.38E-05 | 9.44E-04 | *SLC4A4, WDR91, IRF2BP2, RMDN2, NR1D2, PAH, MMS19, MRPL38, EEA1, RIDA, CHN2, SQOR, SLMAP, GOLIM4, CDH2, TPM1, UTRN, INSIG2, MDM2, PIK3CA, ROCK2, POLR2J, KCNJ2, CDC42BPA, OXR1, KLF6, KLF5, KLF9, SLC25A30, EFR3A, CPT1A, GOLPH3L, MRPS18A, SNX4, CUTC, ZDHHC17, IL33, SSC4D, LMO7, CRIM1, GCLM, YWHAG, YWHAZ, ALDH1A1, CAT, CLN5* |
| NFE2L2 | 181.03 | -127.76 | Inhibited | 20 | 7.38E-05 | 9.44E-04 | *SLC4A4, WDR91, IRF2BP2, RMDN2, NR1D2, PAH, MMS19, MRPL38, EEA1, RIDA, CHN2, SQOR, SLMAP, GOLIM4, CDH2, TPM1, UTRN, INSIG2, MDM2, PIK3CA, ROCK2, POLR2J, KCNJ2, CDC42BPA, OXR1, KLF6, KLF5, KLF9, SLC25A30, EFR3A, CPT1A, GOLPH3L, MRPS18A, SNX4, CUTC, ZDHHC17, IL33, SSC4D, LMO7, CRIM1, GCLM, YWHAG, YWHAZ, ALDH1A1, CAT, CLN5* |
| STAT3 | 180.41 | -92.49 | Inhibited | 19 | 7.24E-05 | 9.44E-04 | *ZC3H12D, MYC, NAMPT, RNF125, DDIT3, PRKD2, IRF2BP2, FOSL2, MMS19, EEA1, ANXA4, AEBP2, RAPGEF5, AHNAK, LAMTOR2, SMCHD1, FKBP9, CPD, ALB, BCL3, ISY1, GLRX, SLC25A30, PANK3, PDK1, IMP4, IDE, RAC2, CD47, NR3C1, KNTC1, DPP4, ACBD5, ARHGAP25, CHD9, TNFAIP3, YWHAZ, ETV5, LATS2, ZMYND11, KDM7A, TBRG4, MAPK6, ATP2B1, WEE1* |
| ESR1 | 202.56 | -68.35 | Inhibited | 22 | 1.15E-04 | 1.41E-03 | *DDIT3, TTPA, IRF2BP2, NR0B2, PAH, PPARA, FMO2, FADS2, NUDT12, CLIP1, INSIG2, GOT1, DHCR24, PCCA, HSD17B11, CPT1A, FNDC4, PTDSS2, PCK1, DPP4, GCLM, PICALM, ACOX1, PSMB10, WEE1* |
| ERG | 163.40 | 24.63 | Activated | 23 | 1.29E-04 | 1.52E-03 | *POP1, SMC2, MYC, NAMPT, CLPP, EXD2, RPN1, BLOC1S6, NR2C2AP, MCTS1, TYSND1, FOSL2, MCEE, PCMTD1, GSTO1, GMIP, KAT2B, RNF167, TUT1, PSMB2, RNF138, LAMTOR5, FADS2, STK16, MAP2K3, ZBTB39, SNRNP25, ENY2, FBXO48, ATXN7, DHCR24, BMPR2, MRPL4, NGRN, PSTPIP1, SORD, COASY, SNRPA1, HM13, ZBTB44, POLR2J, SEC11C, NAGK, TRMT61A, NUBP1, EFR3A, PDK1, ALG5, SNX4, NDUFAF3, SUGP1, SEC61B, TMED9, SLU7, ITCH, CHCHD1, ZGPAT, DPM2, DOK2, RPS19BP1, DHRS7B, ACBD5, GCLM, GMPPA, TRAPPC6A, RABEP2, PNKP, NR2C2, TBRG4, TNKS2, MVB12A* |
| CREM | 165.90 | -14.99 | Inhibited | 24 | 1.43E-04 | 1.62E-03 | *NCKAP1, SMC4, NAMPT, WDR91, CDK5RAP3, CHID1, VPS18, HDHD5, RUFY3, CLPP, DDIT3, FDX2, PRKD2, UBA52, CDCA2, NPRL2, RPN1, MRPL16, BLOC1S6, WDR70, PRKAR2A, TYSND1, NR1D2, FOSL2, MMS19, MTMR10, MRPL38, ABCC10, ING1, TRMT112, EMD, PCMTD1, NUDC, AEBP2, TUT1, PPARA, SF3B5, PDF, PSMB2, RNF138, SC5D, TARBP2, SLMAP, UHRF1BP1L, LTN1, MPV17L2, FADS2, SMCHD1, CDH2, STK16, MAP2K3, CNPY3, CLIP1, SNRNP25, SNRPA, ENY2, FECH, FBXO44, ATXN7, EIF2B4, GOT1, DHCR24, FOXN2, G6PC3, TMEM11, CPD, BCL3, MDM2, SERINC5, ISY1, BMPR2, ZC3H3, MRPL4, NGRN, SGCB, PCCA, DAD1, TSSC4, ROCK2, HM13, CXXC1, ZBTB44, MRM1, HECTD1, FNIP1, SPAG9, SLC35A3, FOXJ3, CDC42BPA, OXR1, GFER, KBTBD11, BICC1, KLF6, KLF5, NAGK, KLF9, POLR3H, TRMT61A, SLC25A35, EIF4A2, SMARCB1, JMJD1C, TXNDC16, NUBP2, EFR3A, RPL36AL, EIF2D, ZFPL1, MRPS18B, PREB, CFTR, PPP1R37, FXR1, SNX4, PTDSS2, RACGAP1, HLTF, STK38L, SYMPK, EAF2, NDUFAF3, CALM3, INTS11, GUK1, PRMT6, TMED9, ZFX, ZDHHC17, APRT, KIF2C, MICU3, CD47, YAP1, AIF1, NPM3, ITCH, KLB, RTL6, SSC4D, SRP14, CHCHD1, BRMS1, CCNE2, ECT2, ANKZF1, CBX8, ACBD5, DENND4A, KLHL26, HSPA2, HOOK3, TJAP1, DLG1, GCLM, BRWD1, PICALM, CHD9, DOCK7, PPP1R9B, YWHAG, ARHGEF10, YWHAZ, ETV5, TRAPPC6A, LATS2, EEF1D, ZMYND11, KDM7A, HLF, USP12, TBRG4, RMND5A, TNKS2, STOM, MAPK6, TAF10, ATP2B1, WEE1, CDK2AP2, RBM42* |
| TBX5 | 170.24 | -0.90 | Inhibited | 25 | 1.67E-04 | 1.83E-03 | *SMC2, KRTCAP2, PSMD4, NAMPT, VPS18, AARSD1, RNF125, RPL28, NPRL2, RPN1, TOE1, MMS19, MTMR10, ABCC10, EEA1, GMIP, TUT1, PPARA, AHNAK, DCBLD2, GATM, LAMTOR2, MAP2K3, FKBP9, IP6K2, NEK9, EIF2B4, NGRN, SORD, REXO4, DAD1, SNRPA1, TSSC4, HM13, CXXC1, POLR2I, POLR2J, SEC11C, OXR1, GFER, NAGK, SLC25A30, SNRPD1, SOX6, CPT1A, ACTN2, ALG5, PREB, SNX4, CALM3, FKBP11, YAP1, NR3C1, EME2, KNTC1, CHCHD1, LMO7, ACYP2, COBLL1, ECT2, ACBD5, TJAP1, DLG1, NABP2, CHD9, YWHAG, ARHGEF10, KDM7A, HLF, PSMB10, CLN5, CMTM4, SLC50A1* |
| SOX2 | 189.76 | -89.41 | Inhibited | 26 | 1.98E-04 | 1.89E-03 | *KIAA1109, NAMPT, BIN3, DDIT3, PRKD2, CDCA2, SDR42E1, NEBL, NR0B2, VANGL1, MTMR10, MRPL38, PYGL, PBK, ANXA4, ANXA5, GPRC5A, CDC42EP5, PPARA, RAPGEF5, AHNAK, PDF, CHN2, LAPTM5, DCBLD2, SMOC2, AADAC, HSPA1A, PLOD2, PYCARD, ATP11C, CNPY2, CLIP1, UTRN, IP6K2, CYBRD1, ABHD18, SERINC5, BMPR2, LSP1, PCCA, MET, GLRX, SH3BP2, FNIP1, CYB5A, CDC42BPA, KBTBD11, TXNDC16, EFR3A, FBXO3, TNS3, PDK1, NEIL2, PLPP3, CFTR, HLTF, STK38L, ASL, CD14, FKBP11, SLC44A4, ROBO1, RPUSD4, TOR4A, SATB1, KIAA1324L, GEMIN2, NR3C1, ARHGAP42, EME2, FAM3A, HIST1H2AC, ALDH3A2, CCNE2, DPP4, COBLL1, CRIM1, HSPA2, GCLM, BRWD1, ARHGEF10, ETV5, TRAPPC6A, LATS2, ALDH1A1, KDM7A, USP12, PRRG4, TMEM30B, ATP2B1* |
| FOXO3 | 164.43 | 60.35 | Activated | 28 | 2.30E-04 | 2.08E-03 | *SMC2, MPV17, AARSD1, HDHD5, CLPP, FDX2, RPL28, NPRL2, MRPL16, NR1D2, MTMR10, PQBP1, GSTO1, PLCB1, SQOR, PSMB8, GATM, SMCHD1, SNRNP25, ENY2, EIF2B4, GOT1, TMEM11, ZC3H3, NGRN, PSTPIP1, SGCB, REXO4, TSSC4, CXXC1, NCLN, POLR2G, POLR2I, POLR2J, SLC35A3, DDHD2, OXR1, FBXL12, NUBP1, MRPS18A, PREB, STK38L, STK11IP, LACC1, CALM3, CUTC, SUGP1, RAC2, MICU3, CD47, TYMS, NPM3, FAM3A, ALG12, ALDH3A2, TMEM258, SELENOP, SHARPIN, RHBDD3, KLHL26, HOOK3, YWHAG, TSFM, RABEP2, ACOX1, EEF1D, KDM7A, CLN5, TBRG4, TNKS2, SLC50A1, WEE1* |
| RUNX1 | 165.97 | 11.65 | Activated | 27 | 2.27E-04 | 2.08E-03 | *SPI1, GMFG, KRTCAP2, CDK5RAP3, IER2, HDHD5, UBA52, EXD2, NPRL2, RPN1, MRPL16, WDR70, NR2C2AP, TOE1, IRF2BP2, RMDN2, FOSL2, NFATC4, ADI1, ABCC10, ING1, CYB561D2, EEA1, ANXA4, ANXA5, SUMF1, KAT2B, HEATR5A, AEBP2, SF3B5, APOLD1, RNF138, CHN2, LAPTM5, TARBP2, SNX15, LAMTOR2, LAMTOR5, CHPF, LTN1, GOLIM4, MYLK4, STK16, MAP2K3, ZBTB39, TPM1, CNPY3, UTRN, SNRPA, ENY2, INO80E, FECH, INSIG2, UBR3, FOXO4, ATXN7, AMDHD2, MAP3K20, ANKRD29, EIF2B4, FOXN2, BFSP1, TMEM11, BCL3, MDM2, ZNF771, BMPR2, ZC3H3, CELF4, PIK3CA, DAD1, SNRPA1, TSSC4, GTF3C5, CXXC1, GLRX, MRM1, HECTD1, NCLN, SH3BP2, POLR2I, SPAG9, PTPN18, FOXJ3, DDHD2, PTPN12, OXR1, GFER, ATOX1, KLF6, KLF9, TRMT61A, SMARCB1, JMJD1C, NUBP1, NUBP2, EFR3A, ZFPL1, TNS3, CPT1A, MRPS18A, ALG3, IMP4, NEIL1, STXBP3, FXR1, PTDSS2, STK38L, SYMPK, STXBP5, CD14, STK11IP, CALM3, MRPS11, CITED4, IDE, GUK1, RAC2, ACADM, TMED9, VAV1, CD47, C1QB, PTGER3, NT5C, PES1, NPM3, WASHC4, NR3C1, SRP14, EME2, SBF2, CHCHD1, ZGPAT, DPM2, DOK2, TYROBP, RPS19BP1, CBX8, TCAF1, ARHGEF37, GCLM, BRWD1, COPE, PICALM, CHD9, TNFAIP3, PPP1R9B, ADGRE5, ENOSF1, YWHAG, TSFM, YWHAZ, ETV5, LATS2, CAT, PNKP, CLN5, TBRG4, MVB12A, STOM, MAPK6, TAF10, SLC50A1, WEE1, BUD13* |
| TCF7L2 | 160.52 | -114.66 | Inhibited | 29 | 4.93E-04 | 4.09E-03 | *EXD2, BLOC1S6, WDR70, VANGL1, AEBP2, PLCB1, FADS2, CDH2, CLIP1, ENY2, FECH, EIF2B4, CCDC22, SORD, JMJD1C, EIF2D, SOX6, CPT1A, B4GALNT1, CUTC, IDE, ROBO1, GYS2, SH3BGRL, IL33, LMO7, ACBD5, CRIM1, PPP1R9B* |
| RUNX2 | 192.40 | -26.39 | Inhibited | 30 | 5.80E-04 | 4.57E-03 | *NCKAP1, KRTCAP2, MYC, IER2, CHID1, HDHD5, BIN1, CDCA2, ABCB8, PRKAR2A, IRF2BP2, FOSL2, NFATC4, ADI1, MTMR10, ING1, TRMT112, GSTO1, ANXA5, KAT2B, GPRC5A, AEBP2, AHNAK, CHN2, BID, LAPTM5, TARBP2, SLMAP, FADS2, SMCHD1, MAP2K3, TPM1, CLIP1, UTRN, AMDHD2, MDM2, LSP1, GTF3C5, HM13, GLRX, MRM1, POLR2J, PTPN12, CDC42BPA, KBTBD11, POLR3H, SOX6, TNS3, PDK1, MRPS18A, VAT1, MYO5C, CUTC, FKBP11, SLC44A4, SH3PXD2B, RAC2, KIF2C, GPRIN3, COX19, ECT2, TJAP1, NABP2, BRWD1, PPP1R9B, YWHAG, YWHAZ, KDM7A, LUM, WEE1* |
| FOXA1 | 193.76 | -119.31 | Inhibited | 31 | 5.96E-04 | 4.58E-03 | *SLC4A4, MYC, RNF125, CDCA2, NEBL, MCAT, FOSL2, ADI1, PAH, ING1, PCMTD1, SUMF1, ANGPTL3, PLCB1, RAPGEF5, AHNAK, FAM214A, RNF138, CHN2, SNX15, PLOD2, SMCHD1, ATP11C, SLC9A2, INSIG2, ANKRD29, DHCR24, TMEM11, CPD, ALB, SLCO1A2, MDM2, PTPN21, SGCB, PIK3CA, UNC93B1, HM13, TP53INP1, FOXJ3, KBTBD11, KLF6, SLC25A30, TRMT61A, ECH1, PLPP3, RACGAP1, STXBP5, APOB, ZFX, ZDHHC17, CD47, IL33, SBF2, HIST1H2AC, BRMS1, CCNE2, SELENOP, PPIH, COBLL1, DHRS7B, CBX8, ACBD5, ARHGEF37, DENND4A, RABEP2, KDM7A, PFDN6, MAPK6, CMTM4, TAF10, WEE1, CDK2AP2, SNTB1* |
| FLI1 | 180.18 | 0.99 | Activated | 32 | 6.61E-04 | 4.95E-03 | *NCKAP1, SPI1, GMFG, WDR83, SMC2, KIAA1109, KRTCAP2, SCP2, PSMD4, LIPH, MPV17, CDK5RAP3, IER2, VPS18, RNF125, BIN3, BIN1, CLPP, DDIT3, PRKD2, UBA52, RPL28, EXD2, NPRL2, RPN1, MRPL16, BLOC1S6, WDR70, TOE1, NEBL, RMDN2, MCAT, FOSL2, ADI1, ABCC10, ING1, CYB561D2, PQBP1, EEA1, TRMT112, PCMTD1, GSTO1, ANXA4, RNF167, HEATR5A, RAPGEF5, AHNAK, APOLD1, PDF, CHN2, SC5D, BID, LAPTM5, AADAC, ZNF576, LAMTOR5, CHPF, LTN1, GOLIM4, MSI1, FADS2, STK16, NUDT12, MAP2K3, LENG1, ENY2, INO80E, CALCRL, INSIG2, AMDHD1, EIF2B4, FOXN2, BFSP1, TMEM11, CCDC22, COPS9, ISY1, ZC3H3, NGRN, GPX1, REXO4, PIK3CA, PCCA, DAD1, SNRPA1, TSSC4, GTF3C5, ROCK2, HM13, CXXC1, SH3BP2, POLR2G, POLR2I, PTPN18, FOXJ3, PTPN12, CDC42BPA, KBTBD11, KLF6, KLF5, NAGK, KLF9, TP53RK, NUBP1, NUBP2, SNRPD1, ZFPL1, TNS3, CPT1A, ALG3, IMP4, NEIL2, PREB, NEIL1, STXBP3, PTDSS2, RACGAP1, MAP7, STK38L, SYMPK, STXBP5, CD14, ZRANB1, STK11IP, MYO5C, NDUFAF3, CALM3, IDE, GUK1, SH3PXD2B, RAC2, PRMT6, TMED9, VAV1, NME1, APRT, KIF2C, CD47, SLU7, AMIGO1, SCAND1, WASHC4, EME2, ACYP2, DPM2, TYROBP, GPRIN1, PPIH, RPS19BP1, CCNB1, DHRS7B, CBX8, ACBD5, DENND4A, NONO, CRIM1, ARHGAP25, NABP2, BRWD1, COPE, PICALM, CHD9, TNFAIP3, PPP1R9B, YWHAG, PEX1, YWHAZ, ETV5, RABEP2, DOCK1, PNKP, USP12, PSMB10, CLN5, TBRG4, MVB12A, STOM, CMTM4, TAF10, SLC50A1, WEE1, CDK2AP2, BUD13* |
| FOXP3 | 152.33 | 28.88 | Activated | 33 | 6.83E-04 | 4.99E-03 | *WDR83, ZC3H12D, SCP2, CDK5RAP3, IER2, MRPL16, BLOC1S6, FOSL2, PQBP1, MCEE, SUMF1, HEATR5A, TUT1, APOLD1, SC5D, TARBP2, LAMTOR2, ZNF576, LAMTOR5, ENY2, EIF2B4, GOT1, CDK1, MDM2, LSP1, REXO4, SNRPA1, GTF3C5, OXR1, EIF2D, ECH1, ALG3, PPP1R37, PTDSS2, SYMPK, MRPS11, SEC61B, APRT, PES1, YIPF1, KNTC1, SHARPIN, ECT2, ANKZF1, RHBDD3, DENND4A, ARHGAP25, DDX41, PPP1R9B, TSFM, TRAPPC6A, EEF1D, USP12, RMND5A, BUD13* |
| ETS1 | 149.09 | 89.80 | Activated | 34 | 8.12E-04 | 5.54E-03 | *KRTCAP2, PSMD4, IER2, CLPP, UBA52, NPRL2, MCTS1, TRMT112, MRPL42, TUT1, SF3B5, RIDA, PSMB2, TARBP2, SLMAP, LAMTOR2, ZNF576, LAMTOR5, LTN1, MAP2K3, CNPY2, INO80E, FBXO48, EIF2B4, TMEM11, CCDC22, ISY1, REXO4, COASY, SNRPA1, TSSC4, CXXC1, NCLN, SEC11C, PANK3, SNRPD1, MRPS18B, IMP4, PTDSS2, RACGAP1, SYMPK, STXBP5, STK11IP, MRPS11, PRMT6, TMED9, NME1, PES1, SCAND1, DPM2, BRMS1, DHRS7B, ACBD5, COPE, DDX41, YWHAG, TSFM, PFDN6, TBRG4, MVB12A, RBM42* |
| STAT5A | 198.11 | -7.48 | Inhibited | 35 | 8.33E-04 | 5.56E-03 | *SMC4, PSMD4, NAMPT, TTPA, FERMT1, MTMR10, PBK, FMO2, PSMB8, LAMTOR2, PYCARD, ZBTB39, MDM2, GPX1, REXO4, SLC35A3, KLF6, NAGK, KLF9, SLC25A30, SLC25A35, PANK3, ACTN2, CD14, EAF2, CALM3, FKBP11, INTS11, CITED4, PRMT6, CRIP3, IL33, KNTC1, ALDH3A2, GPRIN3, SELENOP, ANKZF1, PICALM, CPTP, PSMB10, TNKS2, SLC50A1, SNTB1* |
| TET1 | 201.44 | 18.81 | Activated | 36 | 8.74E-04 | 5.71E-03 | *GMFG, WDR83, SMC4, ZC3H12D, SCP2, RNF125, DDIT3, FDX2, NPRL2, ANAPC11, ING1, PCMTD1, CDC42EP5, AHNAK, CHPF, MSI1, STX7, CNPY3, SNRNP25, SNRPA, FBXO48, IP6K2, GOT1, CYBRD1, SORD, MET, UNC93B1, HM13, ZBTB44, KBTBD11, ATOX1, KLF6, POLR3H, NUBP2, CPT1A, ALG3, CD14, EAF2, MYO5C, FKBP11, SLC44A4, GYS2, SH3PXD2B, C1QB, UQCR11, AIF1, NT5C, PES1, RTL6, BRMS1, CCNE2, SELENOP, COX19, RPS19BP1, SHARPIN, CBX8, TCAF1, RHBDD3, DDX41, ARHGEF10, ZMYND11, CDK2AP2* |
| RELA | 189.96 | -58.55 | Inhibited | 38 | 9.31E-04 | 5.74E-03 | *SPI1, RAPH1, MYC, NAMPT, IER2, BIN3, NPRL2, IRF2BP2, FOSL2, CYB561D2, PQBP1, PCMTD1, GPRC5A, CDC42EP5, AHNAK, HSPA1A, PYCARD, SMCHD1, MAP2K3, TPM1, UTRN, FECH, BFSP1, CPD, BCL3, PIK3CA, GLRX, SPAG9, CYB5A, PTPN12, ATOX1, KLF5, EIF4A2, MAP7, CD209, ZFX, CD48, SH3BGRL, SLC31A1, NR3C1, CYBA, DPP4, DHRS7B, ECT2, DENND4A, GCLM, TNFAIP3, TBRG4, PRRG4* |
| SMAD4 | 172.22 | -142.30 | Inhibited | 39 | 9.35E-04 | 5.74E-03 | *IRF2BP2, MMS19, ANXA4, GPRC5A, INSIG2, NEK9, RAD9A, PTPN21, SPAG9, CDC42BPA, KLF6, KLF5, JMJD1C, MAP7, CALM3, ECT2, ARHGEF37, DENND4A, PICALM, CHD9, ARHGEF10* |
| VDR | 148.40 | 43.26 | Activated | 37 | 9.09E-04 | 5.74E-03 | *NCKAP1, POP1, SMC2, SCP2, CDK5RAP3, IER2, RNF125, RUFY3, CLPP, DDIT3, UBA52, CDCA2, NPRL2, MRPL16, ABCB8, MRPL38, ING1, PCMTD1, TUT1, SF3B5, FAM214A, RNF138, TARBP2, ZNF576, LTN1, SMCHD1, CNPY3, FECH, RLF, AMDHD2, FOXN2, MDM2, ZNF414, ISY1, ZC3H3, HM13, HSD17B11, NCLN, POLR2J, NAGK, POLR3H, EIF4A2, JMJD1C, EFR3A, SNRPD1, RPL36AL, EIF2D, ZFPL1, ALG5, ECH1, IMP4, PREB, STXBP3, RACGAP1, SYMPK, CALM3, MRPS11, RAC2, SEC61B, NME1, NEDD8, PES1, SCAND1, HIST1H2AC, ACYP2, DOK2, PPIH, RPS19BP1, DDX41, ENOSF1, TSFM, KDM7A, PFDN6, PSMB10, CLN5, TBRG4, RMND5A, RBM42, BUD13* |
| ELK3 | 174.81 | -34.54 | Inhibited | 40 | 1.24E-03 | 7.19E-03 | *PSMD4, IER2, RNF125, BIN1, PRKD2, MRPL16, IRF2BP2, FOSL2, NFATC4, ADI1, ABCC10, ING1, TRMT112, NUDC, GPRC5A, AHNAK, LAPTM5, LAMTOR2, CHPF, FADS2, TPM1, UTRN, AMDHD1, BCL3, BMPR2, PSTPIP1, TSSC4, SH3BP2, FNIP1, SLC35A3, PTPN12, OXR1, KLF6, KLF9, CPT1A, NEIL1, STXBP3, PTDSS2, APOB, VAT1, CALM3, CUTC, GUK1, STBD1, SH3PXD2B, RAC2, ACADM, PRMT6, ZFX, YIPF1, ACYP2, DOK2, BRMS1, GPRIN1, PPIH, RPS19BP1, ECT2, NONO, CRIM1, DLG1, PICALM, TNFAIP3, PPP1R9B, YWHAZ, TRAPPC6A, CPTP, DOCK1, PSMB10, TNKS2, SLC50A1, WEE1, CDK2AP2* |
| EOMES | 191.13 | -113.44 | Inhibited | 42 | 1.37E-03 | 7.65E-03 | *NCKAP1, SMC4, BIN1, EXD2, IRF2BP2, VANGL1, PYGL, AHNAK, CHN2, GATM, LAMTOR2, MSI1, PLOD2, FADS2, TPM1, ATP11C, CNPY3, TMEM11, CPD, PCCA, CYB5A, SLC35A3, SLC25A35, JMJD1C, STXBP3, VAT1, CUTC, IDE, ACADM, CD47, YIPF1, NPM3, ARHGAP42, IL11RA, ALDH3A2, DENND4A, GCLM, PICALM, STOM* |
| ZNF217 | 198.46 | -166.21 | Inhibited | 41 | 1.35E-03 | 7.65E-03 | *RAPH1, MYC, LIPH, WDR70, NEBL, IRF2BP2, FOSL2, VANGL1, ANXA4, KAT2B, PLCB1, RAPGEF5, AHNAK, FAM214A, CHN2, RNLS, PLOD2, NUDT12, TPM1, SLC9A2, UTRN, INSIG2, FOXN2, CDK1, SERINC5, DAD1, MET, HM13, TP53INP1, FOXJ3, PTPN12, OXR1, KLF6, KLF5, SOX6, TNS3, CPT1A, PLPP3, CFTR, MYO5C, CITED4, ROBO1, SH3PXD2B, SEC61B, CD47, SATB1, ITCH, NR3C1, SBF2, COBLL1, CRIM1, DLG5, YWHAZ, ZMYND11, USP12, MAPK6, SNTB1* |
| GATA4 | 184.80 | -87.40 | Inhibited | 43 | 1.53E-03 | 8.37E-03 | *NCKAP1, SLC4A4, PSMD4, RUFY3, ABCB8, NEBL, FOSL2, HRG, MTMR10, SUMF1, AEBP2, TUT1, SF3B5, PSMB2, GOLIM4, PYCARD, UTRN, FECH, AMDHD1, AMDHD2, IP6K2, GOT1, CYBRD1, PTPN21, SORD, REXO4, ROCK2, PTPN12, OXR1, BICC1, NAGK, SOX6, CPT1A, PLPP3, PTDSS2, RACGAP1, HLTF, SYMPK, B4GALNT1, ZRANB1, CALM3, CUTC, RAC2, KIF2C, YAP1, TYMS, NR3C1, KNTC1, CHCHD1, LMO7, CCNE2, COX19, RHBDD3, NABP2, PICALM, DDX41, YWHAG, YWHAZ, ETV5, PFDN6, WEE1* |
| DACH1 | 168.37 | -4.09 | Inhibited | 44 | 1.72E-03 | 8.97E-03 | *RPL28, IRF2BP2, ADI1, MMS19, TARBP2, DCBLD2, ZNF576, CHPF, FADS2, ZBTB39, FKBP9, AMDHD2, EIF2B4, CDK1, RAD9A, ZC3H3, PTPN21, TSSC4, ROCK2, HM13, SPAG9, BICC1, EIF4A2, PANK3, SNRPD1, RPL36AL, ZFPL1, PDK1, ECH1, MRPS18B, ALG3, PPP1R37, FXR1, PTDSS2, CALM3, IDE, ACADM, PRMT6, CD47, YAP1, NT5C, WASHC4, EME2, ZGPAT, ALDH3A2, RPP21, SHARPIN, ACBD5, CRIM1, CHD9, PPP1R9B, YWHAG, EEF1D, KDM7A, USP12, NR2C2, TBRG4, TAF10, CDK2AP2, BUD13* |
| SOX17 | 193.37 | -56.69 | Inhibited | 45 | 1.81E-03 | 9.24E-03 | *LIPH, IER2, RUFY3, VANGL1, HRG, ING1, EEA1, PCMTD1, GSTO1, ANXA4, TRMT10B, SLMAP, STK16, NUDT12, MAP2K3, ZBTB39, UTRN, IP6K2, TMEM11, SERINC5, SORD, HM13, CXXC1, ZBTB44, NCLN, POLR2I, KCNJ2, SEC11C, ATOX1, KLF6, SOX6, ECH1, GOLPH3L, ALG3, IMP4, NEIL2, PLPP3, SNX4, ZRANB1, SUGP1, ROBO2, ROBO1, SH3PXD2B, ACADM, NME1, CD47, PES1, SCAND1, SLC31A1, KLB, KNTC1, ALG12, BRMS1, SELENOP, ECT2, BRWD1, GMPPA, YWHAZ, ACOX1, NR2C2, PRRG4* |
| ELK1 | 168.70 | -26.27 | Inhibited | 46 | 2.18E-03 | 1.08E-02 | *NCKAP1, POP1, MYC, IER2, WDR70, MCTS1, TUT1, SF3B5, AHNAK, GOLIM4, NUDT12, TPM1, G6PC3, ISY1, SNRPA1, MET, ZBTB44, POLR2G, PTPN12, KLF6, NAGK, EIF4A2, TP53RK, FBXO3, ECH1, MRPS18B, RACGAP1, STK11IP, NME1, NPM3, KNTC1, ACYP2, DLG1, YWHAZ, NR2C2, TNKS2, SLC50A1* |
| FOXM1 | 245.72 | -82.93 | Inhibited | 47 | 2.22E-03 | 1.08E-02 | *SMC4, CDCA2, VANGL1, FERMT1, PBK, APOLD1, LENG1, IP6K2, GOT1, FOXN2, CDK1, MDM2, FAM207A, GTF3C5, HSD17B11, KLF6, NAGK, POLR3H, ZFPL1, MRPS18B, PPP1R37, RACGAP1, CALM3, KIF2C, YAP1, BRMS1, COX19, CCNB1, SHARPIN, COBLL1, ECT2, NABP2, CFAP43, PEX1, ETV5, CAT, WEE1, CDK2AP2* |
| SOX9 | 173.99 | -53.08 | Inhibited | 48 | 2.44E-03 | 1.17E-02 | *NAMPT, BIN3, CLPP, TTPA, SDR42E1, FOSL2, VANGL1, MRPL42, PCMTD1, ANXA5, PLCB1, AHNAK, LAPTM5, BBOX1, HSPA1A, GOLIM4, IL1RAP, PYCARD, UTRN, SNRPA, TMEM11, GPX1, TSSC4, POLR2I, ATOX1, KLF6, KLF9, EIF4A2, TNS3, SYMPK, MRPS11, SH3PXD2B, NME1, IL33, ARHGAP42, SBF2, LMO7, CRIM1, TJAP1, GCLM, CHD9, PPP1R9B, CNPPD1, ETV5, LATS2, ALDH1A1, DOCK1, CLN5, MAPK6, TAF10, ATP2B1* |
| FOXP1 | 155.66 | 6.78 | Activated | 49 | 3.18E-03 | 1.50E-02 | *RLF, HM13, RPL36AL, ACYP2* |
| KLF4 | 202.70 | -80.17 | Inhibited | 50 | 3.72E-03 | 1.70E-02 | *MYC, IER2, RNF125, CLPP, UBA52, NPRL2, ABCB8, FOSL2, NFATC4, MCEE, PCMTD1, KAT2B, RNF167, GPRC5A, AEBP2, CDC42EP5, PYCARD, G6PC3, BCL3, GPX1, TSSC4, HSD17B11, CYB5A, ATOX1, KLF6, KLF5, NAGK, KLF9, EIF4A2, ECH1, MRPS18B, ALG3, PLPP3, VAT1, MYO5C, ROBO2, ACADM, PES1, ALDH3A2, CCNE2, CCNB1, ECT2, CBX8, BRWD1, PICALM, YWHAG, YWHAZ, LATS2, ZMYND11, CMTM4, WEE1* |
| YY1 | 151.40 | -22.37 | Inhibited | 51 | 3.76E-03 | 1.70E-02 | *SMC4, SCP2, FDX2, UBA52, EXD2, ABCB8, MMS19, MTMR10, MRPL38, MRPL42, SUMF1, HEATR5A, PLCB1, AHNAK, KIDINS220, SLMAP, UHRF1BP1L, MYLK4, UBR3, TMEM11, MET, CXXC1, METAP1D, SMARCB1, JMJD1C, ECH1, MRPS18B, MRPS18A, ALG3, IMP4, PREB, FXR1, SNX4, RACGAP1, GYS2, TMED9, ZDHHC17, RPUSD4, ACYP2, ALG12, RHBDD3, TJAP1, CHD9, DDX41, PEX1, PFDN6, TBRG4, TNKS2, LUM, ATP2B1* |
| POU5F1 | 188.17 | -19.78 | Inhibited | 52 | 4.66E-03 | 2.02E-02 | *RAPH1, KRTCAP2, MYC, LIPH, NAMPT, IER2, BHLHA15, RNF125, CLPP, DDIT3, CDCA2, ABCB8, CFP, WDR70, TTPA, NFATC4, VANGL1, MRPL38, MCEE, PBK, KAT2B, AEBP2, TUT1, RAPGEF5, SF3B5, AHNAK, TARBP2, SLMAP, HSPA1A, SMCHD1, ATCAY, MAP2K3, SLC9A2, UTRN, ENY2, NEK9, EIF2B4, BCL3, SERINC5, ISY1, ZC3H3, PTPN21, MRPL4, GPX1, REXO4, EVC, PCCA, GTF3C5, ROCK2, HM13, ZBTB44, HSD17B11, MRM1, POLR2J, SPAG9, PTPN18, GFER, KLF6, KLF5, KLF9, EIF4A2, EIF2D, ZFPL1, TNS3, PDK1, CPT1A, MRPS18B, FNDC4, PLPP3, SNX4, RACGAP1, HLTF, STK38L, CD14, SUGP1, SLC44A4, TMED9, NME1, RPUSD4, KIF2C, CD47, SATB1, UQCR11, NEDD8, AIF1, GEMIN2, SRP14, DOK2, CCNE2, GPRIN1, SELENOP, PPIH, RPS19BP1, KLHL13, DPP4, DHRS7B, ECT2, CBX8, KLHL26, HSPA2, DLG1, NABP2, BRWD1, CHD9, PPP1R9B, ADGRE5, GMPPA, YWHAG, ARHGEF10, YWHAZ, ETV5, LATS2, RABEP2, PRRG4, WEE1, BUD13* |
| WT1 | 207.75 | -116.52 | Inhibited | 53 | 4.68E-03 | 2.02E-02 | *NCKAP1, SCP2, WDR91, VPS18, RPN1, NEBL, IRF2BP2, RMDN2, FOSL2, NFATC4, VANGL1, PAH, MMS19, MYOM1, PYGL, MCEE, PCMTD1, GSTO1, ANXA4, KAT2B, GPRC5A, HEATR5A, AEBP2, RAPGEF5, CHN2, LAPTM5, BBOX1, DCBLD2, SMOC2, GOLIM4, MSI1, IL1RAP, PYCARD, FADS2, NUDT12, TPM1, STX7, CLIP1, UTRN, INSIG2, ILDR2, GOT1, FOXN2, CPD, BCL3, SERINC5, BMPR2, PTPN21, LSP1, PCCA, DAD1, ROCK2, HM13, MRM1, SH3BP2, KCNJ2, CYB5A, PTPN12, CDC42BPA, BICC1, KLF6, SLC25A30, TXNDC16, SOX6, TNS3, PDK1, NEIL2, PREB, PLPP3, FXR1, MAP7, STXBP5, PCK1, APOB, LACC1, MYO5C, ROBO2, GRIK5, ROBO1, SH3PXD2B, RAC2, IL33, YIPF1, NPM3, RTL6, SSC4D, NR3C1, SBF2, SELENOP, COBLL1, CBX8, ARHGEF37, CRIM1, DLG5, BRWD1, CHD9, PPP1R9B, GMPPA, ARHGEF10, YWHAZ, LATS2, USP12, STOM, MAPK12, WEE1, SNTB1* |
| KLF5 | 191.76 | -79.40 | Inhibited | 54 | 5.66E-03 | 2.34E-02 | *SMC2, CDK5RAP3, IER2, BIN1, DDIT3, EXD2, RPN1, IRF2BP2, MCAT, FOSL2, VANGL1, FERMT1, MRPL38, EEA1, ANXA4, HEATR5A, SF3B5, AHNAK, SMOC2, ZNF576, NUDT12, SLC9A2, ATXN7, FOXN2, BCL3, MDM2, BMPR2, PIK3CA, PCCA, ROCK2, POLR2J, KCNJ2, SPAG9, METAP1D, CDC42BPA, OXR1, NAGK, KLF9, TNS3, CPT1A, MRPS18A, STXBP3, FXR1, ALDH6A1, CALM3, ROBO2, CD47, SATB1, NT5C, ITCH, SSC4D, NR3C1, ARHGAP42, KNTC1, LMO7, ECT2, KLHL26, PICALM, YWHAZ, LATS2, RABEP2, HLF, CLN5, LUM, SLC50A1, CDK2AP2* |
| NANOG | 188.63 | -61.49 | Inhibited | 55 | 5.85E-03 | 2.36E-02 | *POP1, MYC, HDHD5, RNF125, CDCA2, NPRL2, PRKAR2A, TTPA, NFATC4, PAH, PYGL, CYB561D2, PQBP1, EEA1, ANXA5, KAT2B, AEBP2, RNF138, CHN2, TRMT10B, SMOC2, LAMTOR2, FADS2, STK16, MAP2K3, TPM1, CNPY3, UTRN, FOXO4, FOXN2, MDM2, ZC3H3, GPX1, HM13, ZBTB44, HSD17B11, POLR2I, SEC11C, CYB5A, PTPN12, OXR1, KLF6, KLF5, NAGK, KLF9, EIF4A2, PANK3, SMARCB1, FBXO3, SOX6, PLPP3, NEIL1, HLTF, RAC2, RPUSD4, AIF1, ZGPAT, ACYP2, BRMS1, GPRIN1, PPIH, KLHL13, NONO, HOOK3, BRWD1, ETV5, LATS2, RABEP2, EEF1D, MAPK6* |
| AR | 186.52 | -155.34 | Inhibited | 59 | 6.39E-03 | 2.45E-02 | *NCKAP1, SLC4A4, SMC2, CLPP, WDR70, NEBL, RMDN2, PCMTD1, ANXA5, TUT1, RAPGEF5, AHNAK, CHN2, SC5D, RNLS, SQOR, BBOX1, LTN1, IL1RAP, NUDT12, STX7, SLC9A2, UTRN, INSIG2, UGT3A2, ANKRD29, FOXN2, TMEM11, SLCO1A2, SERINC5, BMPR2, PTPN21, PIK3CA, KCNJ2, CYB5A, PTPN12, KLF6, TXNDC16, TNS3, PLPP3, SH3PXD2B, PRMT6, YAP1, HSD17B6, SH3BGRL, NR3C1, ARHGAP42, SBF2, LMO7, GPRIN3, DPP4, COBLL1, CRIM1, HOOK3, DLG5, PICALM, CHD9, DOCK7, TNFAIP3, ALDH1A1, DOCK1, STOM, MAPK6* |
| PRDM5 | 163.96 | -112.43 | Inhibited | 58 | 6.35E-03 | 2.45E-02 | *SMC2, NAMPT, CDCA2, PBK, PCMTD1, RIDA, TRMT10B, ATP11C, UTRN, ENY2, ZC3H3, REXO4, MET, TP53INP1, DDHD2, PTPN12, OXR1, EFR3A, TNS3, CFTR, MAP7, STXBP5, STAG2, SEC61B, ZFX, SH3BGRL, FAM3A, KIAA2026, KLHL13, NONO, HOOK3, TNFAIP3, YWHAG, ARHGEF10, PEX1, ALDH1A1, TBRG4, MARVELD2, SNTB1* |
| SETDB1 | 191.11 | 18.93 | Activated | 57 | 6.29E-03 | 2.45E-02 | *KRTCAP2, MYC, CDK5RAP3, BIN1, DDIT3, ABCB8, ANAPC11, MCAT, NR0B2, ABCC10, GSTO1, RNF167, AEBP2, AHNAK, APOLD1, TARBP2, MSI1, PLOD2, SMCHD1, ATCAY, MAP2K3, RAD9A, BMPR2, COASY, GTF3C5, HM13, CXXC1, GLRX, ZBTB44, PTPN18, OXR1, ATOX1, FBXL12, KLF9, SOX6, TNS3, B4GALNT1, VAT1, CALM3, GUK1, ACADM, SEC61B, PRMT6, TMED9, CD47, CRIP3, GEMIN2, NR3C1, CYBA, RPS19BP1, COBLL1, ACBD5, DLG1, CHD9, GMPPA, YWHAZ, CAT, DOCK1, PFDN6, USP12, PSMB10* |
| TAL1 | 187.57 | -32.13 | Inhibited | 56 | 6.22E-03 | 2.45E-02 | *SMC2, MYC, CDK5RAP3, IER2, VPS18, BIN3, DDIT3, RPN1, NR1D2, ING1, KAT2B, SF3B5, AHNAK, TARBP2, SNX15, UHRF1BP1L, IL1RAP, MAP2K3, TPM1, UTRN, FECH, FBXO48, ZC3H3, GTF3C5, GLRX, HECTD1, REC8, KLF6, KLF5, KLF9, POLR3H, NUBP2, FBXO3, SOX6, TNS3, PDK1, CPT1A, PPP1R37, PTDSS2, STK38L, B4GALNT1, PCK1, VAT1, MYO5C, CITED4, RAC2, CD47, PTGER3, HIST1H2AC, SHARPIN, ARHGAP25, DLG1, GCLM, DDX41, PPP1R9B, ADGRE5, CAT, KDM7A, USP12, MAPK6, ATP2B1* |
| SMAD3 | 195.15 | -74.20 | Inhibited | 60 | 7.07E-03 | 2.68E-02 | *RAPH1, SMC2, MYC, NAMPT, VPS18, ABCB8, NEBL, IRF2BP2, FOSL2, ANXA4, GPRC5A, TUT1, AHNAK, FAM214A, SQOR, LAPTM5, BBOX1, GOLIM4, PLOD2, FADS2, MAP2K3, CLIP1, UTRN, INSIG2, BFSP1, MDM2, RAD9A, ISY1, BMPR2, PTPN21, MET, GTF3C5, HECTD1, NCLN, POLR2J, SPAG9, KLF6, EIF4A2, JMJD1C, ECH1, MRPS18B, ALG3, CALM3, IDE, GUK1, SEC61B, NME1, YAP1, NPM3, COBLL1, ARHGEF37, DENND4A, CRIM1, GCLM, PICALM, CHD9, ARHGEF10, YWHAZ, ACOX1, PRRG4, STOM, MAPK6, SLC50A1, WEE1* |
| SREBF2 | 163.83 | -10.68 | Inhibited | 61 | 7.48E-03 | 2.80E-02 | *GMFG, WDR91, IER2, RPL28, PRKAR2A, TTPA, ING1, PCMTD1, GMIP, PPARA, SQOR, SNX15, ENY2, UBR3, PSTPIP1, REXO4, MET, KLF6, KLF9, TRMT61A, NUBP2, EFR3A, SNRPD1, TNS3, CPT1A, YAP1, C1QB, DPM2, BRMS1, CCNE2, RHBDD3, TJAP1, NABP2, DDX41, YWHAG, ZMYND11* |
| SALL4 | 229.95 | -176.58 | Inhibited | 62 | 8.56E-03 | 3.13E-02 | *LIPH, RNF125, RPL28, CFP, GPRC5A, AEBP2, CDC42EP5, FADS2, ATCAY, FOXN2, CPD, HSD17B11, PTPN12, KLF5, KLF9, EIF4A2, PDK1, PLPP3, HLTF, STK38L, VAT1, CUTC, GRIK5, SATB1, CCNE2, ECT2, GCLM, BRWD1, CHD9, YWHAG, ETV5, LATS2, HLF, MAPK6, WEE1* |
| CUX1 | 177.55 | -14.85 | Inhibited | 63 | 9.61E-03 | 3.41E-02 | *POP1, RAPH1, SMC4, SMC2, SCP2, MYC, IER2, HDHD5, FDX2, RPL28, ABCB8, BLOC1S6, MCTS1, IRF2BP2, TYSND1, VANGL1, HRG, MTMR10, PQBP1, MRPL42, PBK, ANXA5, TUT1, PLCB1, APOLD1, RIDA, PSMB2, LAPTM5, ZNF576, MPV17L2, FADS2, CDH2, LENG1, FECH, UBR3, BFSP1, BCL3, ISY1, LSP1, REXO4, COASY, EVC, DAD1, GTF3C5, UNC93B1, CXXC1, GLRX, ATOX1, KLF9, EIF4A2, JMJD1C, NUBP1, PDK1, MRPS18A, ALG3, IMP4, STXBP3, RACGAP1, STXBP5, CD14, PCK1, STK11IP, LACC1, STAG2, NDUFAF3, CUTC, INTS11, ROBO1, SH3PXD2B, RAC2, SEC61B, KIF2C, C1QB, SCAND1, SLC31A1, SRP14, TEPSIN, KNTC1, HIST1H2AC, ACYP2, PPIH, SHARPIN, ANKZF1, CRIM1, ARHGAP25, TJAP1, GCLM, TNFAIP3, ARHGEF10, YWHAZ, ZMYND11, PRRG4, TNKS2, MAPK6* |
| DMRT1 | 184.87 | -66.79 | Inhibited | 64 | 9.68E-03 | 3.41E-02 | *NCKAP1, SLC4A4, DDIT3, BLOC1S6, IRF2BP2, NFATC4, TRMT112, PCMTD1, ANXA4, PPARA, RAPGEF5, AHNAK, PSMB2, LAMTOR2, PLOD2, SMCHD1, CDH2, UTRN, UBR3, FBXO44, ATXN7, FOXN2, LSP1, HM13, SPAG9, FOXJ3, DDHD2, PTPN12, OXR1, ATOX1, KLF6, JMJD1C, SOX6, TNS3, PLPP3, HLTF, STAG2, CALM3, ROBO2, GUK1, STBD1, ZDHHC17, NME1, CD47, YAP1, SATB1, NPM3, RTL6, SSC4D, NR3C1, GPRIN1, RPP21, RPS19BP1, DLG5, BRWD1, ADGRE5, ARHGEF10, YWHAZ, LATS2, KDM7A, HLF, USP12, PSMB10, CDK2AP2* |
| GATA2 | 197.30 | -110.39 | Inhibited | 65 | 1.00E-02 | 3.49E-02 | *NCKAP1, SPI1, IER2, NEBL, IRF2BP2, FOSL2, EEA1, TUT1, RAPGEF5, AHNAK, KIDINS220, LAPTM5, BBOX1, SLMAP, GOLIM4, FADS2, MAP2K3, SLC9A2, UTRN, FECH, UBR3, DHCR24, FOXN2, SERINC5, GPX1, SORD, MRM1, SPAG9, CYB5A, CDC42BPA, KLF6, KLF9, POLR3H, PDK1, PTDSS2, HLTF, STXBP5, CD14, PCK1, APOB, CITED4, STBD1, RAC2, KIF2C, KIAA1324L, SBF2, ACYP2, DOK2, GPRIN1, SELENOP, DHRS7B, ARHGEF37, DENND4A, DLG5, PICALM, TNFAIP3, DDX41, PPP1R9B, ADGRE5, YWHAZ, RABEP2, ALDH1A1, CAT, KDM7A, HLF, USP12, SLC50A1, WEE1* |
| HIF1A | 145.96 | -87.85 | Inhibited | 66 | 1.05E-02 | 3.63E-02 | *SMC2, NAMPT, HDHD5, SUMF1, AHNAK, PLOD2, UTRN, INSIG2, PDK1, MRPS18A, GUK1, SLC31A1, ANKZF1, CLN5, MAPK6* |
| MITF | 177.93 | -50.03 | Inhibited | 67 | 1.07E-02 | 3.65E-02 | *NCKAP1, SLC4A4, NAMPT, WDR91, MPV17, IER2, CHID1, VPS18, HDHD5, BIN3, RUFY3, DDIT3, FDX2, PRKD2, UBA52, RPN1, ANAPC11, BLOC1S6, WDR70, TOE1, IRF2BP2, RMDN2, NR1D2, FOSL2, NFATC4, VANGL1, ADI1, MMS19, PYGL, EEA1, TRMT112, ANXA4, ANXA5, SUMF1, KAT2B, GPRC5A, AEBP2, PPARA, PLCB1, AHNAK, APOLD1, PSMB2, RNF138, CHN2, SC5D, BID, SF3A2, RNLS, LAPTM5, SLMAP, VMA21, LAMTOR2, LAMTOR5, GOLIM4, MSI1, IL1RAP, FADS2, MAP2K3, STX7, CNPY2, CLIP1, FKBP9, SNRNP25, SNRPA, FECH, UBR3, RLF, FBXO44, AMDHD1, ATXN7, AMDHD2, DHCR24, G6PC3, SERINC5, RAD9A, ZNF771, ISY1, CELF4, GPX1, REXO4, EVC, PCCA, MET, ROCK2, UNC93B1, HM13, SH3BP2, FNIP1, SPAG9, BICC1, ATOX1, KLF5, NAGK, KLF9, POLR3H, TRMT61A, EIF4A2, PANK3, SOX6, TNS3, CPT1A, ACTN2, ALG5, MRPS18A, PLPP3, CFTR, SNX4, PTDSS2, SYMPK, STXBP5, PCK1, VAT1, STAG2, CITED4, ROBO1, SH3PXD2B, ZDHHC17, VAV1, NME1, APRT, RPUSD4, CD47, YAP1, TYMS, NT5C, PES1, SCAND1, SLC31A1, WASHC4, ARHGAP42, GPRIN3, SCNN1D, GPRIN1, COX19, CCNB1, CBX8, DENND4A, ARHGAP25, DLG1, BRWD1, PICALM, CHD9, TNFAIP3, ARHGEF10, ETV5, TRAPPC6A, LATS2, RABEP2, ACOX1, CAT, HLF, TBRG4, RMND5A, TNKS2, MARVELD2, PPP2R3C, MVB12A, STOM, MAPK6, CMTM4, MAPK12, WEE1, CDK2AP2, SNTB1* |
| IRF8 | 162.34 | 33.49 | Activated | 68 | 1.39E-02 | 4.64E-02 | *NAMPT, RUFY3, AEBP2, BID, PSMB8, RAD9A, LSP1, REXO4, TSSC4, GTF3C5, NCLN, PTPN18, ATOX1, EIF4A2, CUTC, IDE, ZFX, ZDHHC17, AIF1, ARHGAP25, YWHAG, ZMYND11, PSMB10* |
| TP53 | 173.54 | -26.72 | Inhibited | 69 | 1.42E-02 | 4.65E-02 | *UBA52, TOE1, IRF2BP2, MTMR10, EEA1, AEBP2, APOLD1, LAPTM5, SNX15, MAP3K20, MDM2, SGCB, GPX1, EVC, TP53INP1, POLR2I, SPAG9, REC8, SMARCB1, EFR3A, RPL36AL, PREB, CFTR, YAP1, TYMS, SCAND1, IL33, SBF2, ANKZF1, CHD9, DDX41, ENOSF1, LATS2, STOM* |
| HSF1 | 183.49 | 3.39 | Activated | 70 | 1.47E-02 | 4.76E-02 | *VPS18, NR0B2, MRPL38, ING1, NUDC, ANXA4, HEATR5A, HSPA1A, PLOD2, UTRN, MAP3K20, GOT1, COASY, UNC93B1, NCLN, CYB5A, EIF4A2, TNS3, IMP4, MYO5C, NME1, SSC4D, KNTC1, TYROBP, BRMS1, KLHL26, YWHAG, ZMYND11, PRRG4, CMTM4* |
| MEIS1 | 152.71 | 63.87 | Activated | 71 | 1.50E-02 | 4.79E-02 | *SPI1, GMFG, SMC4, KRTCAP2, WDR91, CDK5RAP3, BIN3, DDIT3, PRKD2, ABCB8, MCEE, KAT2B, PSMB2, KIDINS220, LAPTM5, MAP2K3, UBR3, CORO1A, AMDHD2, DHCR24, RAD9A, MRPL4, PSTPIP1, TSSC4, CXXC1, GLRX, POLR2I, SEC11C, EIF4A2, PDK1, CPT1A, FNDC4, CUTC, RAC2, VAV1, MICU3, AIF1, SLC31A1, ZGPAT, DOK2, ANKZF1, CBX8, HOOK3, PICALM, PEX1* |
| TCF3 | 192.12 | -115.04 | Inhibited | 72 | 0.01579 | 0.05 | *NCKAP1, UBA52, RPL28, VANGL1, RNF138, SLMAP, GOLIM4, SLC9A2, ATXN7, FOXN2, BFSP1, CPD, HSD17B11, SPAG9, SLC35A3, GFER, KLF5, PDK1, FNDC4, STK38L, B4GALNT1, VAT1, CITED4, ZFX, CD47, DOK2, CCNE2, KLHL13, COBLL1, GCLM, BRWD1, YWHAG, YWHAZ, LATS2, HLF, RMND5A, MAPK6, TMEM30B, ATP2B1, WEE1, SNTB1* |
