# Supplementary material for: Unique adaptations in neonatal hepatic transcriptome, nutrient signaling, and one-carbon metabolism in response to feeding ethyl cellulose rumen-protected methionine during late-gestation in Holstein cows
Source: BMC Genomics. 2021 Apr 17;22:280. doi: 10.1186/s12864-021-07538-w (PMC8053294; doi:10.1186/s12864-021-07538-w)
Supplement: Supplementary file 4 — Additional File 4:. Extended discussion on methionine synthase (MTR) and cystathionine beta-synthase (CBS) activity, phosphatidylcholine (PC) and very low density lipoproteins (VLDL), and on single genes involved in immune status in MET calves. [file 12864_2021_7538_MOESM4_ESM.docx]

**Additional File 4:** Extended discussion on methionine synthase (MTR) and cystathionine beta-synthase (CBS) activity, on phosphatidylcholine (OC) and very low density lipoproteins (VLDL), and on single genes involved in immune status in MET calves.

**Methionine synthase (MTR) activity**

At least in non-ruminants, within the one-carbon metabolism system, there is a phenomenon known as ‘folate trapping’ relying on irreversible conversion of 5,10-methyl-THF to 5-methyl-THF [1]. This indicates that conversion of 5-methyl-THF back into THF encompasses the release of the methyl group to successfully convert adenosylcobalamin into methylcobalamin. If this reaction does not occur, folate will get ‘trapped’ in the methylated form [1]. A pivotal role in this conversion is played by 5-Methyltetrahydrofolate-Homocysteine Methyltransferase Reductase (MTRR), which is known to maintain activation of the methionine synthase enzyme (MTR) [2]. Another potential factor associated with MTR activity at this early age could have been the supply, or lack, of vitamin B_12_. Although all calves consumed the same amounts of milk replacer and had ad-libitum access to solid feed, ruminal microbial activity at 4 days of age would not be expected to be the main supplier of B vitamins. Thus, it is unlikely that differences in activity of MTR were due to a shortage of vitamin B_12_, a key cofactor of MTR. Taken previous and current data together, we speculate that despite the lower activity of MTR in liver from MET calves, enzymatic efficiency might have been greater.

**Phosphatidylcholine (PC) and very low density lipoproteins (VLDL)**

Phosphatidylcholine (PC) is the major phospholipid component of very low density lipoproteins (VLDL) [3] and a strong reduction of VLDL secretion was observed when PC biosynthesis was inhibited [4]. In particular, along with PC synthesis in the CDP–choline pathway through choline kinase enzymes [5], in vivo synthesis of PC also occurs via SAM donating 3 methyl groups to phosphatidylethanolamine, a reaction catalyzed by PEMT [6]. This scenario, with the caveat that FA influx into liver would be from dietary origin, seemed to be further supported in our experiment by the strong upregulation of *SLC44A4* (*Solute Carrier Family 44 Member 4*) [FC = 4.46] encoding a sodium-dependent transmembrane transport protein involved in the uptake of choline for PC synthesis [7]. In this regard, possible effects on increased VLDL export through the generation of PC via the CDP pathway have already been demonstrated in primary bovine liver cells in response to enhanced in vitro supply of Met and choline [8].

The idea that an increase in VLDL could have been one route to maintain a ‘normal’ intracellular pool of FA and lipid droplets in MET calves was further and indirectly supported in our experiment by the greater concentrations of glycochenodeoxycholic, glycocholic, taurocholic, and lithocholic acids. Cholic and chenodeoxycholic acid are primary bile acids formed in the liver where they are conjugated with glycine or taurine before secretion into bile [9]. The pivotal role of bile in mammalian digestion and absorption of lipid is widely-recognized [10]. Since feed intake in our calves was the same, the fact that most of the bile acids had greater concentrations in MET calves suggested they were more efficient at digesting dietary fat from milk replacer. As such, MET calves might have cleared more FA from chylomicron remnants for further use either in FA oxidation, phospholipid synthesis, cellular membrane synthesis, and/or export as VLDL. The fact that the ‘FA degradation’ pathway was downregulated in MET calves suggested that, rather than active FA oxidation, incoming FA might have been incorporated into phospholipids (e.g. in bile), cellular membranes, and/or exported as VLDL. In this regard, it is noteworthy to highlight the significant upregulation of *SLC51A* [FC = 4.06] encoding the solute carrier family 51 subunit alpha. This transporter is essential for intestinal bile acid absorption and, thus, dietary lipid digestion [11]. The *NR0B2* gene [FC = 2.64], which encodes a member of the nuclear receptor superfamily, classified as an ‘orphan’ subgroup is also known for its role in hepatic bile acid metabolism [12].

The downregulation of *Lipase H* (*LIPH*) [FC = -1.75] further supported the idea of a better capacity of MET calves to handle dietary fat, and was in line with the lower expression of *Phospholipid Phosphatase 3* (*PLPP3*) [FC = -1.84]. Indeed, *LIPH* encodes a membrane-bound member of the mammalian triglyceride lipase family that catalyzes the production of 2-acyl lysophosphatidic acid (LPA) [13], whereas *PLPP3* encodes a cell-surface glycoprotein that hydrolyzes extracellular LPA and short-chain phosphatidic acid [14].

In the context of VLDL synthesis the downregulation of *Angiopoietin-Like Protein 3* (*ANGPTL3*) gene [FC = -1.55] in MET calves was noteworthy. Considering that ANGPTL3 has been shown to inhibit lipoprotein lipase (LPL) [15], which is an endothelium-associated enzyme that hydrolyzes the triacylglycerol component of circulating chylomicrons and VLDL [16], we speculate a possible role of *ANGPTL3* in the modulation of lipid metabolism by regulation of VLDL triglyceride levels through inhibition of LPL activity. In this general scenario, the unexpected downregulation of *Apolipoprotein B* (*APOB*) [FC = -1.29], although in line with downregulation of PPARA [FC = -1.33] which in non-ruminants controls its expression [17], deserved particular mention. It is commonly-accepted that apoB is important in stabilizing the nascent VLDL particles [18]. Single gene expression studies with dairy cows have suggested that decreased mRNA abundance for *APOB* is consistent with decreased secretion of VLDL from liver [19, 20]. However, expression patterns of key genes involved in VLDL assembly in bovine vary greatly early postpartum, and these contrasting responses do not clarify how the entire process might be regulated at the transcriptional level [19]. Considering the downregulation of *APOB* in our experiment, we speculate that the limiting step of VLDL synthesis in calves is not at the level of APOB. For instance, betaine (a methyl donor) alleviated hepatic triglyceride accumulation in apolipoprotein E-deficient mice, upregulating the expression of PPARA and some of its target genes (e.g. *CPT1A*) and reversing the hypermethylation of PPAR via reducing methylation of its promoter [21].

At least in non-ruminants, it is well known that hepatic VLDL production is tightly regulated by insulin. Indeed, in response to increased postprandial insulin release, hepatic VLDL production is suppressed whereas under fasting conditions, an increased VLDL secretion into the blood is observed [22]. Although the protein abundance data in the present study suggested greater hepatic insulin sensitivity in MET calves, it is unclear if this effect was strong enough to diminish VLDL production in those calves. The role of forkhead transcription factor FOXO1 to integrate hepatic insulin action to VLDL production in non-ruminants is well-established [23], and was recognizable among our TF enrichment analysis results where a high impact and inhibited state were predicted for FOXO1 (Table 10).

**Cystathionine beta-synthase (CBS) activity**

The cystathionine beta-synthase (CBS) enzyme catalyzes the rate-limiting step in the transsulfuration pathway, the condensation of homocysteine and serine to cystathionine. Cystathionine γ-lyase subsequently catalyzes the hydrolysis of cystathionine to cysteine and α-ketobutyrate. Apart from its role in protein synthesis, cysteine is a precursor of glutathione (GSH), a strong antioxidant and an essential compound in detoxification of many xenobiotics [24, 25]. Short-term regulation of GSH production occurs via the availability of cysteine, the limiting substrate, and by feedback inhibition of glutamate–cysteine ligase (GCL) by GSH [26]. In this regard, we detected downregulation of *GCLM* [FC = -1.33], a GCL modifier subunit, whose upregulation in rats was reported in response to cysteine deprivation and was associated with a higher catalytic efficiency of GCL [26]. It is also important to note that restoration of GSH levels in hepatocytes from rats reversed the inactivation of MAT resulting from S-nitrosylation [27], a response we speculate is supported by the tendency for greater *MAT1A* abundance in MET calves.

Despite continued progress on epigenetic mechanisms in mammals, epigenetic control of CBS is unclear with few published studies mainly in non-ruminants [28–31]. For example, altered intrauterine milieu associated with uteroplacental insufficiency could determine genomic hypomethylation in the liver, inducing decreased mRNA levels of CBS [29]. Decreased expression of CBS has also been observed in mice fed a methionine-deficient diet [32, 33], and lower CBS activity detected when betaine homocysteine methyltransferase (BHMT) is inhibited [34]. Supplemental betaine restored CBS activity in BHMT knock-out mice [35], but it is also reported that reduction in the SAM/SAH ratio due to loss of CBS activity, does not result in overall DNA hypomethylation [30]. These seemingly contrasting results, mostly from non-ruminant species, underscore important gaps in knowledge and the need for deeper investigation.

**Single genes involved in immune status in MET calves**

In context of an enhanced immune status in MET calves, focusing on single gene targets, a prominent role seemed to be played by *CLEC6A* (*C-Type Lectin Domain Containing 6A*) [FC=2.30; Table 8] and *CD209* (*CD209 molecule*) [FC=2.02; Table S2] genes both of which were markedly upregulated in our analysis. These two genes belong to C-type lectin receptors [36]. In particular, *CD209* is described as a phagocytic receptor [37]. Also upregulation of *CD14* (*c*) [FC = 1.62] deserves particular mention. Indeed, this gene is known to influence host immune response and alternative activation of macrophages [38]. A plausible better immune status was also suggested by the downregulation of genes usually associated with an ongoing inflammation state. For example, downregulation of *TNFAIP3* (*TNF Alpha Induced Protein 3*) [FC = -1.61] is compatible with a lower basal inflammatory status, considering that this gene is currently qualified as a susceptibility gene for inflammatory disease [39] and that a high level of TNF-α was observed in calves from dams exposed to high concentrations of metabolic-related oxidative stressors (e.g. NEFA) [40]. The downregulation of *NR3C1* (*Nuclear Receptor Subfamily 3 Group C Member 1*) [FC = -1.40] also supported our hypothesis, since this gene is associated with the mitigation of inflammatory activation in vitro [41].

**References**

1. Pizano JM, Williamson CB. Nutritional Influences on Methylation. In: Noland D, Drisko JA, Wagner L, editors. Integrative and Functional Medical Nutrition Therapy: Principles and Practices. Cham: Springer International Publishing; 2020. p. 269–84. doi:10.1007/978-3-030-30730-1_18.

2. Wang W, Jiao X-H, Wang X-P, Sun X-Y, Dong C. MTR, MTRR, and MTHFR gene polymorphisms and susceptibility to nonsyndromic cleft lip with or without cleft palate. Genet Test Mol Biomarkers. 2016;20:297–303.

3. Gruffat D, Durand D, Graulet B, Bauchart D. Regulation of VLDL synthesis and secretion in the liver. Reprod Nutr Dev. 1996;36:375–89.

4. Yao ZM, Vance DE. The active synthesis of phosphatidylcholine is required for very low density lipoprotein secretion from rat hepatocytes. J Biol Chem. 1988;263:2998–3004.

5. Fagone P, Jackowski S. Phosphatidylcholine and the CDP–choline cycle. Biochimica et Biophysica Acta (BBA) - Molecular and Cell Biology of Lipids. 2013;1831:523–32.

6. Zhou Z, Garrow TA, Dong X, Luchini DN, Loor JJ. Hepatic activity and transcription of betaine-homocysteine methyltransferase, methionine synthase, and cystathionine synthase in periparturient dairy cows are altered to different extents by supply of methionine and choline. J Nutr. 2017;147:11–9.

7. Paul A, Krelin Y, Arif T, Jeger R, Shoshan-Barmatz V. A new role for the mitochondrial pro-apoptotic protein SMAC/Diablo in phospholipid synthesis associated with tumorigenesis. Molecular Therapy. 2018;26:680–94.

8. Zhou YF, Zhou Z, Batistel F, Martinez-Cortés I, Pate RT, Luchini DL, et al. Methionine and choline supply alter transmethylation, transsulfuration, and cytidine 5′-diphosphocholine pathways to different extents in isolated primary liver cells from dairy cows. Journal of Dairy Science. 2018;101:11384–95.

9. Chiang JYL. Bile acid metabolism and signaling. In: Comprehensive Physiology. American Cancer Society; 2013. p. 1191–212. doi:10.1002/cphy.c120023.

10. de Aguiar Vallim TQ, Tarling EJ, Edwards PA. Pleiotropic roles of bile acids in metabolism. Cell Metab. 2013;17:657–69.

11. Ballatori N, Christian WV, Wheeler SG, Hammond CL. The heteromeric organic solute transporter, OSTα–OSTβ/SLC51: A transporter for steroid-derived molecules. Mol Aspects Med. 2013;34. doi:10.1016/j.mam.2012.11.005.

12. Chanda D, Park J-H, Choi H-S. Molecular basis of endocrine regulation by orphan nuclear receptor small heterodimer partner. Endocrine Journal. 2008;55:253–68.

13. Jin W, Broedl UC, Monajemi H, Glick JM, Rader DJ. Lipase H, a new member of the triglyceride lipase family synthesized by the intestine. Genomics. 2002;80:268–73.

14. Busnelli M, Manzini S, Hilvo M, Parolini C, Ganzetti GS, Dellera F, et al. Liver-specific deletion of the Plpp3 gene alters plasma lipid composition and worsens atherosclerosis in apoE-/- mice. Sci Rep. 2017;7:44503.

15. Liu J, Afroza H, Rader DJ, Jin W. Angiopoietin-like protein 3 inhibits lipoprotein lipase activity through enhancing its cleavage by proprotein convertases. J Biol Chem. 2010;285:27561–70.

16. Mead JR, Irvine SA, Ramji DP. Lipoprotein lipase: structure, function, regulation, and role in disease. J Mol Med (Berl). 2002;80:753–69.

17. Shah A, Rader DJ, Millar JS. The effect of PPAR-α agonism on apolipoprotein metabolism in humans. Atherosclerosis. 2010;210:35–40.

18. Gibbons GF. Assembly and secretion of hepatic very-low-density lipoprotein. Biochem J. 1990;268:1–13.

19. Bernabucci U, Ronchi B, Basiricò L, Pirazzi D, Rueca F, Lacetera N, et al. Abundance of mRNA of apolipoprotein B100, apolipoprotein E, and microsomal triglyceride transfer protein in liver from periparturient dairy cows. Journal of Dairy Science. 2004;87:2881–8.

20. Sparks JD, Collins HL, Chirieac DV, Cianci J, Jokinen J, Sowden MP, et al. Hepatic very-low-density lipoprotein and apolipoprotein B production are increased following in vivo induction of betaine–homocysteine S-methyltransferase. Biochem J. 2006;395 Pt 2:363–71.

21. Wang L, Chen L, Tan Y, Wei J, Chang Y, Jin T, et al. Betaine supplement alleviates hepatic triglyceride accumulation of apolipoprotein E deficient mice via reducing methylation of peroxisomal proliferator-activated receptor alpha promoter. Lipids Health Dis. 2013;12:34.

22. Malmström R, Packard CJ, Watson TD, Rannikko S, Caslake M, Bedford D, et al. Metabolic basis of hypotriglyceridemic effects of insulin in normal men. Arterioscler Thromb Vasc Biol. 1997;17:1454–64.

23. Kamagate A, Dong HH. FoxO1 integrates insulin signaling to VLDL production. Cell Cycle. 2008;7:3162–70.

24. Pastore A, Federici G, Bertini E, Piemonte F. Analysis of glutathione: implication in redox and detoxification. Clinica Chimica Acta. 2003;333:19–39.

25. Gaucher C, Boudier A, Bonetti J, Clarot I, Leroy P, Parent M. Glutathione: antioxidant properties dedicated to nanotechnologies. Antioxidants (Basel). 2018;7. doi:10.3390/antiox7050062.

26. Lee J-I, Kang J, Stipanuk MH. Differential regulation of glutamate–cysteine ligase subunit expression and increased holoenzyme formation in response to cysteine deprivation. Biochem J. 2006;393 Pt 1:181–90.

27. Corrales FJ, Ruiz F, Mato JM. In vivo regulation by glutathione of methionine adenosyltransferase S-nitrosylation in rat liver. Journal of Hepatology. 1999;31:887–94.

28. Caudill MA, Wang JC, Melnyk S, Pogribny IP, Jernigan S, Collins MD, et al. Intracellular S-adenosylhomocysteine concentrations predict global DNA hypomethylation in tissues of methyl-deficient cystathionine beta-synthase heterozygous mice. J Nutr. 2001;131:2811–8.

29. MacLennan NK, James SJ, Melnyk S, Piroozi A, Jernigan S, Hsu JL, et al. Uteroplacental insufficiency alters DNA methylation, one-carbon metabolism, and histone acetylation in IUGR rats. Physiological Genomics. 2004;18:43–50.

30. Lee H-O, Wang L, Kuo Y-M, Gupta S, Slifker MJ, Li Y, et al. Lack of global epigenetic methylation defects in CBS deficient mice. J Inherit Metab Dis. 2017;40:113–20.

31. Zhang N. Role of methionine on epigenetic modification of DNA methylation and gene expression in animals. Anim Nutr. 2018;4:11–6.

32. Tang B, Mustafa A, Gupta S, Melnyk S, James SJ, Kruger WD. Methionine-deficient diet induces post-transcriptional down-regulation of Cystathionine β-Synthase. Nutrition. 2010;26:1170–5.

33. Aissa AF, Tryndyak V, Conti A de, Melnyk S, Gomes TDUH, Bianchi MLP, et al. Effect of methionine-deficient and methionine-supplemented diets on the hepatic one-carbon and lipid metabolism in mice. Molecular Nutrition & Food Research. 2014;58:1502–12.

34. Strakova J, Gupta S, Kruger WD, Dilger RN, Tryon K, Li L, et al. Inhibition of betaine-homocysteine S-methyltransferase in rats causes hyperhomocysteinemia and reduces liver cystathionine β-synthase activity and methylation capacity. Nutr Res. 2011;31:563–71.

35. Kim SK, Choi KH, Kim YC. Effect of acute betaine administration on hepatic metabolism of S-amino acids in rats and mice. Biochemical Pharmacology. 2003;65:1565–74.

36. Patin EC, Orr SJ, Schaible UE. Macrophage inducible C-type lectin as a multifunctional player in immunity. Front Immunol. 2017;8. doi:10.3389/fimmu.2017.00861.

37. Kerrigan AM, Brown GD. C-type lectins and phagocytosis. Immunobiology. 2009;214:562–75.

38. Tundup S, Srivastava L, Nagy T, Harn D. CD14 influences host immune responses and alternative activation of macrophages during Schistosoma mansoni infection. Infect Immun. 2014;82:3240–51.

39. Jarosz M, Olbert M, Wyszogrodzka G, Młyniec K, Librowski T. Antioxidant and anti-inflammatory effects of zinc. Zinc-dependent NF-κB signaling. Inflammopharmacol. 2017;25:11–24.

40. Ling T, Hernandez-Jover M, Sordillo LM, Abuelo A. Maternal late-gestation metabolic stress is associated with changes in immune and metabolic responses of dairy calves. Journal of Dairy Science. 2018;101:6568–80.

41. Abdelmegeid MK, Vailati-Riboni M, Alharthi A, Batistel F, Loor JJ. Supplemental methionine, choline, or taurine alter in vitro gene network expression of polymorphonuclear leukocytes from neonatal Holstein calves. Journal of Dairy Science. 2017;100:3155–65.
