# Supplementary material for: Unique adaptations in neonatal hepatic transcriptome, nutrient signaling, and one-carbon metabolism in response to feeding ethyl cellulose rumen-protected methionine during late-gestation in Holstein cows
Source: BMC Genomics. 2021 Apr 17;22:280. doi: 10.1186/s12864-021-07538-w (PMC8053294; doi:10.1186/s12864-021-07538-w)
Supplement: Supplementary file 5 — Additional File 5: KEGG pathways in ‘Lipid Metabolism’, ‘Amino Acid Metabolism’, and ‘Immune System’ subcategories resulting from the DIA analysis of RNAseq data from liver tissue of 4-d old Holstein calves (n = 6/group) born to cows randomly assigned to receive a basal control (CON) diet from − 28 ± 2 d to parturition [1.47 Mcal/kg dry matter (DM) and 15.3% crude protein (CP)] with no added Met or CON plus ethyl cellulose Met (MET, Mepron®, Evonik Nutrition & Care GmbH, Germany). The columns represent the effect (impact) and flux responses. The blue bars represent the effect value (0 to 300), and the flux columns represent negative (−) and positive (+) flux (− 300 to + 300) based on the direction of the effect. The positive flux (red bars) indicates an upregulation in treated (MET) liver tissue cells compared to control (CON) ones, while the negative flux (green bars) indicates a downregulation. [file 12864_2021_7538_MOESM5_ESM.docx]

**Additional File 5:** KEGG pathways in ‘Lipid Metabolism’, ‘Amino Acid Metabolism’, and ‘Immune System’ subcategories resulting from the DIA analysis on heifer calf liver transcriptome from Holstein cows (n = 6/treatment) randomly assigned to receive a basal control (CON) close-up diet (from −28 ±2 d to parturition) [1.47 Mcal/kg dry matter (DM) and 15.3% crude protein (CP)] with no added MET or CON plus ethyl cellulose MET (MET, Mepron®, Evonik Nutrition & Care GmbH, Germany). The columns represent the effect (impact) and flux responses. The blue bars represent the effect value (0 to 300), and the flux columns represent negative (−) and positive (+) flux (−300 to +300) based on the direction of the effect. The positive flux (red bars) indicates an upregulation in treated (MET) liver tissue cells compared to control (CON) ones, while the negative flux (green bars) indicates a downregulation.
