# Supplementary material for: Unique adaptations in neonatal hepatic transcriptome, nutrient signaling, and one-carbon metabolism in response to feeding ethyl cellulose rumen-protected methionine during late-gestation in Holstein cows
Source: BMC Genomics. 2021 Apr 17;22:280. doi: 10.1186/s12864-021-07538-w (PMC8053294; doi:10.1186/s12864-021-07538-w)
Supplement: Supplementary file 6 — Additional File 6:. Ingredient and nutrient composition of far-off (from − 45 to − 29 d) and close-up (from − 28 d to parturition) diets fed to Holstein cows used in the present study. [file 12864_2021_7538_MOESM6_ESM.docx]

**Additional File 6:** Ingredient and nutrient composition of far-off (from −45 to −29 d) and close-up (from −28 d to parturition) diets fed to cows.

| **Ingredient, % of DM** | **Diets** | |
| --- | --- | --- |
|  | **Far-off** | **Close-up** |
| Alfalfa haylage | ­­ — | 6.55 |
| Corn silage | 34.7 | 26.6 |
| Wheat straw | 33.7 | 26.5 |
| Corn grain, ground, dry | — | 12.6 |
| Molasses, beet sugar | — | 4.03 |
| Soybean hulls | 15.7 | 3.46 |
| Soybean meal, 48% CP | 12.0 | 7.83 |
| Expeller soybean meal^1^ | — | 5.80 |
| Protein supplement^2^ | — | 0.78 |
| Urea | 0.46 | 0.59 |
| Soychlor^3^ | — | 1.23 |
| Salt | 0.40 | — |
| Dicalcium phosphate | 0.50 | 0.52 |
| Magnesium sulfate | 1.90 | 2.08 |
| Mineral vitamin mix^4^ | 0.40 | 0.17 |
| Vitamin A^5^ | — | 0.03 |
| Vitamin D^6^ | — | 0.03 |
| Vitamin E^7^ | 0.40 | 0.60 |
| Biotin^8^ | — | 0.70 |
| Momensin^9^ | 0.01 | — |
| Ethyl-cellulose rumen-protected Met^10^ | — | 0.09 |

^1^SoyPlus, West Central Soy (Ralston, IA).

^2^ProVAAl AADvantage, Perdue AgriBusiness (Salisbury).

^3^West Central Soy.

^4^Contained a minimum of 5% Mg, 10% S, 7.5% K, 2.0% Fe, 3.0% Zn, 3.0% Mn, 5,000 mg of Cu/kg, 250 mg of I/kg, 40 mg of Co/kg, 150 mg of Se/kg, 2,200 kIU of vitamin A/kg, 660 kIU of vitamin D3/kg, and 7,700 IU of vitamin E/kg.

^5^Contained 30,000 kIU/kg.

^6^Contained 5,000 kIU/kg.

^7^Contained 44,000 kIU/kg.

^8^ADM Animal Nutrition (Quincy, IL).

^9^Rumensin, Elanco Animal Health (Greenfield, IN).

^10^Evonik Nutrition and Care GmbH (Hanau-Wolfgang, Germany). Added only in the treatment group.
