# Supplementary material for: Unique adaptations in neonatal hepatic transcriptome, nutrient signaling, and one-carbon metabolism in response to feeding ethyl cellulose rumen-protected methionine during late-gestation in Holstein cows
Source: BMC Genomics. 2021 Apr 17;22:280. doi: 10.1186/s12864-021-07538-w (PMC8053294; doi:10.1186/s12864-021-07538-w)
Supplement: Supplementary file 7 — Additional File 7:. Extended information about quantitative real-time PCR. Accession number, gene symbol, and forward and reverse primer sequences of genes analyzed in calf liver. [file 12864_2021_7538_MOESM7_ESM.docx]

**Additional File 7:** Extended information about quantitative real-time PCR. Accession number, gene symbol, and forward and reverse primer sequences of genes analyzed in calf liver.

**RNA Isolation, Quality Evaluation and cDNA synthesis**

Total RNA was isolated from liver samples using TRIzol Reagent (Invitrogen, Carlsbad, CA). Liver samples were homogenized in TRIzol reagent with 1 μL linear acrylamide (Ambion, Inc., Austin, TX) using a Tissue-TearorTM (BioSpec Products, Inc.) homogenizer at maximum speed. Upon centrifugation, total RNA was separated with chloroform followed by acid phenol:chloroform (Ambion, Inc., Austin, TX) to remove DNA. Total RNA was then precipitated with isopropanol and the RNA pellet was cleaned with 75% ethanol prior to reconstitution in RNA storage buffer (Ambion, Inc., Austin, TX) and storage at −80°C. Total RNA was cleaned using RNeasy mini kit columns (Cat. # 74104, QIAGEN) and genomic DNA was removed using the RNase-Free DNase Set (Cat. # 79254, Qiagen). The RNA concentration was measured with NanoDrop ND-1000 spectrophotometer (NanoDrop Technologies). The purity of RNA was assessed by ratio of optical density OD260/280, which were above 1.97 for all samples. A portion of the RNA was diluted to 100 ng/μL with DNase/RNase-free water for cDNA synthesis through RT-PCR. cDNA was synthesized using 100 ng of RNA, 1 μg of dT18 (Operon Biotechnologies), 1 μL of 10 mM dNTP mix (Cat. #18427013, Invitrogen), 1 μL of Random Primers (Cat. #48190011, Invitrogen), and 10 μL of DNase/RNase-free water. The mixture was incubated at 65°C for 5 min and kept on ice for 3 min. A total of 6 μL of master mix composed of 4.5 μL of 5X First-Strand Buffer, 1 μL of 0.1 M DTT, 0.25 μL (50 U) of SuperScript III RT (Cat. #11752050, Invitrogen), and 0.25 μL of RNase Inhibitor (Cat. #N2111, Promega) was added. The reaction was performed in an Eppendorf Mastercycler Gradient following such temperature program: 25°C for 5 min, 50°C for 60 min, and 70°C for 15 min. The cDNA was then diluted 1:4 with DNase/RNase-free water.

**Primer Design and Evaluation**

Primers were designed and evaluated as previously described (Bionaz and Loor, 2007). Briefly, primers were designed using Primer Express 3.0 with minimum amplicon size of 80 bp (amplicons of 100–120 bp were of superiority, if possible) and limited 3’ G + C percentage (Applied Biosystems). Primer sets were intentionally designed to fall across exon-exon junctions. Then, primers were aligned against NCBI database through BLASTN and UCSC’s COW (Bos taurus) Genome Browser Gateway to determine the compatibility of primers with already annotated sequence of the corresponding gene in both databases. Prior to quantitative real time PCR (qPCR), primers were verified through a 20-μL PCR reaction, which followed the same procedures of qPCR described below except the dissociation step. A universal reference cDNA amplified from all samples was utilized to ensure the identification of genes. Five microliters of PCR product was run in a 2% agarose gel stained with ethidium bromide, and the remaining 15 μL were cleaned with a QIAquick PCR Purification Kit (Cat. #28104, QIAGEN) and sequenced at the Core DNA Sequencing Facility of the Roy J. Carver Biotechnology Center at the University of Illinois, Urbana. The sequencing product was confirmed through BLASTN at the National Center for Biotechnology Information (NCBI) database. Only primers that presented a single band of the expected size and the right amplification product were used for qPCR. Supplemental Table 1 shows the list of all primers designed and used for qPCR analysis in this study.

**qPCR**

qPCR was performed in a MicroAmp Optical 384-Well Reaction Plate (Cat. #4309849, Applied Biosystems). Within each well, 4 μL of diluted cDNA combined with 6 μL of mixture composed of 5 μL 1×SYBR Green master mix (Cat. #4309155, Applied Biosystems), 0.4 μL each of 10 μM forward and reverse primers, and 0.2 μL of DNase/RNase-free water were added. Three replicates and a 6-point standard curve plus the nontemplate control (NTC) were run for each sample to test the relative expression level. qPCR was conducted in ABI Prism 7900 HT SDS instrument (Applied Biosystems) following the conditions below: 2 min at 50°C, 10 min at 95°C, 40 cycles of 15 s at 95°C (denaturation), and 1 min at 60°C (annealing + extension). The presence of a single PCR product was verified by the dissociation protocol using incremental temperatures to 95°C for 15 s, then 65 °C for 15 s. The threshold cycle (Ct) data were analyzed and transformed using the standard curve with the 7900 HT Sequence Detection System Software (version 2.2.1, Applied Biosystems, CA). Data were then normalized with the geometric mean of the three Internal Control Genes (ICG).

**Accession number, gene symbol, and forward and reverse primer sequences of genes analyzed in calf liver.**

| **Gene** | **Accession number** | **Forward**  **primer** | **Reverse**  **primer** |
| --- | --- | --- | --- |
| *MAT1A* | NM_001046497 | CAAGGGCTTTGACTTTAA | CCGACATCCTCTTCATTT |
| *BHMT* | NM_001011679 | GCTCTCCTCGTCCATCCTCAT | CCGTTCTAGGATGCCCTTCTT |
| *BHMT2* | XM_003586514 | ACACCACAAGGATGAAGT | CAGGCTCTCCAGATTCTT |
| *MTR* | NM_001030298 | ATACCGCCAATGCCAAGG | ATGAGACACGCTGATGACAA |
| *SAHH* | NM_001034315 | CAATGTCAATGACTCTGT | CTTGATGCCATCTATGAG |
| *PEMT* | NM_182989 | AATTACCAAGAGCAGAGG | CAGATTCCAGATTCCAGAT |
| *BADH* | NM_001045969 | CACCTACTGTCCTGCTAA | ATCACCAATCTGTCTCACTAC |
| *CHDH* | NM_001205564 | AAACTGAGAAGTGCCAAC | ACGGAAGTCTTTAATGTCA |
| *SARDH* | NM_001193041 | GAGGAGGTGTCAGATGAG | GCAGACTGTGGACTTAATC |
| *DNMT1* | NM_001206502.1 | GCGCCTCAGCTAAAATCAAGG | CCACAAACACCGCATACGAC |
| *DNMT3A* | NM_182651.2 | GGAGTCACTGGAAGCCCAACC | CATAGATCCAGGTGTGGAGCGG |
| *CBS* | NM_001102000 | GCCACCACCTCTGTCAAATTC | GGACAGAAAGCAGAGTGGTAACTG |
| *CTH* | BC151523.1 | GACCCATGCATCAGTGCCTA | ATTTGGAGGGTTAGCTGCCT |
| *CSAD* | XM_005206242 | GCCTCAACACCAGCCAGTA | GTTTCTTCAGCACCTCCTGCCT |
| *CDO1* | NM_001034465 | ATGGAAGCCTATGAGAGCAA | TTCAGAAAGCAGTGGGAGTC |
| *GCLC* | NM_001083674 | TACGATCAGTTGGCTACC | CCGAGTTCTATCATCTACAGA |
| *GSS* | NM_001015630 | CGAGTGATCCAATGCATTTCA | ATGTCCCACGTGCTTGTTCAT |
| *GSR* | NM_001114190 | GAGAACGCTGGCATTGAG | AGCAGGCAGTCAACATCT |
| *GPX1* | NM_174076.3 | AACGCCAAGAACGAGGAGATC | CATTCACCTCGCACTTTTCGA |
| *CHKA* | XM_002699402 | GCACAGGTTCCTCAGTTA | GCCATCCAGCAGTAAGAT |
| *CHKB* | XM_010805827 | CCAAGAGGAGCAGAGGAA | GTAGAACTGGAACCGAGACT |
| *PCYT1A* | XM_005201384 | GATGAGGTGGTGAGGAAT | CGAGTGATGATGTCTGATG |
| *PCYT1B* | NM_001193051 | ACTGTCGCTATGTGGATGAA | GCCTTCTGTTCTCTGTGTTG |
| *CEPT1* | NM_001193130 | GGATAGCCCTGGTCTTCTCTT | ATTCGCCTCAATATGTTCAGATTCTT |

**References**

Bionaz M, Loor JJ. Identification of reference genes for quantitative real-time PCR in the bovine mammary gland during the lactation cycle. Physiol Genomics. 2007 May 11;29(3):312-9. doi: 10.1152/physiolgenomics.00223.2006. Epub 2007 Feb 6. PMID: 17284669.
